# NXPH4 Promotes Gemcitabine Resistance in Bladder Cancer by Enhancing Reactive Oxygen Species and Glycolysis Activation through Modulating NDUFA4L2

**DOI:** 10.3390/cancers14153782

**Published:** 2022-08-03

**Authors:** Decai Wang, Pu Zhang, Zijian Liu, Yifei Xing, Yajun Xiao

**Affiliations:** 1 Department of Urology Surgery, Union Hospital, Tongji Medical College, Huazhong University of Science and Technology, 1277 Jiefang Avenue, Wuhan 430022, China; d202081775@hust.edu.cn (D.W.); d202081801@hust.edu.cn (P.Z.); 2 Department of Radiation Oncology, Cancer Center and State Key Laboratory of Biotherapy, West China Hospital, Sichuan University, Chengdu 610041, China; 2020324025312@stu.scu.edu.cn

**Keywords:** NXPH4, bladder cancer, bioinformatics, drug resistance, prognosis

## Abstract

**Simple Summary:**

Bladder cancer is one of the most common malignant tumors of the urinary system, and its treatment is mainly surgical resection, supplemented by chemotherapy after surgery. Postoperative chemotherapy can significantly reduce the tumor recurrence rate and improve the prognosis of bladder cancer patients. However, chemotherapy resistance is one of the major challenges in the treatment of bladder cancer. Therefore, we conducted a series bioinformatic analyses and functional experiments to reveal the novel role of NXPH4 in bladder cancer. We found NXPH4 not only influenced the proliferation, migration, invasion ability of cancer cells, but also affected the level of glycolysis and reactive oxygen species and further promoted the gemcitabine resistance of bladder cancer. Our research has found a novel molecule that may play an important part in the neoplasia, which may provide another angle in the treatment of bladder cancer.

**Abstract:**

Bladder cancer is one of the most prevalent kinds of cancer worldwide, and resistance to gemcitabine is a major problem for patients. The pathogenesis of bladder cancer and mechanism of resistance to chemotherapy remain to be explored. Through bioinformatics analysis, we first found that NXPH4 was independently related to the prognosis of patients with bladder cancer. Through wound healing assays, transwell invasion assays, and plate clone formation assays, we found that NXPH4 promoted the proliferation, migration, and invasion of bladder cancer cells. The induced gemcitabine resistance cell line also showed a higher expression of NXPH4. A glycolytic activity assay demonstrated that the expression of NXPH4 was positively related to glycolysis. A higher level of reactive oxygen species caused by enhanced levels of NXPH4 was found in gemcitabine-resistant cell lines. NDUFA4L2, glycolysis, and reactive oxygen species were shown to be essential for NXPH4-regulated functions through rescue assays in cell lines. The roles of NXPH4-regulated glycolysis, gemcitabine resistance, and NDUFA4L2 were validated in vivo as well. Our results imply that NXPH4 contributes to the proliferation, migration, and invasion of bladder cancer by maintaining the stability of NDUFA4L2 and consequently activating reactive oxygen species and glycolysis.

## 1. Introduction

As one of the most prevalent kinds of cancers, bladder cancer is diagnosed in 430,000 patients and is reported to cause 170,000 deaths annually around the world, causing a heavy burden for both patients and countries [1]. Tumors are divided into nonmuscle-invasive bladder cancer (NMIBC) and muscle-invasive bladder cancer (MIBC) according to the location of the tumor cells [2]. Owing to the development of sequencing technology in recent decades, our treatments for bladder cancer have become more personalized and effective. Additionally, advancements in sequencing technology are deepening our understanding of the pathogenesis of bladder cancer and leading us to focus more on targeted therapies based on molecular characterization [3]. However, to date, while the five-year overall survival rate for patients with NMIBC is about 90%, most patients experience recurrence after one–two years of treatment, decreasing their quality of life. Additionally, the five-year overall survival rate for patients with MIBC is only about 60%, which may not be satisfactory. Therefore, new targeted therapy intended to increase the overall survival rate and decrease complications is essential. Neurexophilin-4 (NXPH4) is one member of the neurexophilin family that also includes NXPH1, NXPH2, and NXPH3. They all share a common structure consisting of five domains [4,5]. The function of NXPH4 has rarely been studied in recent decades [6,7]. Through bioinformatic analysis, we first found that it was strongly associated with the prognosis of patients with bladder cancer. Therefore, we further conducted experiments to explore the underlying mechanism of the influence of NXPH4 on bladder cancer.

## 2. Materials and Methods

### 2.1. Sample Selection

From September 2020 to May 2021, 34 pairs of bladder cancer tissues and adjacent normal tissues were collected from confirmed patients in Wuhan Union Medical College Hospital. All tissue samples were stored in liquid nitrogen for subsequent studies. All tissue samples were verified by pathologists from Wuhan Union Medical College Hospital. All patients and their families signed informed consent forms, and this study was approved by the hospital ethics committee (approval number: I2020I IEC-J (022)).

### 2.2. Data Collection and Processing

We downloaded various kinds of transcriptome data on different cancers from The Cancer Genome Atlas (TCGA) in count format. Using R tools, we transformed the format into transcripts per kilobase of exon model per million mapped reads. In addition, the corresponding clinical and prognostic information including the age, race, overall survival, disease-free survival, and progression-free survival time of patients with bladder cancer was also obtained. The duplicated samples were removed in order to increase the accuracy of the analysis results.

### 2.3. The Baseline Data of Patients with Bladder Cancer from TCGA

We calculated the numbers and proportions of the patients with bladder cancer from different T stages, N stages, M stages, pathologic stages, radiation therapy, primary therapy outcomes, races, ages, histologic grades, and subtypes. We present the results in Table S1.

### 2.4. The Exploration of the Expression Levels of NXPH4 in Patients with Different Status

Utilizing the processed transcriptome and clinical data of the TCGA-BLCA cohort, we contrasted the expression levels of NXPH4 in bladder cancer tissues with non-paired and paired normal bladder tissues. Moreover, we investigated the differential expression levels of NXPH4 in patients with diverse cancers and corresponding normal tissues. The difference level was measured in patients from the TCGA-BLCA cohort with distinct survival outcomes, which indicated the results of overall survival, progression-free survival, and disease-specific survival. We also investigated the correlation between the expression of NXPH4 and the races, and primary therapy outcomes of patients with bladder cancer. R package ggplot2 was used for the visualization of the discrepant expression levels of NXPH4.

### 2.5. The Prognostic Value of NXPH4, Gene Set Enrichment Analysis, and the Infiltration Level of Immune Cells

We performed Kaplan–Meier analyses to study the possible influence of NXPH4 on the results of the survival of patients with bladder cancer from TCGA. According to the median value of the expression levels of NXPH4, the patients were divided into two groups—the NXPH4-Low and NXPH4-High groups. We contrasted the overall survival, progression-free survival and disease-specific survival of patients in these groups with the R packages survminer and survival. Owing to the limitations of the Kaplan–Meier analyses, we proceeded to conduct univariate and multivariate Cox analyses to confirm the prognosis value of NXPH4. After dividing the patients with bladder cancer into two groups through the expression of NXPH4, we conducted gene set enrichment analyses with the R package clusterProfiler. The infiltration levels of various kinds of immune cells between NXPH4-Low and NXPH4-High group patients were calculated through the GSVA package.

### 2.6. Cell Culture and Establishment of Resistant Cell Line

Bladder cancer (BIU-87, 5637, T24, and RT112) cells were purchased from the American Type Culture Collection (ATCC, Manassas, VA, USA). The cells were grown in RPMI-1640 (Gibco, Waltham, MA, USA) with 10% fetal bovine serum (FBS, Gibco) in an atmosphere of 5% carbon dioxide at 37 °C. To acquire gemcitabine-resistant T24 cells and 5637 cells, the cells were exposed to a medium supplemented with GEM at an initial concentration of 0.1 μM in RPMI-1640 plus 10% FBS and incubated for 24 h. Then, the residual viable cells were expanded over 5 days in RPMI-1640 plus 10% FBS. Second and third rounds of selection were performed in a similar manner with increasing concentrations of GEM (0.5 μM, 1.0 μM, 2.0 μM, 4.0 μM, 8.0 μM, and 20 μM). Finally, we found that the resistant T24 cells could be stably cultured in RPMI-1640 plus 10% FBS with GEM at 10 μM.

A Cell Counting Kit-8 (CCK-8) assay was performed for the gemcitabine sensitivity assay. To determine the IC50, bladder cancer cells were cultured in 96-well plates containing fresh RPMI-1640 medium with approximately 5 × 10^3^ cells per well. After administering the corresponding concentration of the drug to each well, the culture was left for 48 h. Then, the absorbance was accurately measured at 450 nm using a microplate reader after incubating for another 2 h at 37 °C.

### 2.7. Glycolytic Activity Assay

We treated the cells with 2-NBDG for 1 h and then conducted a fluorescence-activated cell sorting analysis using a flow cytometer to quantify the glucose uptake capacity of the cells based on the fluorescence intensity. The cells were seeded in a 6-well plate and incubated for 24 h at 37 °C. Then, a lactic acid detection kit was used to determine the concentration of lactic acid in the culture medium. The production of lactic acid is expressed as the concentration of lactic acid per 1 × 10^4^ cells. At the same time, the pH of the culture medium was measured using a pH meter.

### 2.8. Quantitative Real-Time Polymerase Chain Reaction (qRT-PCR)

Total RNA was extracted from cells and tissues according to the manufacturer’s instructions. An ultraviolet spectrophotometer was used to measure the concentration and purity of RNA at 260/280 nm. We used a Primer-Script One-Step RT-PCR Kit (TaKaRa) to reverse transcribe cDNA from RNA. The real time PCR analyses were performed using SYBR on a 7500 Real Time PCR System (Applied Biosystems, CA, USA). The relative expression was normalized to the expression of GAPDH using the 2^−ΔΔCt^ method. The primer sequences were synthesized by Biotech (Shanghai, China) and are listed in Table S2. All experiments were performed in triplicate.

### 2.9. Western Blot Assay and Co-Immunoprecipitation

Western blot analyses were performed as previously described [8]. Briefly, protein samples lysed from bladder cancer cells or tissues were denatured, electrophoretically separated in 10% polyacrylamide gels, and transferred to poly (vinylidene difluoride) membranes (Millipore, Burlington, MA, USA). The blots were then probed with primary antibodies against NDUFA4L2 (1:200, Abcam, Cambridge, UK), NXPH4 (1:1000, Abmart, Berkeley Heights, NJ, USA), and GAPDH (1:1000, Cell Signaling Technology, Danvers, MA, USA). The grayscale of the indicated protein was measured with the software Image J (NIH Image, Maryland, USA). For co-immunoprecipitation, the collected cells were mixed by pipetting with a pre-chilled native lysate NETN (20 mM pH8.0 Tris HCl, 100 mM NaCl, 1 mM EDTA, 0.5% Nonidet P-40) and supplemented with protease inhibitors. The supernatant was incubated with antibodies overnight at 4 °C, followed by protein A/G plus agarose beads for 4 h at 4 °C. The immunoprecipitates were collected by centrifuging the samples and washing 5 times with lysis buffer. Western blotting is usually performed in the order of Co-IP group, Input1, Marker, IP group, and Input2.

### 2.10. Wound Healing and Transwell Invasion Assays

For the wound-healing assay, bladder cancer cells were seeded in 12-well plates until 80–90% confluence and then scratched by micropipette tip and cultured with serum-free medium for 48 h. Cell migration images were acquired at 0, 24, and 48 h after scratching. All experiments were conducted in triplicate.

To measure the invasion ability of bladder cancer cells, transwell invasion assays were performed using chambers (8 mm, BD Biosciences) coated with Matrigel (BD Biosciences, Shanghai, China). A total of 200 ul of bladder cancer cells resuspended with serum-free RPMI-1640 was loaded into the top of the chamber, while the lower chamber was loaded with 700 μL medium containing 30% FBS. After 24 h, the invading bladder cells on the bottom of the membrane were fixed and stained with violet crystalline. Finally, the invading cells in the lower chamber from five random fields were accurately counted via the microscopic assessment of five fields at a magnification of ×40.

### 2.11. CCK-8 Assay

Transfected cell suspensions were seeded on 96-well plates. We placed plates in incubators for 24, 48, 72, or 96 h (37 °C, 5% CO_2_). We added 10 μL of CCK solution to each well. After 2 h of incubation, a microplate analyzer was used to read the absorbance of each well at 450 nm.

### 2.12. Plate Clone Formation Assay

Stable transfected bladder cancer cells were cultured for two weeks on 6-well plates (0.5 × 10^3^/well). Cell clumps were washed with PBS and cells with 4% polyformaldehyde were fixed for 25–30 min. This was followed by 1% violet crystalline for 20–30 min after discarding the solution. Finally, a microscope was used to observe the number of colonies in each well.

### 2.13. Transfection

siRNAs targeting NXPH4 and NDUFA4L2, as well as siRNA negative controls, were provided by RiboBio (Guangzhou, China). The NDUFA4L2 overexpression vector (pcDNA-NDUFA4L2) and empty vector came from GeneChem (Shanghai, China), 1.6 mg of which were transfected to 12-well plates. The NXPH4 overexpression vector and empty vector came from WZ Biosciences (Shandong, China). Lipofectamine 2000 (Invitrogen, Carlsbad, CA, USA) and Opti-MEM (Gibco) were used for cell transfection according to the manufacturers’ instructions. Protein and total RNA were extracted 48–96 h after transfection for further assays. The siRNA sequences are shown in Table S3.

### 2.14. Immunohistochemical Staining

Immunohistochemical staining (IHC) was performed as previously described [9]. Briefly, human bladder tissue specimens were fixed in formalin and then embedded in paraffin and sliced. Sections were deparaffinized, blocked with goat serum, and then incubated with anti-NXPH4 (1:150) or anti-NDUFA4L2 (1:100) overnight at 4 °C. After 30 min incubation with horseradish-peroxidase-conjugated secondary antibody, the slices were counterstained with hematoxylin.

### 2.15. Intracellular ROS Levels

The intracellular ROS content of each group was determined using a reactive oxygen species detection kit (Byotime) according to the manufacturer’s protocol. Fluorescence microscopy was used to detect the DCF fluorescence distribution of cells. Green fluorescent spots indicated positive cells.

### 2.16. Cell Proliferation

A Cell-Light EdU DNA Cell Proliferation Kit (Beyotime, Shanghai, China) was used to test cell proliferation according to the manufacturer’s protocol. Briefly, cells in the logarithmic growth phase were seeded in 96-well plates, followed by cell fixation, EdU labeling, Apollo staining, and DNA staining. Finally, an Olympus microscope (Olympus, Tokyo, Japan) was used to acquire images.

### 2.17. Xenograft Tumor Model

BALB/c male nude mice (HFK Bio-Technology Co., Ltd., Beijing, China) were kept under specific pathogen-free conditions. Transfected T24 cells (5 × 10^6^) were suspended in 100 μL serum-free medium and subcutaneously injected into the back of 6-week-old BALB/c male nude mice. Tumor volumes were measured every 3 days using the formula V = 0.5 × L (length) × W2 (width). The mice were euthanized 36 days after the injection of the cells. Solid tumor tissues were obtained and the weight was measured. The Animal Research Committee of the Academic Medical Center at Huazhong University of Science and Technology approved all animal experiments. The Institutional and Animal Care and Use Committee guidelines were obeyed to ensure ethical animal treatment (Permission No: 655).

### 2.18. Statistical Analyses

All data are given as the mean ± standard deviation (SD) and were generated from three independent experiments. Student’s *t*-tests or χ2 tests using SPSS version 19.0 were utilized to analyze the results (SPSS Inc., Chicago, IL, USA) and GraphPad Prism 8 (GraphPad Software Inc., La Jolla, CA, USA) was used to present the results. *p* < 0.05 was considered to be statistically significant.

## 3. Results

### 3.1. The Expression Patterns of NXPH4 across 33 Kinds of Cancers from TCGA and Its Expression in Patients with Bladder Cancer from Different Groups

**Figure 1:**
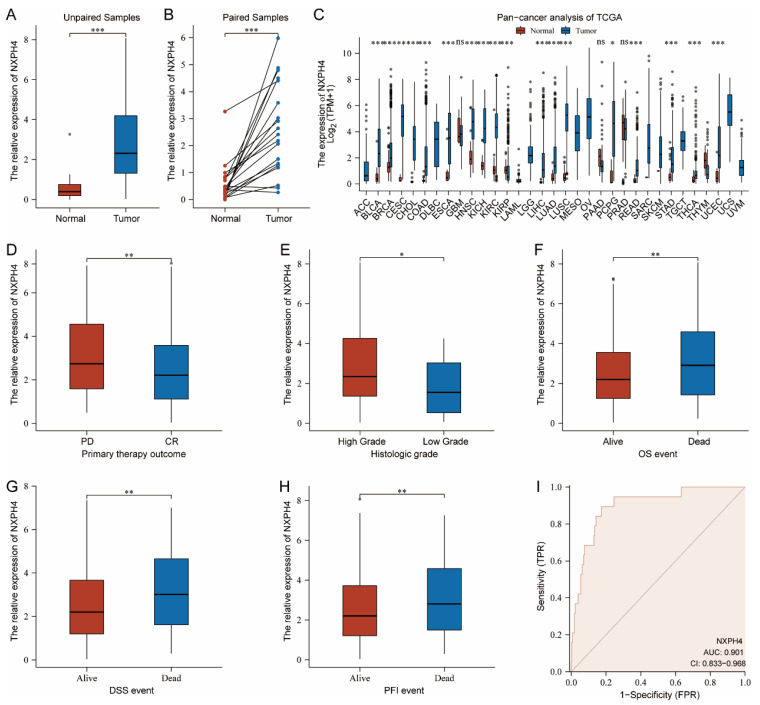
Bioinformatic analysis reveals the abnormal expression of NXPH4. (**A**–**C**) The distinct expression levels of NXPH4 in various kinds of cancers. (**D**) In contrast to patients with complete response, patients with progressive disease had higher levels of NXPH4. (**E**) The patients with bladder cancer of higher grade also experience higher expression of NXPH4 (**F**–**H**). Patients experiencing OS, DSS, and PFI have higher expression of NXPH4. (**I**) The receiver operating characteristics of NXPH4 in bladder cancer tissues and normal bladder tissues, * *p* < 0.05; ** *p* < 0.01; *** *p* < 0.001.

As Figure 1A suggests, we found that the transcriptome level of NXPH4 was elevated in bladder cancer tissues in contrast to normal bladder tissues. This was also observed in the paired cancer and normal tissues (Figure 1B). Consequently, we researched the expression levels of NXPH4 in other kinds of cancers and discovered that NXPH4 was raised in cervical squamous cell carcinoma, endocervical adenocarcinoma, cholangiocarcinoma, breast invasive carcinoma, colon adenocarcinoma, esophageal carcinoma, head and neck squamous cell carcinoma, kidney chromophobe, kidney renal clear cell carcinoma, glioblastoma multiforme, lung adenocarcinoma, and liver hepatocellular carcinoma, while in some kinds of cancers it was decreased or there was no significant difference between the normal and cancer tissues (Figure 1C). This suggests that NXPH4 may be an oncogene in some kinds of tumors while it may prevent the occurrence of tumor cells in other kinds of cancers. Moreover, we observed that among the patients with bladder cancer who received formal therapy, the patients experiencing progressive disease had higher levels of NXPH4 than patients experiencing complete responses (Figure 1D). In addition, it was found that compared with patients with lower grades of bladder cancer, patients with higher grades of bladder cancer had higher levels of NXPH4 (Figure 1E). When it came to the correlation between NXPH4 and survival outcomes, we found that in the fields of overall survival events, disease-specific events, and progress-free interval events, patients who had died all had higher levels of NXPH4 (Figure 1F–H). This indicated that NXPH4 may exert an influence on the prognosis of patients with bladder cancer. Finally, we created a receiver operating characteristic curve to judge whether NXPH4 could be used as a biomarker to distinguish between the normal bladder and cancer tissues. The area under the curve was 0.902, showing that this molecule may be a potential biomarker.

### 3.2. High Level of NXPH4 Is Associated with Worse Prognosis in Bladder Cancer

**Figure 2:**
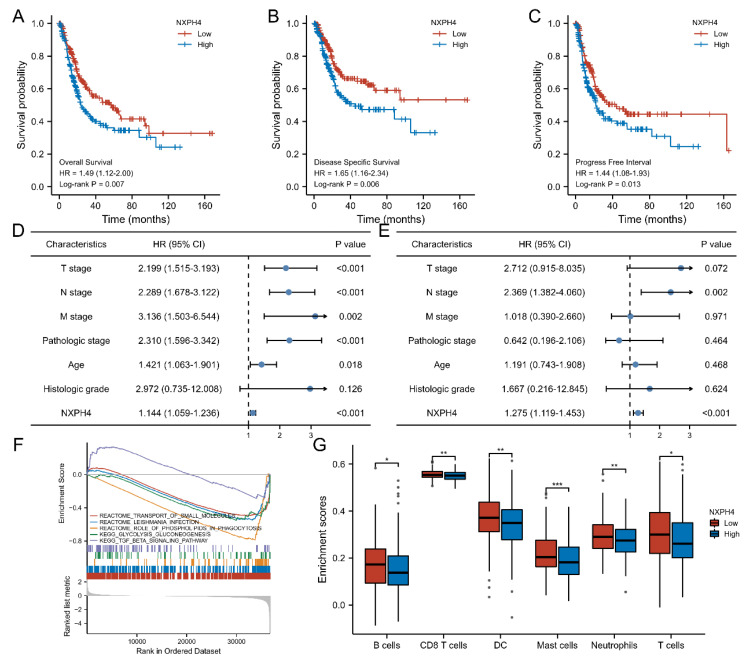
The prognostic value of NXPH4 and its possible mechanism. (**A**–**C**) Kaplan–Meier analysis for patients with bladder cancer in the fields of overall survival, disease-specific survival, and progression-free interval. (**D**,**E**) Univariate and multivariate analysis of the influence of some indicators on the overall survival of patients with bladder cancer. (**F**) Gene-set enrichment analysis after dividing groups by NXPH4 expression. (**G**) The relative levels of B cells, CD8 T cells, dendric cells, mast cells, neutrophils, and T cells between patients from the high-NXPH4 group and low-NXPH4 group, * *p* < 0.05; ** *p* < 0.01; *** *p* < 0.001.

According to the results above, we can speculate that the expression level of NXPH4 may be closely related to the survival outcomes of patients with bladder cancer. According to the results above, we can speculate that higher expression levels of NXPH4 may be closely related to worse survival outcomes (overall survival, disease-specific survival, progress-free interval) for patients with bladder cancer. The Kaplan–Meier analysis showed that even in different groups of patients with bladder cancer the patients with lower levels of NXPH4 had better prognoses than patients with higher levels. In the outcomes of overall survival, disease-specific survival, and progress-free interval, we observed that the patients with higher levels of NXPH4 experience have worse prognoses (Figure 2A–C). Owing to the fact that the Kaplan–Meier analysis is a method that only considers one variation, we conducted univariate and multivariate Cox regression analyses. The results showed that the expression of NXPH4 and N stage were independent indicators for the survival outcomes of patients with bladder cancer (Figure 2D,E). The gene set enrichment analysis showed that the expression level of NXPH4 was associated with glycolysis, which led us to conduct subsequent experiments (Figure 2F). Additionally, we found different infiltration levels of B cells, CD8 T cells, dendritic cells, mast cells, neutrophils, and T cells between the NXPH4-High and NXPH4-Low groups (Figure 2G).

### 3.3. NXPH4 Was Upregulated in BC Tissues and Cells, Promoting the Proliferation, Invasion, and Migration of BC Cells in Vitro

**Figure 3:**
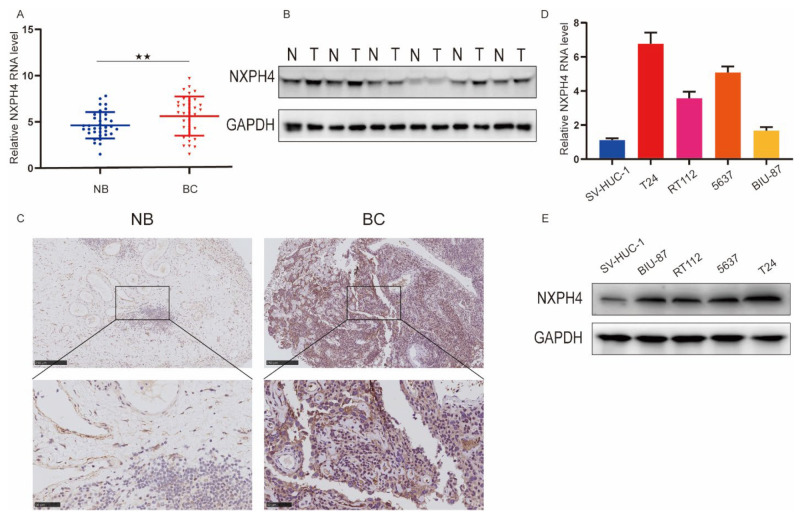
NXPH4 expression is elevated in BC tissues and cell lines. (**A**) NXPH4 expression was assessed by qRT-PCR in 34 BC and paired NB tissues. The horizontal line indicates the median value. (**B**) NXPH4 expression was assessed by Western blot in BC and paired NB tissues. (**C**) IHC staining of NXPH4 was performed in the tumor sections of NB and BC patients. (**D**,**E**) Both qRT-PCR and Western blot analyses of NXPH4 expression in bladder cancer cell lines (T24, 5637, RT112, and BIU-87) compared with a SV-HUC-1 normal human bladder cell line. * *p* < 0.05; ** *p* < 0.01; *** *p* < 0.001, the original western blot figures were showed in Figure S6.

To verify the expression levels of NXPH4 in bladder cancer (BC) tissues, qRT-PCR (reverse transcription-quantitative polymerase chain reaction) and WB (Western blotting) were performed. It was shown that the expression of NXPH4 was obviously elevated in tumor tissues in comparison with their para-cancer counterparts, normal bladder (NB) tissues (Figure 3A,B). The immunohistochemistry (IHC) results of cancer or corresponding adjacent normal tissues also suggested that NXPH4 was upregulated in bladder cancer tissues (Figure 3C). Moreover, we confirmed that the mRNA and protein levels of NXPH4 were higher in bladder cancer cell lines (BIU-87, 5637, RT112, and T24) than in the normal bladder cell line SV-HUC-1 by qRT-PCR and WB (Figure 3D,E). Generally speaking, these results indicate that NXPH4 is overexpressed in bladder cancer tissues.

**Figure 4:**
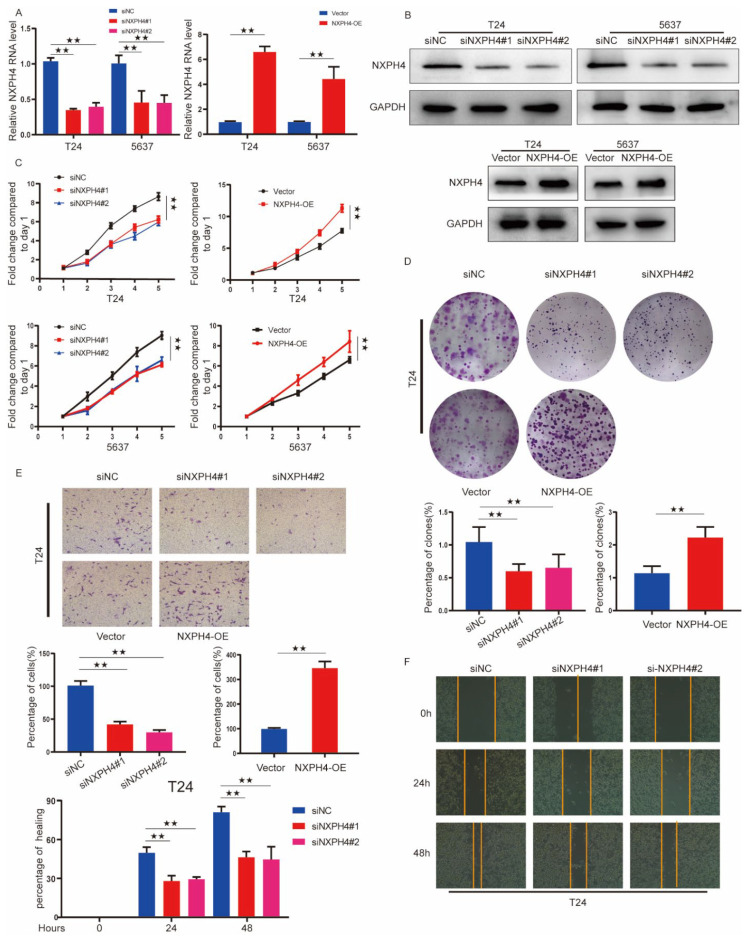
Knockdown or overexpression of NXPH4 altered BC cell invasion and proliferation. (**A**) NXPH4 knockdown or overexpression efficiency in T24 and 5637 cells was analyzed by qRT-PCR. (**B**) NXPH4 knockdown or overexpression efficiency in T24 and 5637 cells was analyzed by Western blot. (**C**,**D**) The effect of NXPH4 knockdown or overexpression on T24 and 5637 cell proliferation was measured via CCK-8 and colony formation assay. Five microscopic fields were chosen at random and averaged. (**E**,**F**) The effect of NXPH4 knockdown or overexpression on invasion and migration was analyzed using Transwell and wound healing assays in T24 cells, respectively. The lower histogram represents relative cell number while the representative images are shown on the top panel. Five microscopic fields were chosen at random and averaged. See Figure S4 also. * *p* < 0.05; ** *p* < 0.01; *** *p* < 0.001, the original western blot figures were showed in Figure S6.

To explore the effect of NXPH4 on the biological behaviors of bladder cancer, bladder cancer cell lines were transfected with si-NXPH4 or NXPH4 plasmid to down- or up-regulate the expression of NXPH4. The mRNA and protein expression levels markedly decreased or increased in T24 and 5637 cells compared with the corresponding negative controls (Figure 4A,B). The CCK-8 assays suggested that the downregulation and upregulation of NXPH4 in tumorous cells inhibited and promoted cell proliferation, respectively (Figure 4C). The colony formation assays also confirmed this result (Figure 4D and Figure S1A). Furthermore, the transwell assays and wound healing assays indicated that the level of NXPH4 was positively correlated with the invasion and migration abilities of the cells, respectively (Figure 4E,F and Figure S1B,C). The above results indicate that NXPH4 promotes the proliferation, migration, and invasion of bladder cancer cells, which is significant in the tumor metastasis cascade.

### 3.4. The Expression of NXPH4 Is Elevated in Acquired Gemcitabine-Resistant Bladder Cancer Cell Lines and Mediates Gemcitabine Resistance through Enhancing Both Intracellular Reactive Oxygen Species and Glycolysis

**Figure 5:**
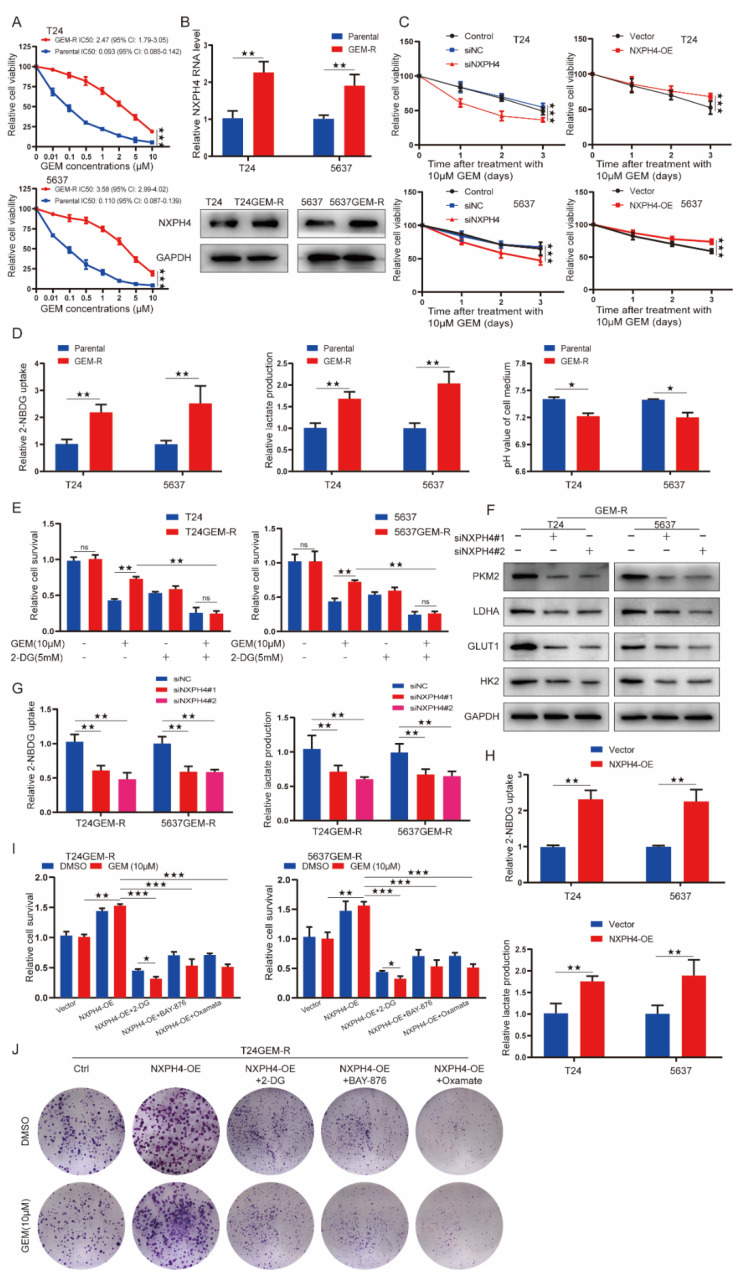
The expression of NXPH4 is elevated in acquired gemcitabine-resistant bladder cancer cell lines and mediates gemcitabine resistance through enhancing glycolysis. (**A**) Gemcitabine sensitivity assay showed a higher 50% maximal inhibitory concentration (IC50) value of gemcitabine in T24GEM-R and 5637GEM-R cells compared to parental cells. (**B**) NXPH4 expression was assessed by qRT-PCR and Western blot in T24 and 5637 cells treated with or without GEM (10 μM), followed by cell viability assay. (**C**,**D**) The influence of overexpressed and knockdown of NXPH4 on the cell survival when treated with gemcitabine and relative glycolysis levels in parental and GEM-R BC cells were assessed by assays of glucose uptake (left), lactate production (middle), and medium acidification (right). (**E**) Cells were cultured in normal or with 2-DG (5 mM) treatment conditions, with or without GEM (10 μM), followed by cell viability assays. (**F**–**I**) Cells were transfected with siNC, siNXPH4, empty vector, or NXPH4 plasmid, followed by glucose uptake assays (left) and lactate production assays (right). (**J**) Cells were transfected with empty vector plasmid or NXPH4 overexpression plasmid and cultured with or without 2-DG (5 mM), BAY-876 (50 nM), or oxamate (20 mM) treatment, followed by cell viability assays and colony formation assays. Values are significant at * *p* < 0.05, ** *p* < 0.01, and *** *p* < 0.001 as indicated. N.S. means the difference is not significant. The original western blot figures were showed in Figure S6.

In view of the fact that among the specimens we collected, 15 patients experienced relapses even after using gemcitabine, we examined the expression level of NXPH4 between the relapse tissue and the initial tumor tissue and found that NXPH4 was significantly elevated at the level of mRNA and protein when the patients experienced relapses. Thus, to better mimic the resistance of bladder cancer to gemcitabine chemotherapy, we induced gemcitabine-resistant bladder cancer cell lines, named T24GEM-R and 5637GEM-R, which are resistant to gemcitabine, by alternating exposure of T24 cells and 5637 cells to different concentrations of gemcitabine. Both T24GEM-R and 5637GEM-R cells showed significantly increased resistance to GEM compared to parental cells (Figure 5A).

To identify whether NXPH4 affects the drug sensitivity of bladder cancer cell lines, we compared the expression of NXPH4 between parental T24/5637 and T24GEM-R/5637GEM-R cells with qPCR and WB. Notably, the expression of NXPH4 was significantly higher in T24GEM-R and 5637GEM-R cells than that in parental cells (Figure 5B), which was consistent with the elevated NXPH4 expression in bladder cancer tissues of patients resistant to chemotherapy with gemcitabine. To further investigate the biological role of NXPH4 in gemcitabine-resistant bladder cancer cell lines, we knocked down the expression of NXPH4 in T24GEM-R and 5637GEM-R cell lines and found that it reversed the chemoresistance of T24GEM-R and 5637GEM-R cells as shown by GEM-R cell survival (Figure 5C). Conversely, the overexpression of NXPH4 enhanced T24 and 5637 cells’ viability (Figure 5C). Taken together, these results imply that NXPH4 is highly expressed in GEM-R cells and promotes gemcitabine resistance in GEM-R cells.

Recent studies have focused on the function of tumor metabolism, especially glycolysis, which is reported to play a role in promoting tumor drug resistance [10,11,12]. GEM-R bladder cancer cells showed increased glucose uptake, lactate production, and cell medium acidification (Figure 5D). Nevertheless, after treatment with 2-DG (inhibitor of glycolysis), GEM-R cells failed to resist the toxicity of gemcitabine, which demonstrated that GEM-R cells have an increased dependence on glycolysis (Figure 5E). Interestingly, the knockdown of NXPH4 significantly inhibited, while the overexpression of NXPH4 promoted, the glycolysis level of GEM-R bladder cancer cell lines (Figure 5F,G). Meanwhile, the cell viability of GEM-R bladder cancer cells induced by NXPH4 overexpression could be inhibited by 2-DG, BAY-876 (inhibitor for GLUT1), or oxamate (inhibitor of LDHA) (Figure 5H,I and Figure S2A).

**Figure 6:**
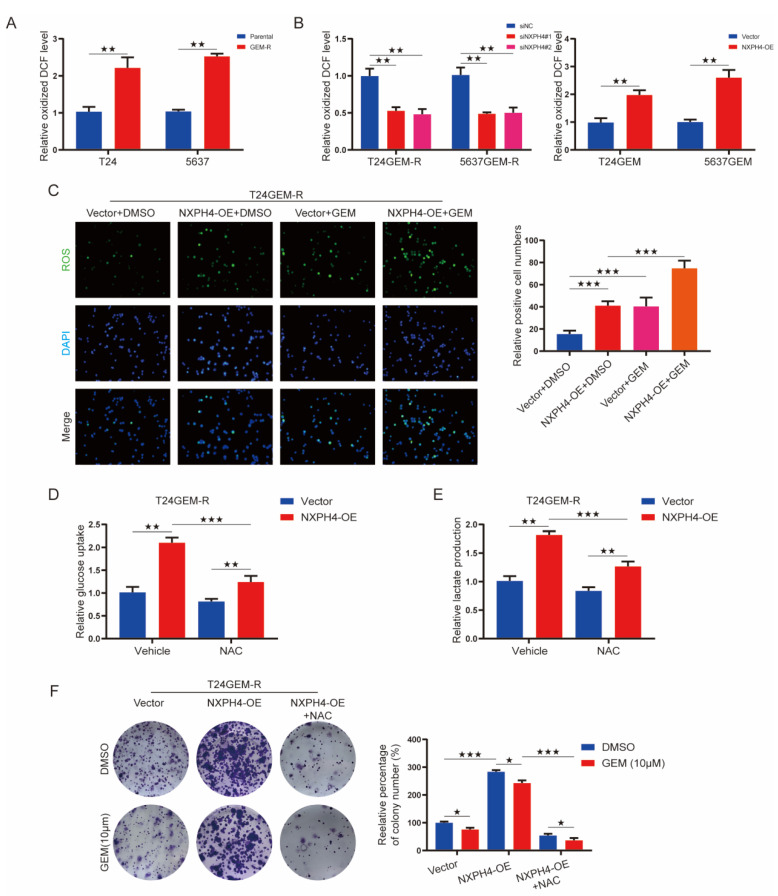
The expression of NXPH4 is elevated in acquired gemcitabine-resistant bladder cancer cell lines and mediates gemcitabine resistance through enhancing both intracellular reactive oxygen species and glycolysis. (**A**) The relative oxidized DCF (2’,7’-Dichlorofluorescein) levels between parental bladder cancer cell lines and gemcitabine-resistant cell lines. (**B**) The influence of knockdown and overexpression of NXPH4 on the relative oxidized DCF levels in parental or gemcitabine-resistant T24 and 5637 cell lines. (**C**) Overexpression of NXPH4 and the stimulation of gemcitabine increased the level of reactive oxygen species in gemcitabine-resistant T24 cell lines. (**D**,**E**) The use of the antioxidant N-acetylcysteine blocked the glycolysis level caused by the overexpression of NXPH4. (**F**) The use of the antioxidant N-acetylcysteine decreased the colony numbers caused by the overexpression of NXPH4. * *p* < 0.05; ** *p* < 0.01; *** *p* < 0.001. The original western blot figures were showed in Figure S6.

Reactive oxygen species (ROS) are the key mediators of many signaling cascades associated with cell proliferation and transformation and are closely connected to the occurrence of glycolysis. In fact, significantly higher levels of ROS were detected in resistant cells compared with parental cells with a reactive oxygen species kit (Figure 6A). We found that silencing of NXPH4 suppressed ROS production in T24GEM-R and 5637GEM-R cells, whereas NXPH4 overexpression increased ROS levels in T24 and 5637 cells (Figure 6B). Similarly, high expression of NXPH4 in T24GEM-R and 5637GEM-R cells also increased the level of ROS, and the level of ROS was also significantly increased under the stimulation of GEM (Figure 6C and Figure S3A). To test whether ROS are responsible for the enhanced tumorigenic properties of NXPH4 in bladder cancer, we blocked the production of ROS with the antioxidant N-acetylcysteine (NAC). In T24GEM-R and 5637GEM-R cells with overexpressed levels of NXPH4, NAC treatment greatly reduced the glycolytic phenotype (Figure 6D, E, and Figure S3B,C). In addition, NAC also reduced the colony number of NXPH4-expressed cells (Figure 6F and Figure S3D). Taken together, these results suggest that NXPH4 regulates intracellular ROS and promotes GEM resistance in bladder cancer cells in a glycolytic manner.

### 3.5. NDUFA4L2 Is a Downstream Target of NXPH4

**Figure 7:**
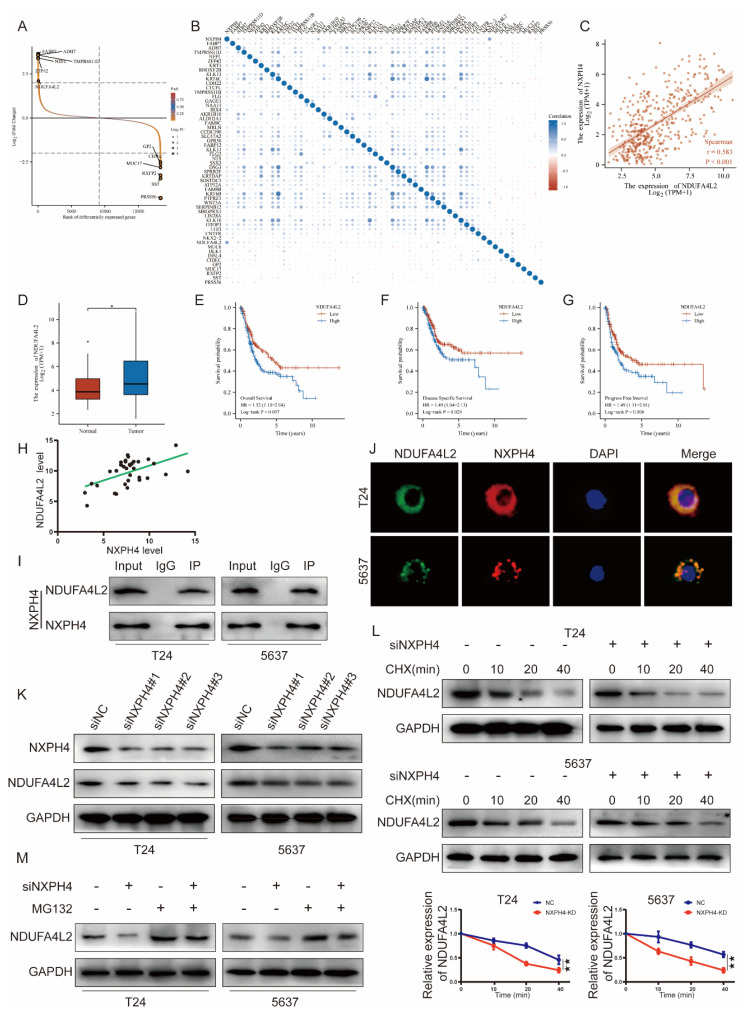
NDUFA4L2 is a downstream of NXPH4 in bladder cancer. (**A**) The top differentially expressed genes between low-NXPH4 and high-NXPH4 groups. (**B**) The Spearman’s correlation coefficient between the differentially expressed genes and NXPH4. (**C**) The Spearman’s correlation coefficient between NDUFA4L2 and NXPH4. (**D**) The expression of NDUFA4L2 is elevated in bladder cancer. (**E**–**G**) Higher expression of NDUFA4L2 predicts worse prognosis results in the fields of overall survival, disease-specific survival, and progress-free interval. (**H**) The correlation between the level of NXPPH4 and NDUFA4L2 in 34 human bladder cancer patient tissues. (**I**,**J**) Co-immunoprecipitation and immunofluorescence show that NXPH4 directly binds to NDUFA4L2. (**K**) The knockdown of NXPH4 also decreases the expression of NDUFA4L2 at the protein level. (**L**,**M**) NXPH4 influences the stability of NDUFA4L2 at the protein level. * *p* < 0.05; ** *p* < 0.01; *** *p* < 0.001. The original western blot figures were showed in Figure S6.

In order to deeply explore the mechanism of NXPH4 in bladder cancer, we searched for possible targets of NXPH4 by bioinformatic analysis. We screened out several possible downstream molecules and further validated their candidacy with experiments (Figure 7A). We calculated the expression correlation coefficient between the possible downstream molecules and NXPH4 with the RNA-seq data of patients from the TCGA-BLCA cohort. Among these molecules, the expression levels of NDUFA4L2, IRX4, WNT3A, LGALS7B, and OTOP3 are the most closely related to the expression of NXPH4. After the knockdown of the expression of NXPH4 in bladder cancer cell lines, we found that only the expression of NDUFA4L2 was influenced while others were not (Figure 4A). Additionally, we consistently found that patients with bladder cancer that had higher expression levels of NDUFA4L2 also experienced worse prognosis results (Figure 7D–G). In addition, NDUFA4L2 has also been reported to regulate the process of glycolysis. Moreover, by analyzing the clinical samples we collected we also found that NXPH4 was associated with NDUFA4L2, which was consistent with the TCGA results (Figure 7H). Therefore, we reasonably speculated that NDUFA4L2 was a downstream of NXPH4.

We then investigated whether NXPH4 could interact with NDUFA4L2. To this end, co-immunoprecipitation assays were conducted with lysates of cells transfected with HA-NXPH4 and GFP-mRFP-NDUFA4L2. Immunoprecipitation (IP) and immunofluorescence (IF) analysis indicated that NXPH4 directly bound and colocalized with NDUFA4L2 (Figure 7I,J). Moreover, NXPH4 knockdown significantly suppressed NDUFA4L2 expression at the protein level (Figure 7K). To assess the possible effect that NXPH4 exerts on the protein level of NDUFA4L2, NDUFA4L2 protein was measured in the presence of the proteasome inhibitor cycloheximide. The results showed that the stability of the NDUFA4L2 protein was decreased by NXPH4 knockdown in T24 and 5637 cells (Figure 7L). Therefore, we thought that NXPH4 might bind to NDUFA4L2 and protect it from degradation. Furthermore, the proteasome inhibitor MG132 could rescue NDUFA4L2 protein levels from inhibition by NXPH4 downregulation in T24 and 5637 cells (Figure 7M). All this indicates that NXPH4 might regulate NDUFA4L2 expression at the protein level.

### 3.6. NXPH4 Promotes ROS Production and Glycolysis-Dependent Gemcitabine Resistance by Regulating NDUFA4L2

**Figure 8:**
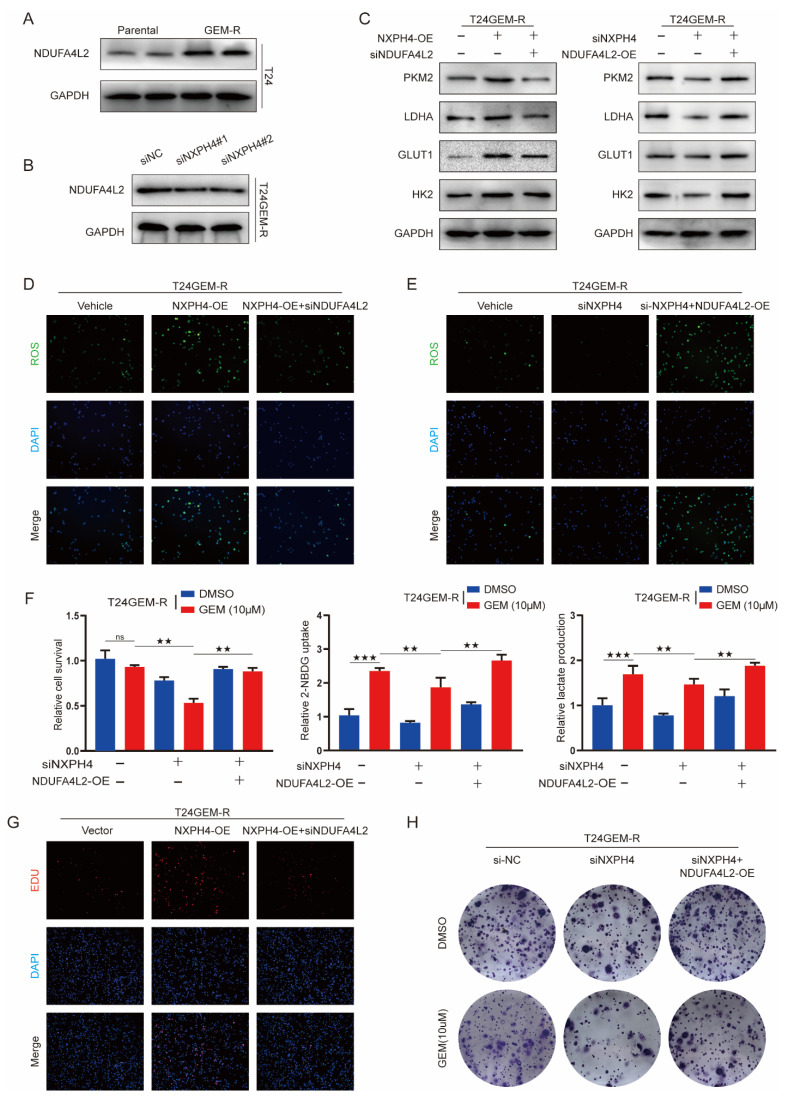
NXPH4 promotes ROS production and glycolysis-dependent gemcitabine resistance by regulating NDUFA4L2. (**A**) Western blot of NDUFA4L2 in T24 parental and gemcitabineresistant cell lines. (**B**,**C**) Cells were transfected with siNXPH4 or NXPH4 plasmid, followed by siNDUFA4L2 or NDUFA4L2 plasmid to detect the influence of NXPH4 on the crucial proteins of glycolysis. (**D**,**E**) Knockdown and overexpression of NDUFA4L2 reversed the effect of the overexpression and knockdown of NXPH4 on the level of reactive oxygen species. (**F**) Cells were transfected with siNC or siNXPH4, or co-transfected with NDUFA4L2 plasmid, followed by CCK8 assays, 2-NBDG uptake assays, and lactate production assays.(**G**,**H**) NDUFA4L2 reversed the proliferative effect caused by NXPH4 with EDU and colony assays in the gemcitabine-resistant T24 cell line. * *p* < 0.05; ** *p* < 0.01; *** *p* < 0.001. N.S. means the difference is not significant. The original western blot figures were showed in Figure S6.

Considering that NDUFA4L2 is associated with oxidative stress and glycolysis in various diseases, we speculated that NXPH4 promotes drug resistance in bladder cancer cells by regulating oxidative stress and glycolysis through NDUFA4L2. Indeed, we found that NDUFA4L2 expression was increased in drug-resistant cells and that knockdown of NXPH4 significantly suppressed NDUFA4L2 expression at the protein level (Figure 8A,B and Figure S5A,B). While the effect caused by knockdown of NXPH4 was different following the overexpression of NDUFA4L2, the oxidative stress, chemoresistance, and elevated level of glycolysis caused by the overexpression of NXPH4 were suppressed by NDUFA4L2 silencing (Figure 8C,D and Figure S5C,D). Consistently, the overexpression of NDUFA4L2 dampened the reduction in ROS, attenuated chemoresistance, and downregulation of glycolysis levels caused by NXPH4 silencing (Figure 8E,F and Figure S5E,F). The results of the EDU experiments indicate that the expression level of NDUFA4L2 can affect the proliferation level of T24 and 5637 cells (Figure 8G and Figure S4G). In addition, silencing NDUFA4L2 also reduces the colony numbers of cells with NXPH4 overexpression (Figure 8H and Figure S5H). Taken together, these results suggest that NDUFA4L2 is an important target for NXPH4 to function in GEM-R bladder cancer cells.

### 3.7. NXPH4-NDUFA4L2 Pathway Is Essential for Gemcitabine Resistance of Bladder Cancer Cells In Vivo

**Figure 9:**
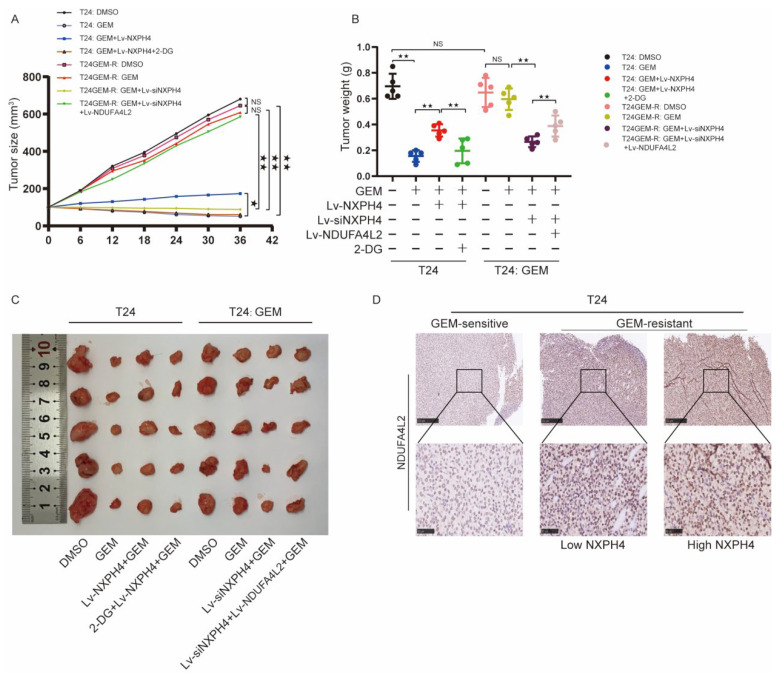
NXPH4/NDUFA4L2 pathway is essential for GEM resistance of BC cells in vivo. (**A**–**C**) Subcutaneous implantation mouse models were established using parental or GEM-R BC cells transfected with lentivirus containing NXPH4 plasmid and siNXPH4 and/or NDUFA4L2 plasmid. Growth curve (**A**), representative images (**B**), and tumor weight (**C**) of xenografts (*n* = 5) in the eight treatment groups for 36 days are shown. (**D**) IHC staining of NDUFA4L2 was performed in the tumor sections from mouse models that were established using parental or GEM-R BC cells transfected with lentivirus containing NXPH4 plasmid and siNXPH4. * *p* < 0.05; ** *p* < 0.01; *** *p* < 0.001. N.S. means the difference is not significant.

We performed xenograft mouse models to further reveal the effects of the NXPH4/NDUFA4L2 pathway on chemotherapy resistance in bladder cancer in vivo. Consistent with our previous in vitro results, the overexpression of NXPH4 significantly abolished the inhibitory effects of GEM treatment in parental bladder cancer cell-derived groups, as indicated by the increased tumor size and tumor weight (Figure 9A). Nevertheless, the treatment of 2-DG suppressed the GEM resistance induced by the overexpression of NDUFA4L2 (Figure 9B). Similarly, NXPH4 knockdown inhibited GEM resistance in GEM-R BC cell-derived xenografts, which could be partly reversed by the overexpression of NDUFA4L2 (Figure 9C). IHC assays of GEM-R BC cell-derived xenografts showed that the expression of NDUFA4L2 could be downregulated by NXPH4 knockdown (Figure 9D). Thus, the results above elucidated that NXPH4/NDUFA4L2 was a potential target for the treatment of GEM-resistant bladder cancer.

## 4. Discussion

Several articles have reported on the structure and function of NXPH4 in certain biological processes [7]. Xiangling Meng et al. [6] stressed the significant role of neurexophilin 4 in modulating specific cerebellar synapses and motor functions. It is believed to bind to α-neurexin and to have the function of regulating signal transduction in the brain [13]. However, no research has found that the expression level of this molecule is associated with the prognosis of patients with bladder cancer. With the advancements in bioinformatics technologies, we can gain easily overlooked but useful information from large-scale data more conveniently. Through an analysis of the transcriptome data of patients from TCGA with 33 kinds of cancers, we found the distinct expression patterns of NXPH4 across multiple cancers and specifically explored its clinical relevance in bladder cancer. We found that patients with high disease progress and high grades of bladder cancer had higher expression levels of NXPH4 in contrast to those patients with complete responses and lower grades of bladder cancer. Additionally, in this way, we first found the possible relationship between NXPH4 and the survival outcome of patients with bladder cancer and revealed the underlying mechanism with a series of experiments.

The possibility of proliferation, invasion, and migration have been proven to be significant factors for the survival and metastasis of tumor cells. Therefore, our research conducted a CCK-8 assay, wound scratch assay, and transwell invasion assay to judge whether NXPH4 influences these abilities of tumor cells. Additionally, we proved that NXPH4 is an oncogene through these experiments. In another field, resistance to chemotherapy exerts a great impact on the results of the prognosis of patients with bladder cancer [14,15]. Von der Maase and Hurst C have both stressed the significant role of reducing chemoresistance in the treatment of bladder cancer [16,17]. Gemcitabine is one of the most prevalent chemotherapy agents [18]. Additionally, after we induced the gemcitabine-resistant cell line, we examined the expression level of NXPH4 between parental and GEM-R cells and found that elevated NXPH4 promoted chemoresistance.

It has been widely reported that glycolysis plays a significant role in the chemotherapy resistance of tumor cells [19,20,21,22]. For example, Xiaoduan Li et al. proved that CTSLP8 mediates chemotherapy resistance by modulating cellular glycolysis in ovarian cancer [23]. The research of Mengxin Li also showed that RSL3 enhances the antitumor effect of cisplatin on prostate cancer cells by causing glycolysis dysfunction [24]. We conducted GSEA analyses and the result indicate that the expression of NXPH4 promotes the glycolysis of tumor cells. Consequently, we detected the glycolysis level in the gemcitabine-resistant T24 and 5637 cell lines. We found that in contrast to T24 and 5637, both T24GEM-R and 5637GEM-R have higher levels of 2-NBDG uptake and lactate production. Therefore, we speculated that NXPH4 promotes gemcitabine resistance through elevated glycolysis. Additionally, in the subsequent experiment, we found when we added 2-DG, which can inhibit glycolysis, the drug resistance of T24GEM-R and 5637GEM-R was reversed, which confirmed our suspicions. Glycolysis is crucial in the proliferation of cells [25,26,27]. Yanhen Zhou et al. have proven that rabeprazole can inhibit cell proliferation in gastric epithelial cells by targeting glycolysis [28]. Fan Wu et al. also demonstrated that the STK25-induced inhibition of aerobic glycolysis could suppress cell proliferation in colorectal cancer [29]. Based on the abovementioned results, we also examined the impact of glycolysis on the proliferation ability in our research and found that NXPH4 promotes proliferation through glycolysis. In conclusion, our results indicate that NXPH4 promotes proliferation and chemoresistance through glycolysis. However, the mechanism and the interactions between NXPH4 and other molecules are still unknown at this time.

ROS have been reported to influence the level of glycolysis in many studies [30,31,32,33,34]. Therefore, we examined the ROS levels between parental bladder cancer cells and gemcitabine-resistant cells and found higher levels of ROS in the drug-resistant cells. Moreover, to explore the impact that NXPH4 exerts on the level of ROS, both knockdown and overexpression of NXPH4 were examined. We found that lower expression of NXPH4 decreased the level of ROS while higher expression of NXPH4 showed the opposite. After using the ROS inhibitor NAC, the impact NXPH4 exerted on glycolysis was reversed. These results indicate that NXPH4 promotes glycolysis through elevated ROS levels.

NDUFA4L2 has been widely reported to induce glycolysis [35,36,37,38,39]. We found elevated protein levels of NDUFA4L2 in both T24GEM-R and 5637GEM-R. After the overexpression and knockdown of NXPH4 in T24GEM-R and 5637GEM-R, we found associated upregulation and downregulation of NDUFA4L2, respectively, indicating that NXPH4 may function through the regulation of NDUFA4L2. VHL, PKM2, GLUT1, and HK2 are crucial target genes for NDUFA4L2 [39] and are responsible for a series of biological processes such as cell survival and apoptosis [40]. In order to confirm our hypothesis, we detected the protein levels of these genes after NXPH4 knockdown. Fortunately, the results were consistent with our suspicions. To confirm that NDUFA4L2 could dampen the effect of NXPH4 on glycolysis and cell proliferation, we examined the cell survival, 2-NBDG uptake, and lactate production under different conditions. The results can be seen in Figure 8E–H and Figure S8E–H. We thus proved that NXPH4 mediates glycolysis and cell proliferation through the NDUFA4L2 signaling pathway.

In vitro experiments alone are not enough. Therefore, we consequently conducted experiments to further confirm the theory in vivo. We found that the expression of NXPH4 and NDUFA4L2 and the level of glycolysis have a great influence on the tumor size and tumor weight originating in mice, which is consistent with the theory proven in vitro.

## 5. Conclusions

In summary, our research found a new molecule, NXPH4, which influences the prognosis of patients with bladder cancer, and revealed that NXPH4 promotes progression, metastasis, and gemcitabine resistance in bladder cancer by enhancing glycolysis through modulating the expression of NDUFA4L2.

## Data Availability

The datasets generated and/or analyzed during the current study are available either in this article or in the Supplementary Materials.

## Supplementary Materials

The following are available online at https://www.mdpi.com/article/10.3390/cancers14153782/s1, NXPH4 Promotes Gemcitabine Resistance in Bladder Cancer by Enhancing Reactive Oxygen Species and Glycolysis Activation through Modulating NDUFA4L2. Figure S1: Knockdown or overexpression of NXPH4 altered the proliferation, invasion, and migration of 5637 bladder cancer cells. (A) The effect of NXPH4 knockdown or overexpression on cell proliferation in 5637 cell lines was measured via colony formation assay. Five microscopic fields were chosen at random and averaged. (B,C) The effect of NXPH4 knockdown or overexpression on invasion and migration was analyzed using Transwell and wound healing assays, respectively, in 5637 cell lines. Five microscopic fields were chosen at random and averaged; Figure S2: The glycolysis inhibitors reverse the proliferative and gemcitabine-resistant effect caused by the overexpression of NXPH4. (A) Cells were transfected with empty vector plasmid or NXPH4 overexpression plasmid and cultured with or without 2-DG (5 mM), BAY-876 (50 nM), or oxamate (20 mM) treatment, followed by colony formation assays; Figure S3: NXPH4 promotes glycolysis by enhancing the level of reactive oxygen species. (A) Overexpression of NXPH4 and the stimulation of gemcitabine increased the level of reactive oxygen species in gemcitabine-resistant 5637 cell lines. (B,C) The use of antioxidant N-acetylcysteine blocked the glycolysis level caused by the overexpression of NXPH4 in gemcitabine-resistant 5637 cell lines. (D) The use of antioxidant N-acetylcysteine decreased the colony numbers caused by the overexpression of NXPH4 in gemcitabine-resistant 5637 cell lines; Figure S4: NDUFA4L2 is a Downstream target of NXPH4. (A) Western blot of NDUFA4L2, IRX4, WNT3A, LGALS7B, and PTPRZ1 OTOP3 in T24 cell lines; Figure S5: NXPH4 promotes ROS production and glycolysis-dependent gemcitabine resistance by regulating NDUFA4L2. (A) Western blot of NDUFA4L2 in 5637 parental and gemcitabine-resistant cell lines. (B,C) Cells were transfected with siNXPH4 or NXPH4 plasmid, followed by siNDUFA4L2 or NDUFA4L2 plasmid to detect the influence of NXPH4 on the crucial proteins of glycolysis. (D,E) Knockdown and overexpression of NDUFA4L2 reversed the effect of the overexpression and knockdown of NXPH4 on the level of reactive oxygen species. (F) Cells were transfected with siNC or siNXPH4, or co-transfected with NDUFA4L2 plasmid, followed by CCK8 assays, 2-NBDG uptake assays, and lactate production assays. (G,H) NDUFA4L2 reversed the proliferative effect caused by NXPH4 with EDU and colony assays in the gemcitabine-resistant 5637 cell line. * *p* < 0.05; ** *p* < 0.01; *** *p* < 0.001. N.S. means the difference is not significant; Figure S6: the original western blot figures; Table S1: The baseline data of patients with bladder cancer from TCGA; Table S2: Primer Sequences used for qRT-PCR; Table S3: The sequence of siRNA.

## Abbreviations

BC	Bladder cancer
NB	Normal bladder
N	Normal
T	Tumor
TCGA	The Cancer Genome Atlas;
OS	Overall survival
DSS	Disease Specific Survival
PFI	Progression Free Interval
CR	Complete Response;
PR	Partial Response;
SD	Stable Disease;
PD	Progressive Disease;
GSEA	Gene Set Enrichment Analysis;
KM analysis	Kaplan-Meier analysis;
GEM	Gemcitabine;
T24-GEM-R	Gemcitabine resistant T24;
5637GEM-R	Gemcitabine resistant 5637
IC50	Half maximal inhibitory concentration.
ROS	Reactive oxygen species
