# Supplementary material for: NXPH4 Promotes Gemcitabine Resistance in Bladder Cancer by Enhancing Reactive Oxygen Species and Glycolysis Activation through Modulating NDUFA4L2
Source: Cancers (Basel). 2022 Aug 3;14(15):3782. doi: 10.3390/cancers14153782 (PMC9367313; doi:10.3390/cancers14153782)
Supplement: Supplementary file 1 [file cancers-14-03782-s001.zip › cancers-1776274-supp-final.pdf]

Supplementary Materials: NXPH4 Promotes Gemcitabine Resistance in Bladder Cancer by Enhancing Reactive Oxygen Species and Glycolysis Activation through Modulating NDUFA4L2

Decai Wang, Pu Zhang, Zijian Liu, Yifei Xing and Yajun Xiao

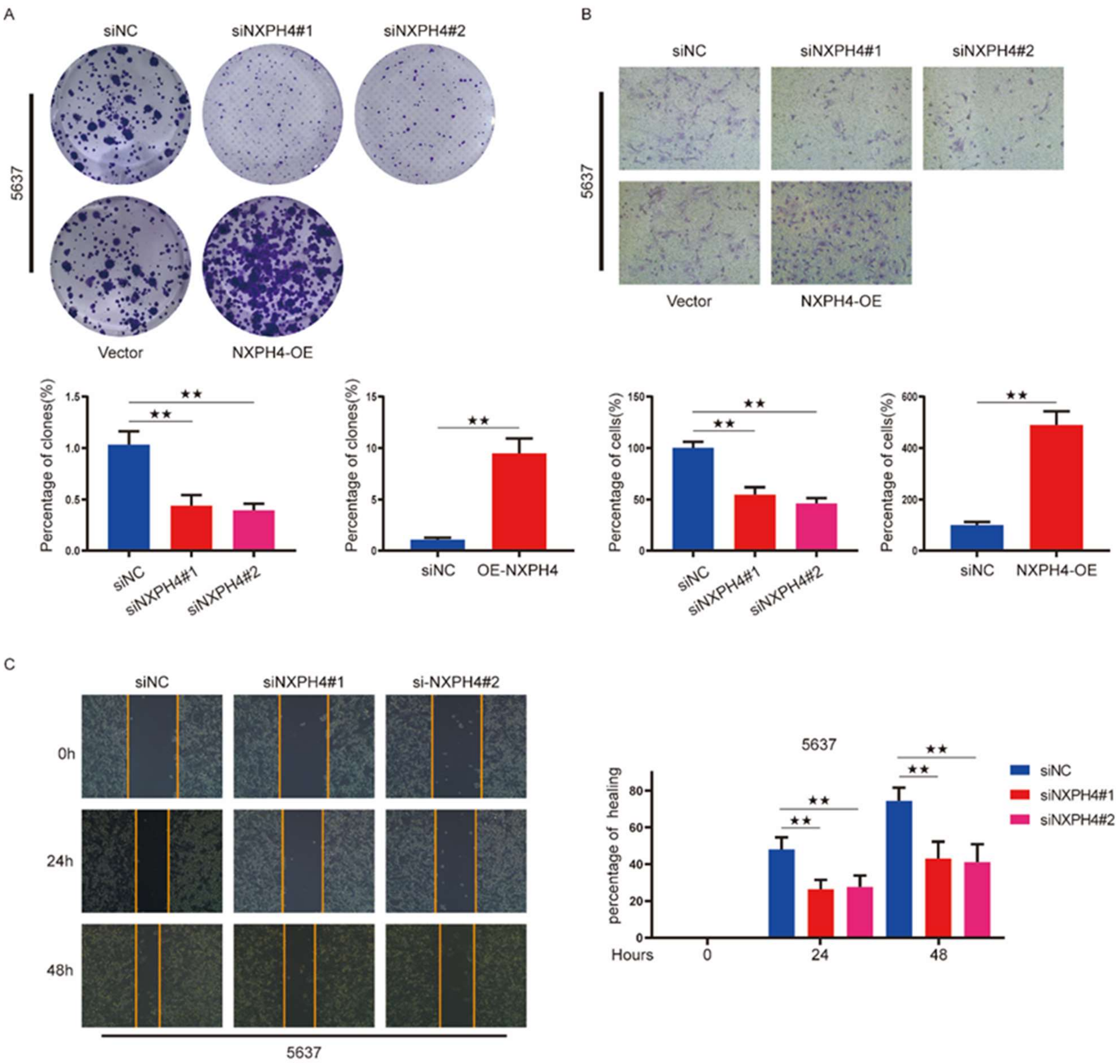

Figure S1. Knockdown or overexpression of NXPH4 altered the proliferation, invasion, and migration of 5637 bladder cancer cells. (A) The effect of NXPH4 knockdown or overexpression on cell proliferation in 5637 cell lines was measured via colony formation assay. Five microscopic fields were chosen at random and averaged. (B,C) The effect of NXPH4 knockdown or overexpression on invasion and migration was analyzed using Transwell and wound healing assays, respectively, in 5637 cell lines. Five microscopic fields were chosen at random and averaged.

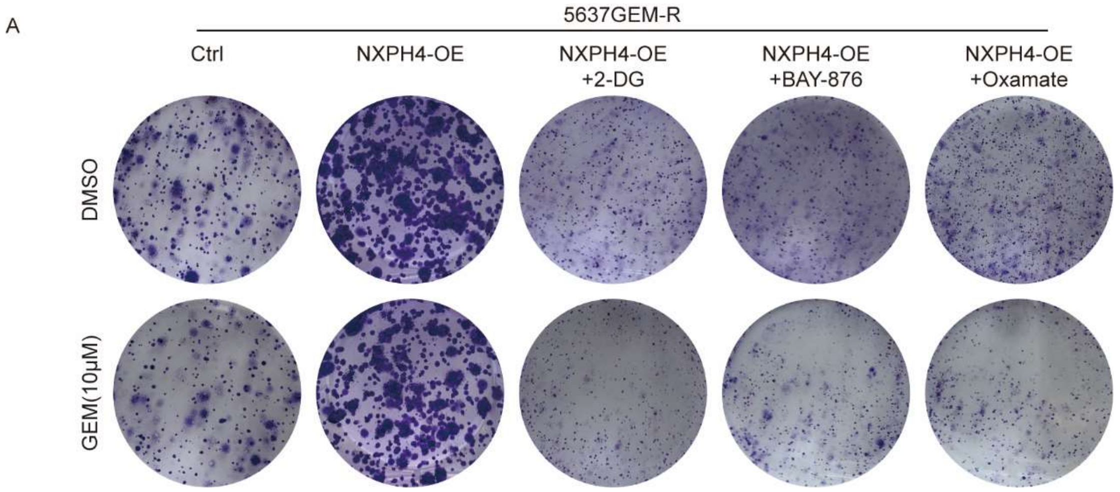

Figure S2. The glycolysis inhibitors reverse the proliferative and gemcitabine-resistant effect caused by the overexpression of NXPH4. (A) Cells were transfected with empty vector plasmid or NXPH4 overexpression plasmid and cultured with or without 2-DG (5 mM), BAY-876 (50 nM), or oxamate (20 mM) treatment, followed by colony formation assays.

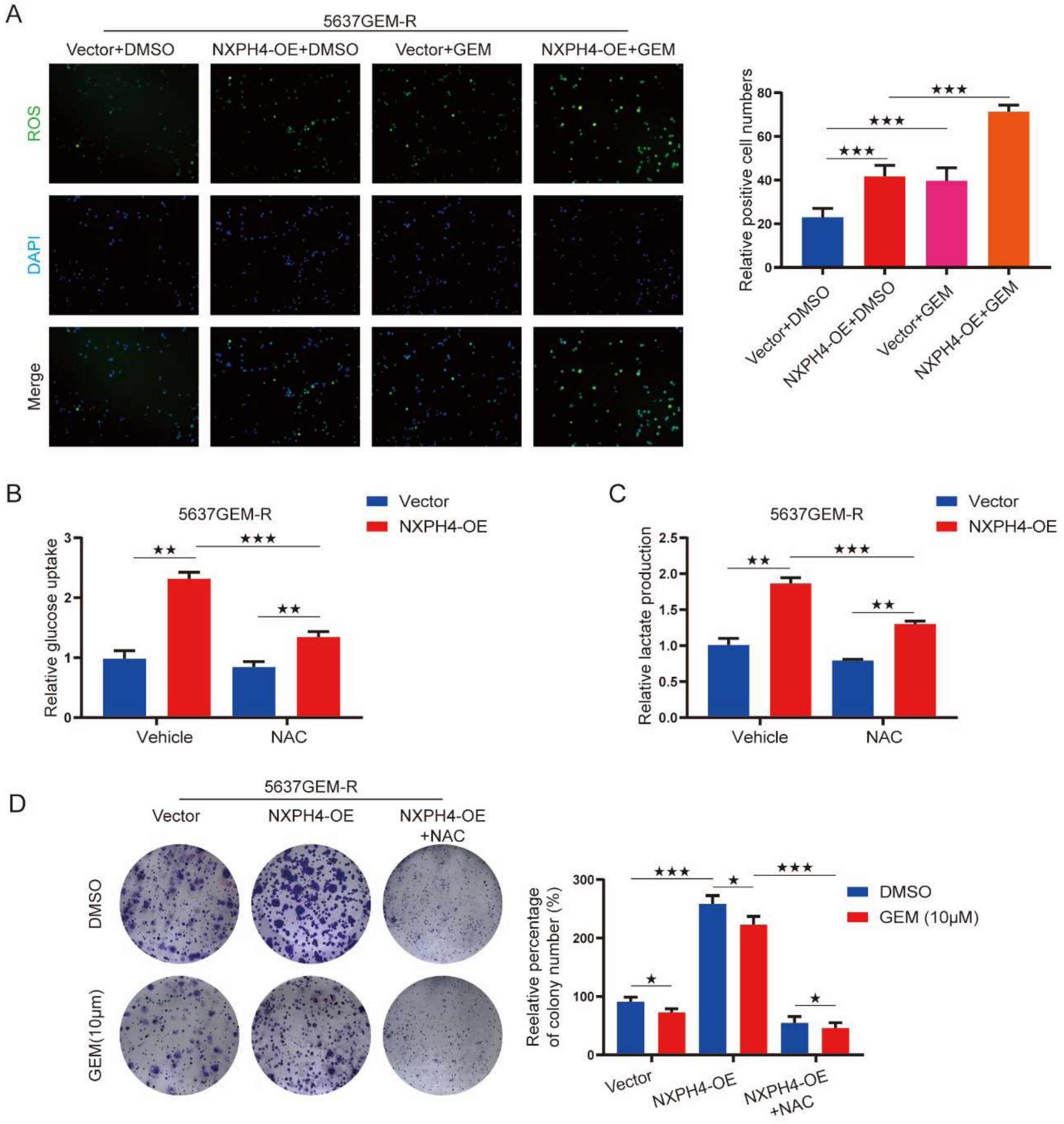

Figure S3. NXPH4 promotes glycolysis by enhancing the level of reactive oxygen species. (**A**) Overexpression of NXPH4 and the stimulation of gemcitabine increased the level of reactive oxygen species in gemcitabine-resistant 5637 cell lines. (**B,C**) The use of antioxidant N-acetylcysteine blocked the glycolysis level caused by the overexpression of NXPH4 in gemcitabine-resistant 5637 cell lines. (**D**) The use of antioxidant N-acetylcysteine decreased the colony numbers caused by the overexpression of NXPH4 in gemcitabine-resistant 5637 cell lines.

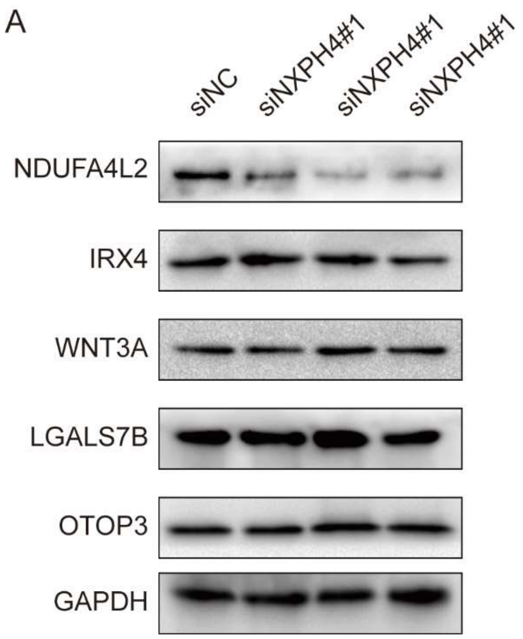

Figure S4. NDUFA4L2 is a Downstream target of NXPH4. (**A**) Western blot of NDUFA4L2, IRX4, WNT3A, LGALS7B, and PTPRZ1 OTOP3 in T24 cell lines.

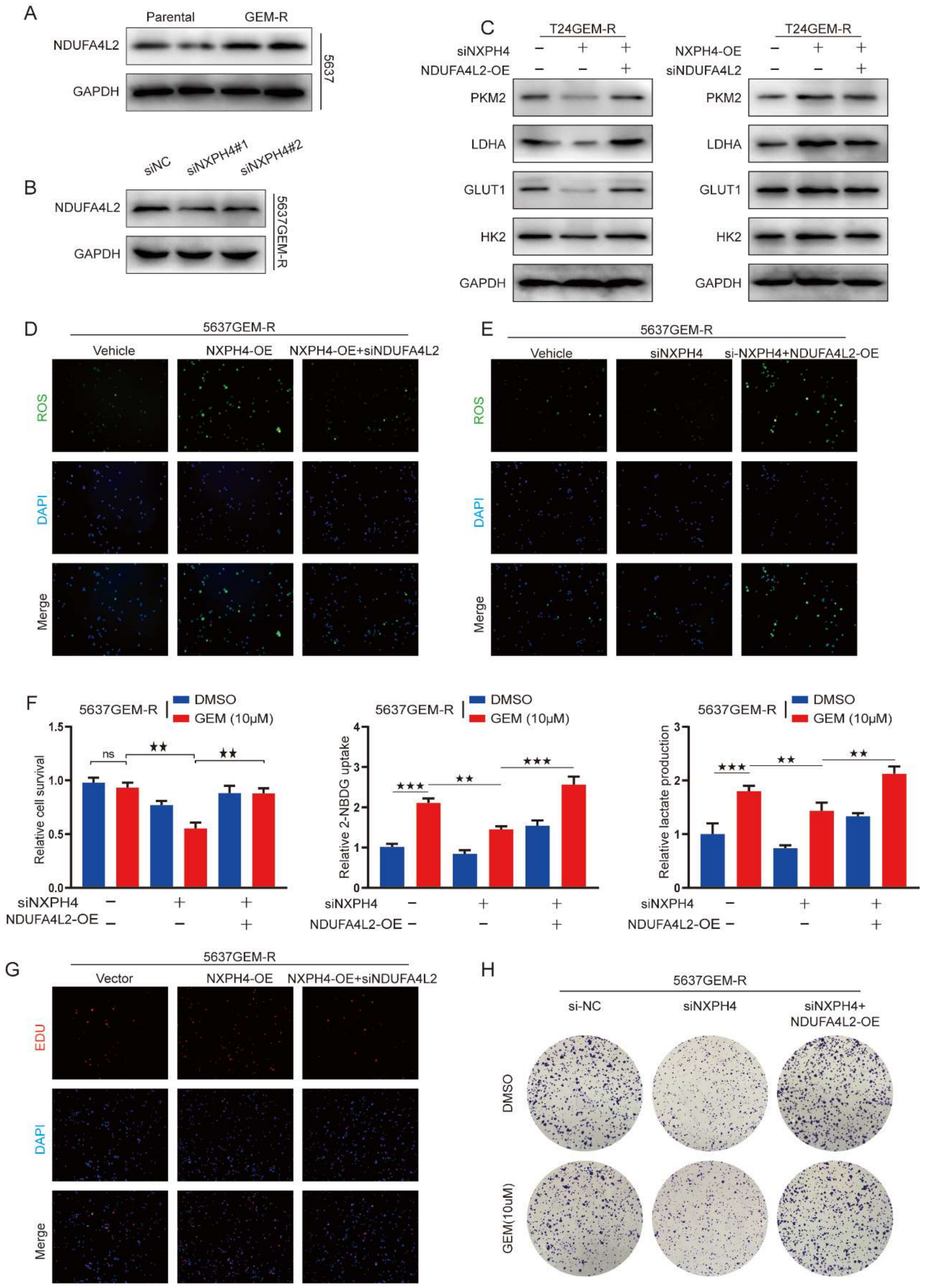

Figure S5. NXPH4 promotes ROS production and glycolysis-dependent gemcitabine resistance by regulating NDUFA4L2. (**A**) Western blot of NDUFA4L2 in 5637 parental and gemcitabine-resistant cell lines. (**B**,**C**) Cells were transfected with siNXPH4 or NXPH4 plasmid, followed by siNDUFA4L2 or NDUFA4L2 plasmid to detect the influence of NXPH4 on the crucial proteins of glycolysis. (**D**,**E**) Knockdown and overexpression of NDUFA4L2 reversed the effect of the overexpression and knockdown of NXPH4 on the level of reactive oxygen species. (**F**) Cells were transfected with siNC or siNXPH4, or co-transfected with NDUFA4L2 plasmid, followed by CCK8 assays, 2-NBDG uptake assays, and lactate production assays. (**G**,**H**) NDUFA4L2 reversed the proliferative effect caused by NXPH4 with EDU and colony assays in the gemcitabine-resistant 5637 cell line. * $p < 0.05$; ** $p < 0.01$; *** $p < 0.001$. N.S. means the difference is not significant.

Figure S6. All original western blot figures.

Table S1. The baseline data of patients with bladder cancer from TCGA.

Characteristic	levels	Overall
n		414
T stage, *n* (%)	T1	5 (1.3%)
	T2	119 (31.3%)
	T3	196 (51.6%)
	T4	60 (15.8%)
N stage, *n* (%)	N0	239 (64.6%)
	N1	46 (12.4%)
	N2	77 (20.8%)
	N3	8 (2.2%)
M stage, *n* (%)	M0	202 (94.8%)
	M1	11 (5.2%)
Pathologic stage, *n* (%)	Stage I	4 (1%)
	Stage II	130 (31.6%)
	Stage III	142 (34.5%)
	Stage IV	136 (33%)
Gender, *n* (%)	Female	109 (26.3%)
	Male	305 (73.7%)
Race, *n* (%)	Asian	44 (11.1%)
	Black or African American	23 (5.8%)
	White	330 (83.1%)
Age, *n* (%)	<=70	234 (56.5%)
	>70	180 (43.5%)
Histologic grade, *n* (%)	High Grade	390 (94.9%)
	Low Grade	21 (5.1%)
Subtype, *n* (%)	Non-Papillary	275 (67.2%)
	Papillary	134 (32.8%)
Age, median (IQR)		69 (60, 76)

Table S2. Primer Sequences used for qRT-PCR.

Primer	Sequence
NXPH4	Forward: 5'-GCAGCGAAAACTTGAGGGTAT-3′
	Reverse: 5'-AAGGTCTTCGGACGGCCTA-3′
NDUFA4L2	Forward: 5'-TCCATGTGACCATGAGGAAA-3′
	Reverse: 5'-CCAAGCAGGTCATAGGTGGT-3'
GAPDH	Forward: 5'-GAAGGTGAAGGTCGGAGTC-3'
	Reverse: 5'-GAAGATGGTGATGGGATT-3'

Table S3. The sequence of siRNA.

SiRNA Targets	SS Sequences	AS Sequences
NXPH4#1	GGACUUCUACUUUCGGGUG	CACCCGAAAGUAGAAGUCC
NXPH4#2	CACGCGCUUUCAAUUGCCA	UGGCAAUUGAAAGCGCGUG
NXPH4#3	CCAGACUAUAACUUCCAGA	UCUGGAAGUUAUAGUCUGG
NDUFA4L2#1	CCCGCUUCUACCGGCAGAUTT	AUCUGCCGGUAGAAGCGGGTT
NDUFA4L2#2	GCGCUGCGCUUUACUUGCUTT	AGCAAGUAAAGCGCAGCGCTT
NDUFA4L2#3	GCUGGGACAGAAAGAACAATT	UUGUUCUUUCUGUCCCAGCTT
