# Supplementary figures and images for: NXPH4 Promotes Gemcitabine Resistance in Bladder Cancer by Enhancing Reactive Oxygen Species and Glycolysis Activation through Modulating NDUFA4L2
Source: Cancers (Basel). 2022 Aug 3;14(15):3782. doi: 10.3390/cancers14153782 (PMC9367313; doi:10.3390/cancers14153782)

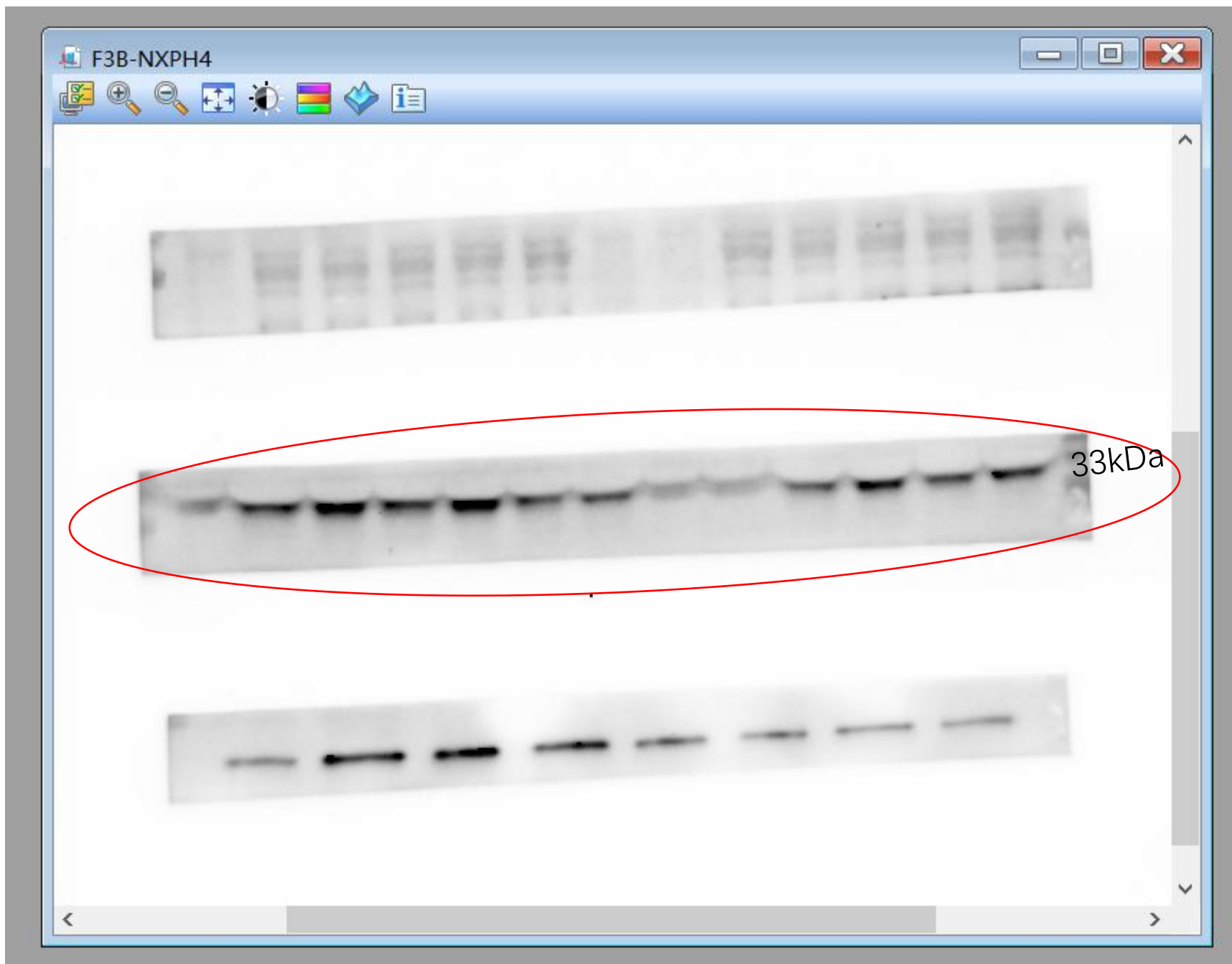

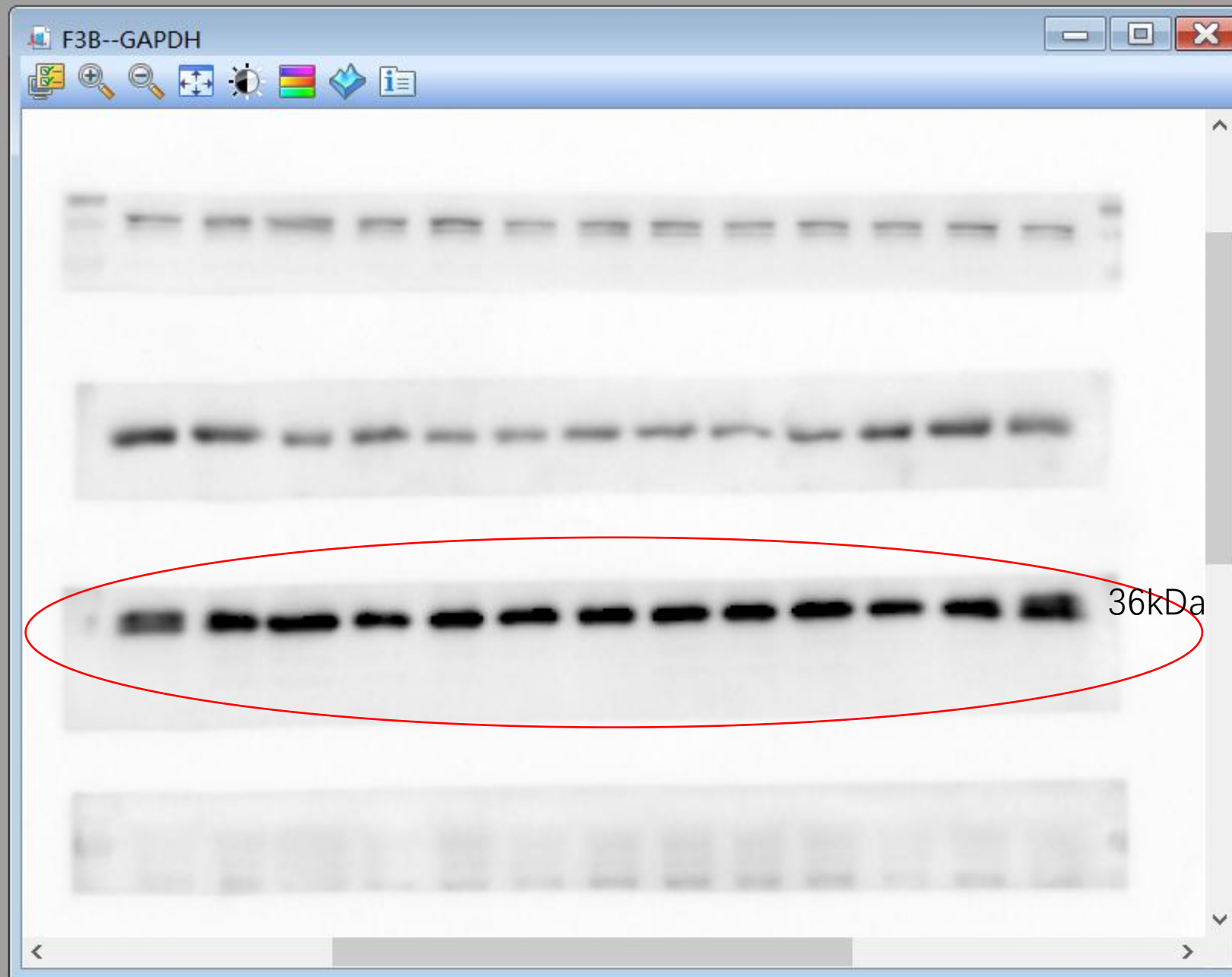

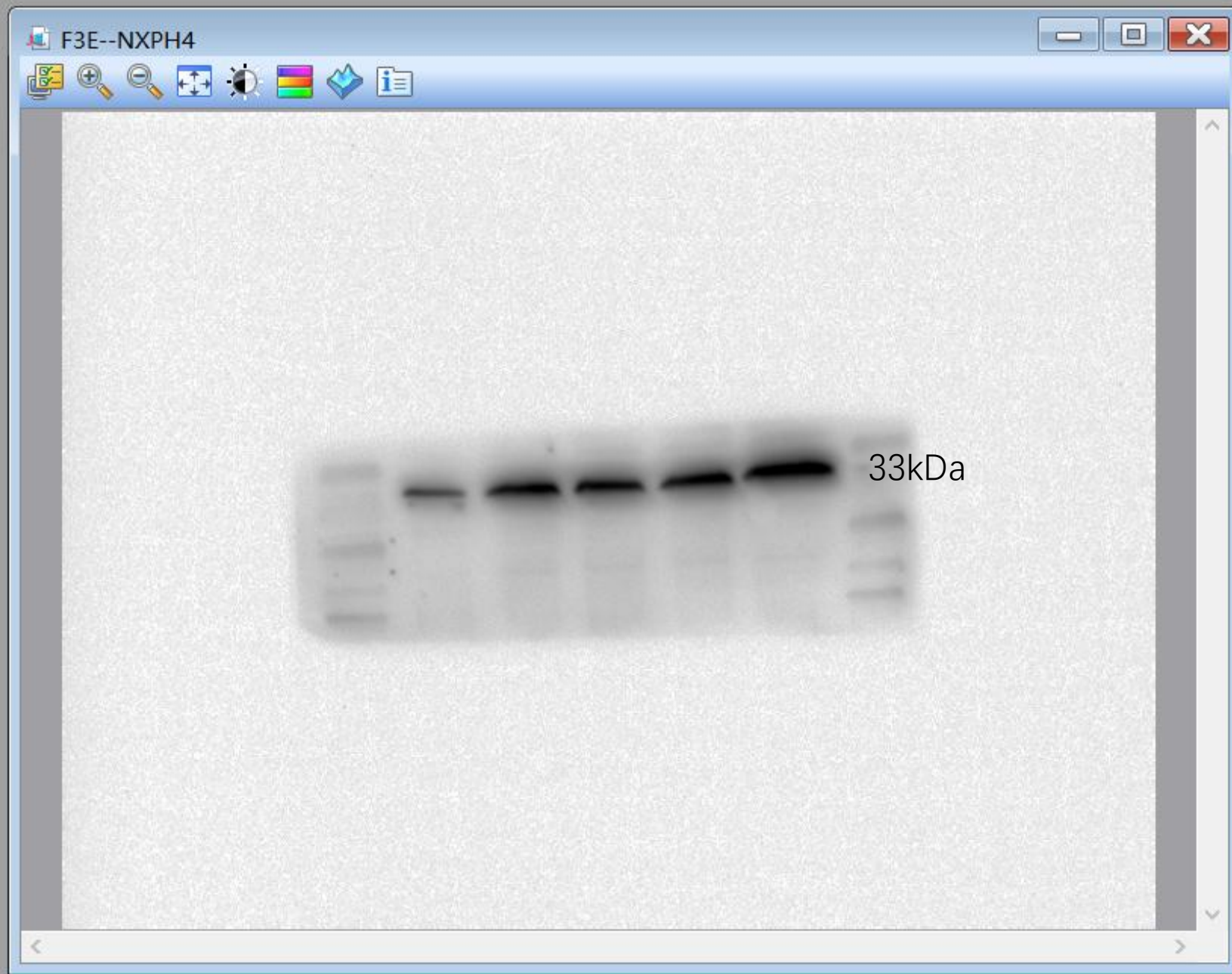

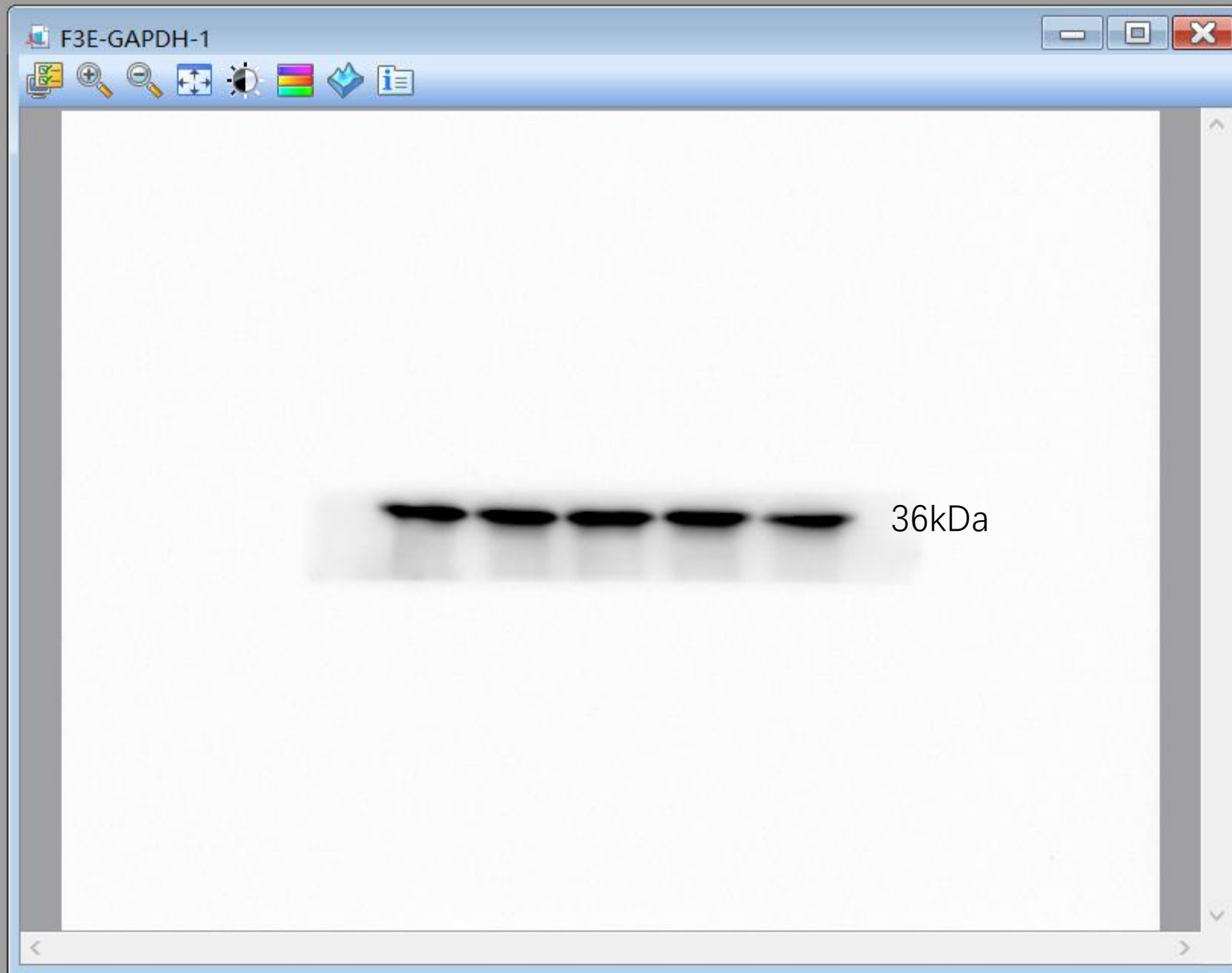

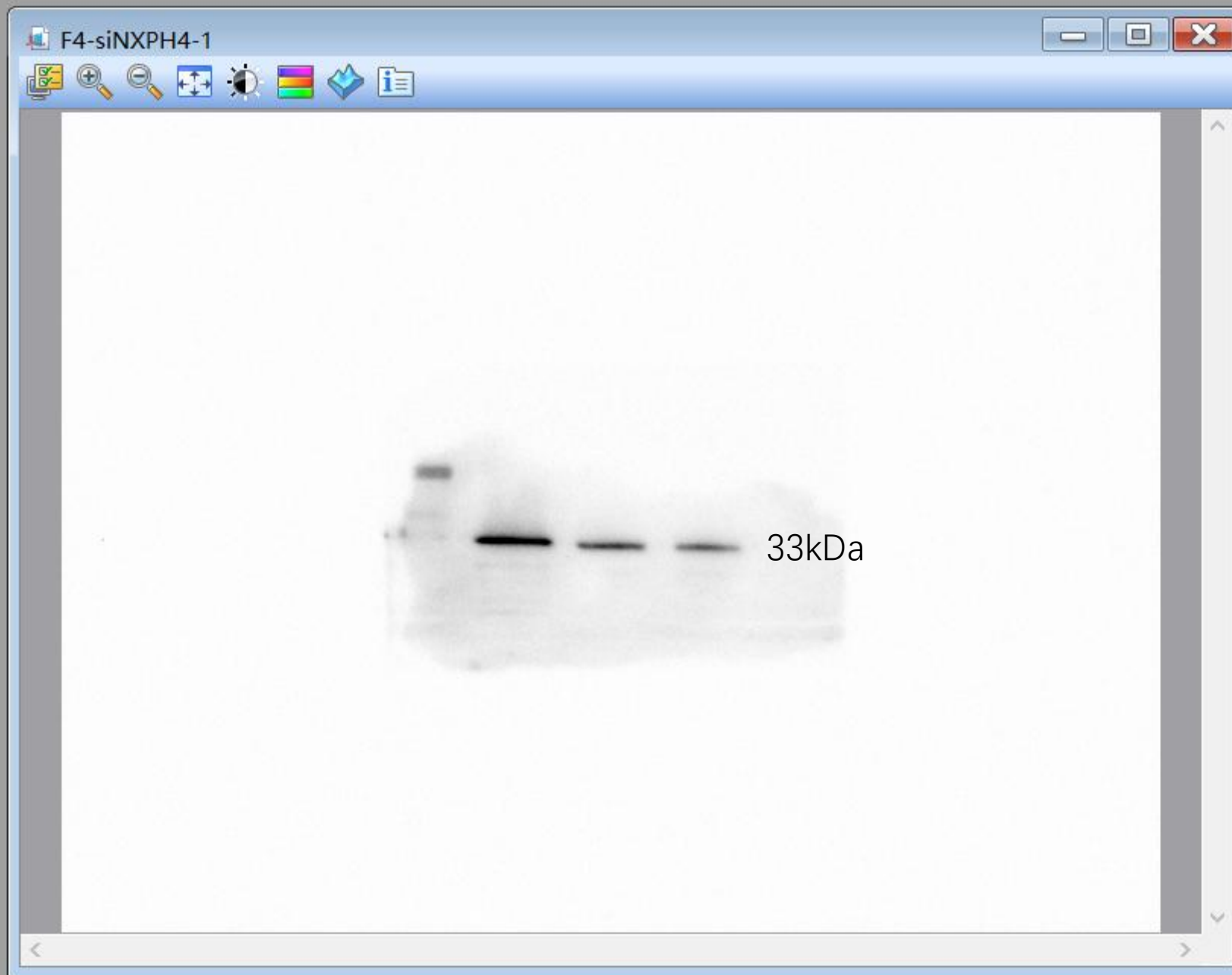

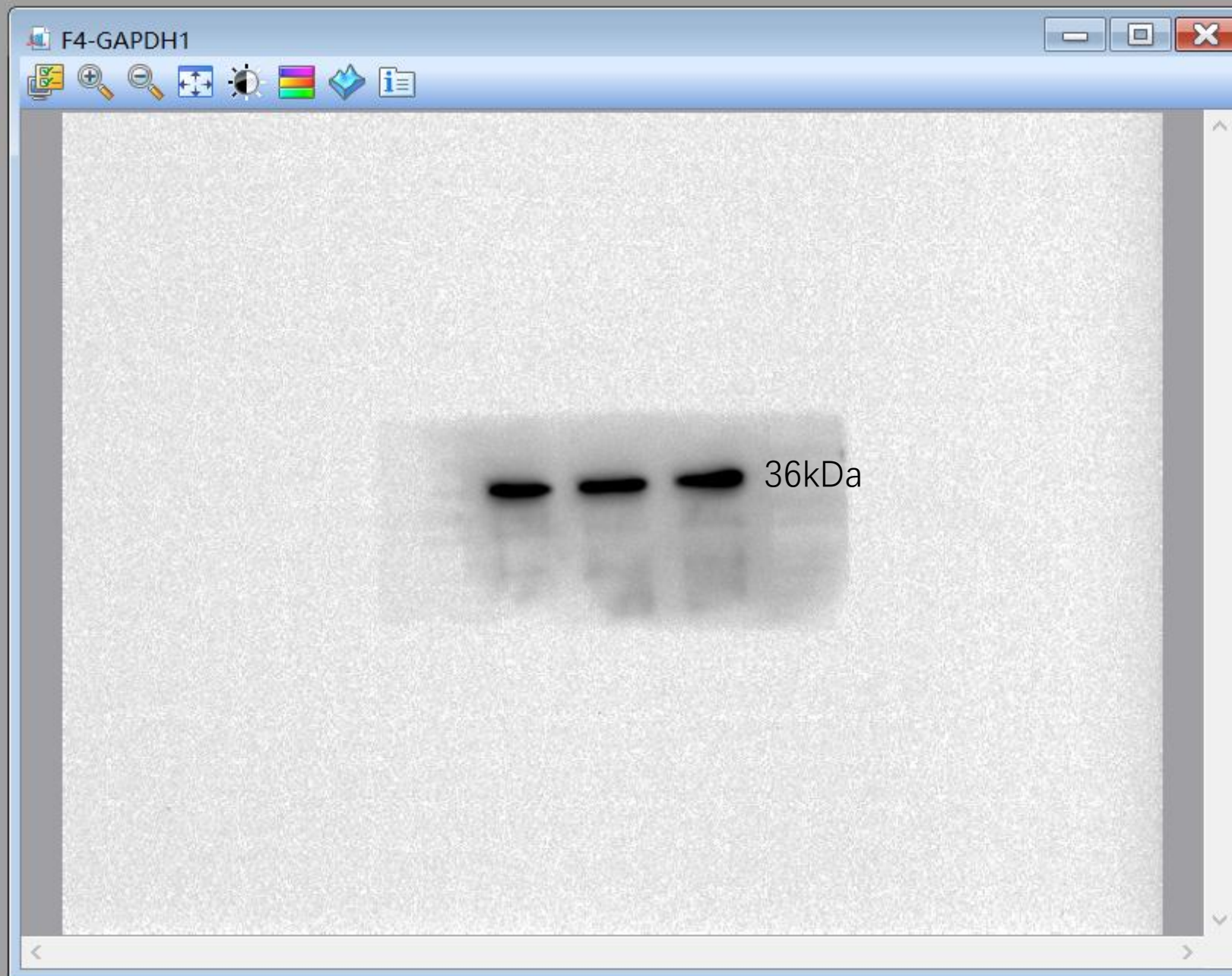

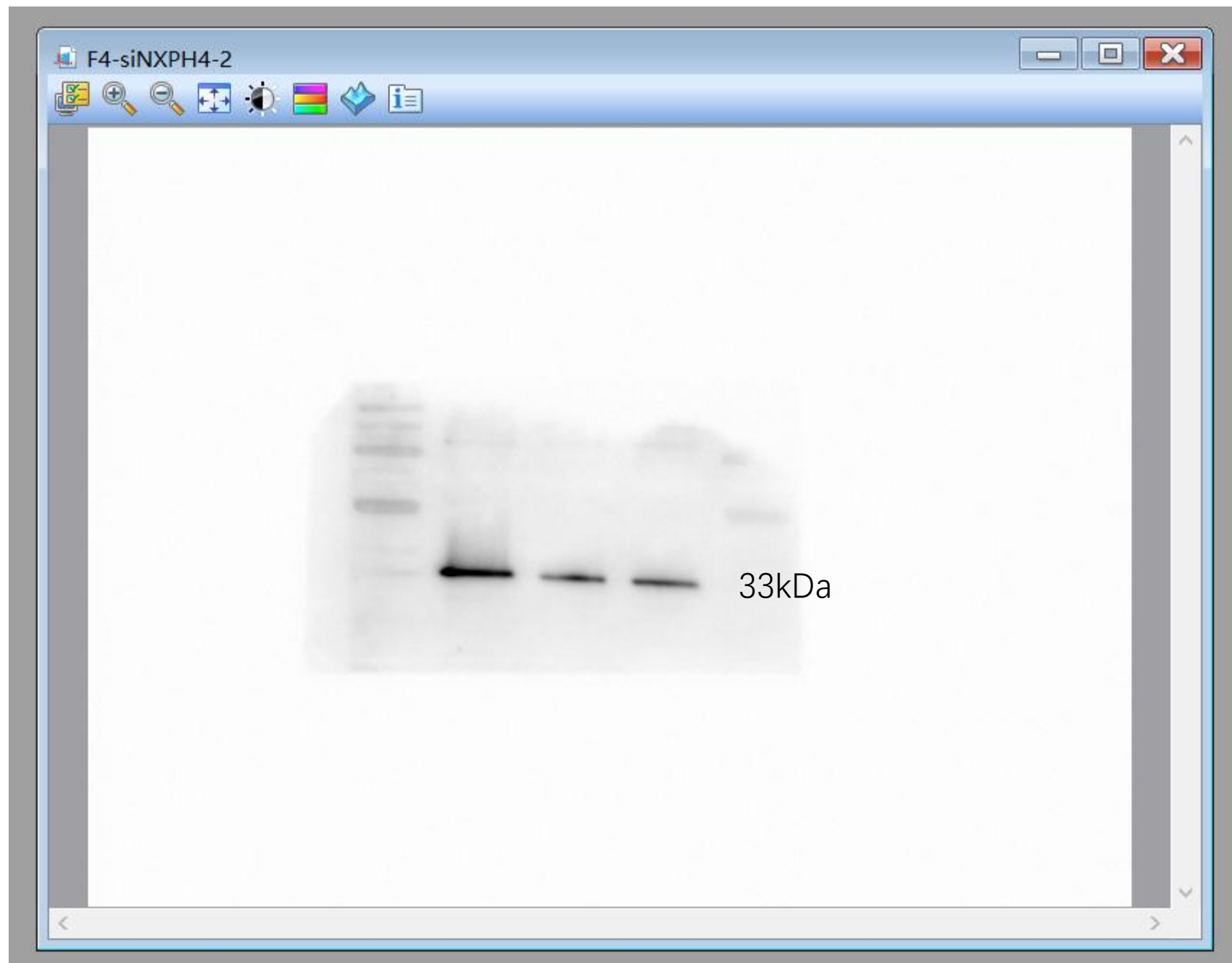

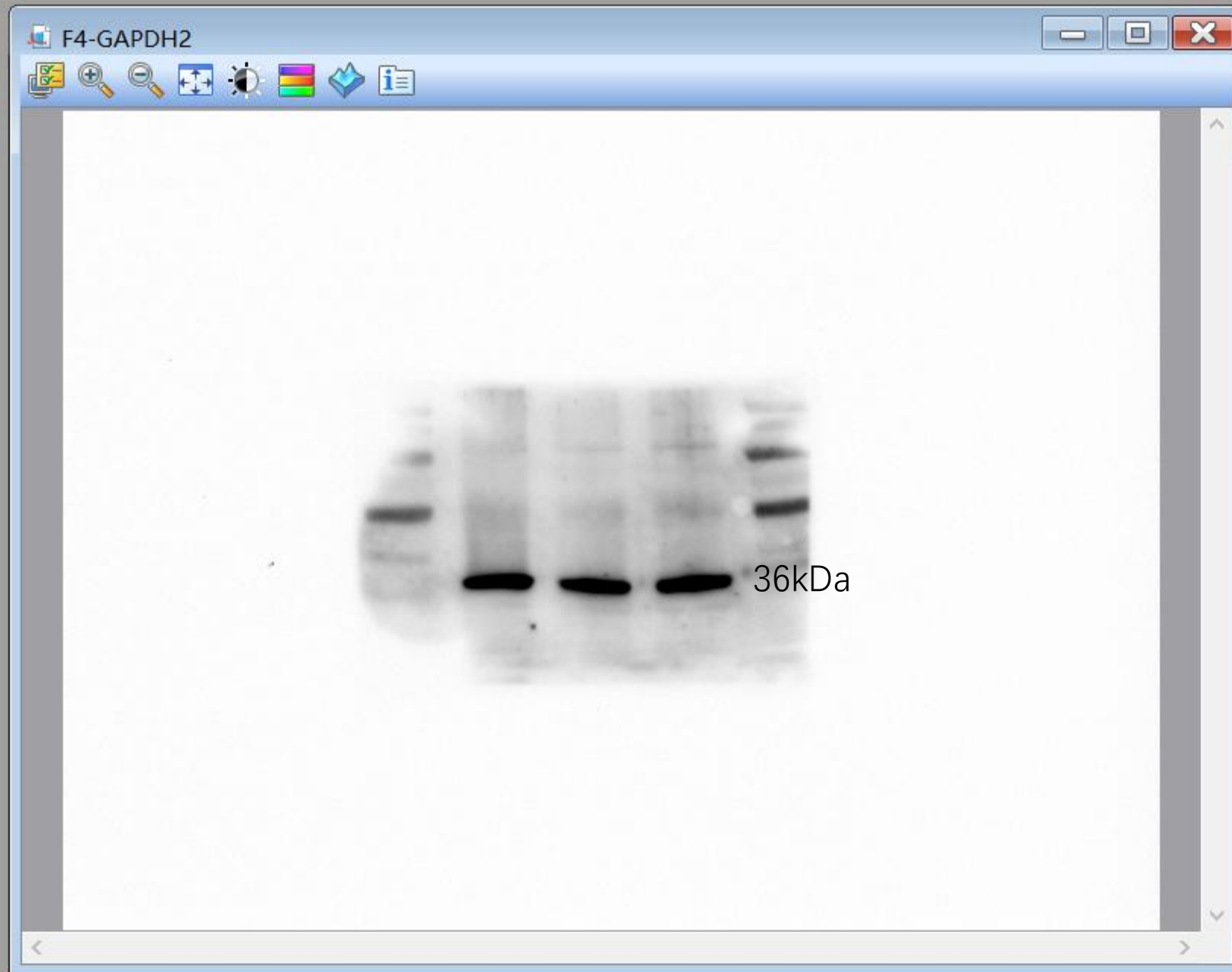

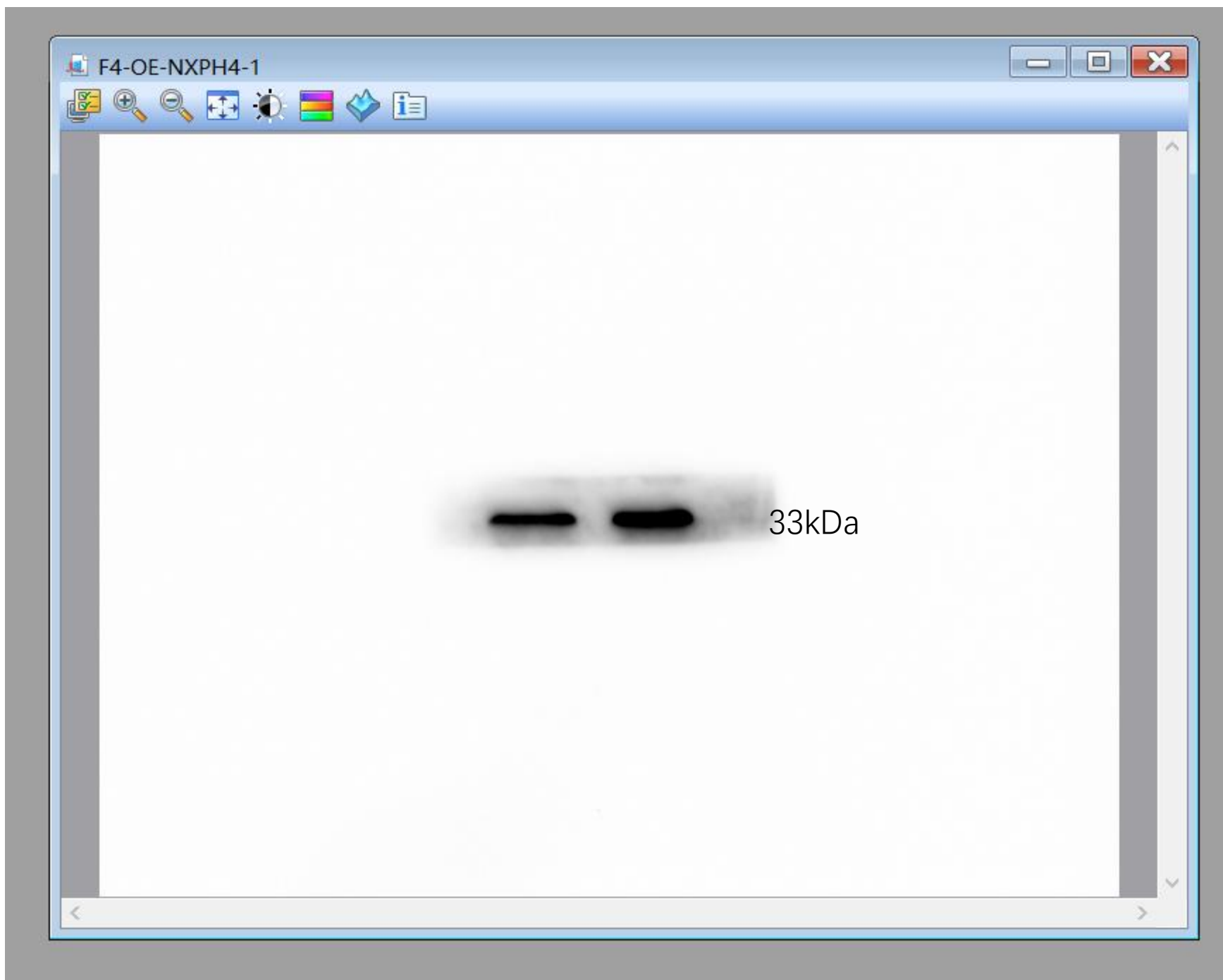

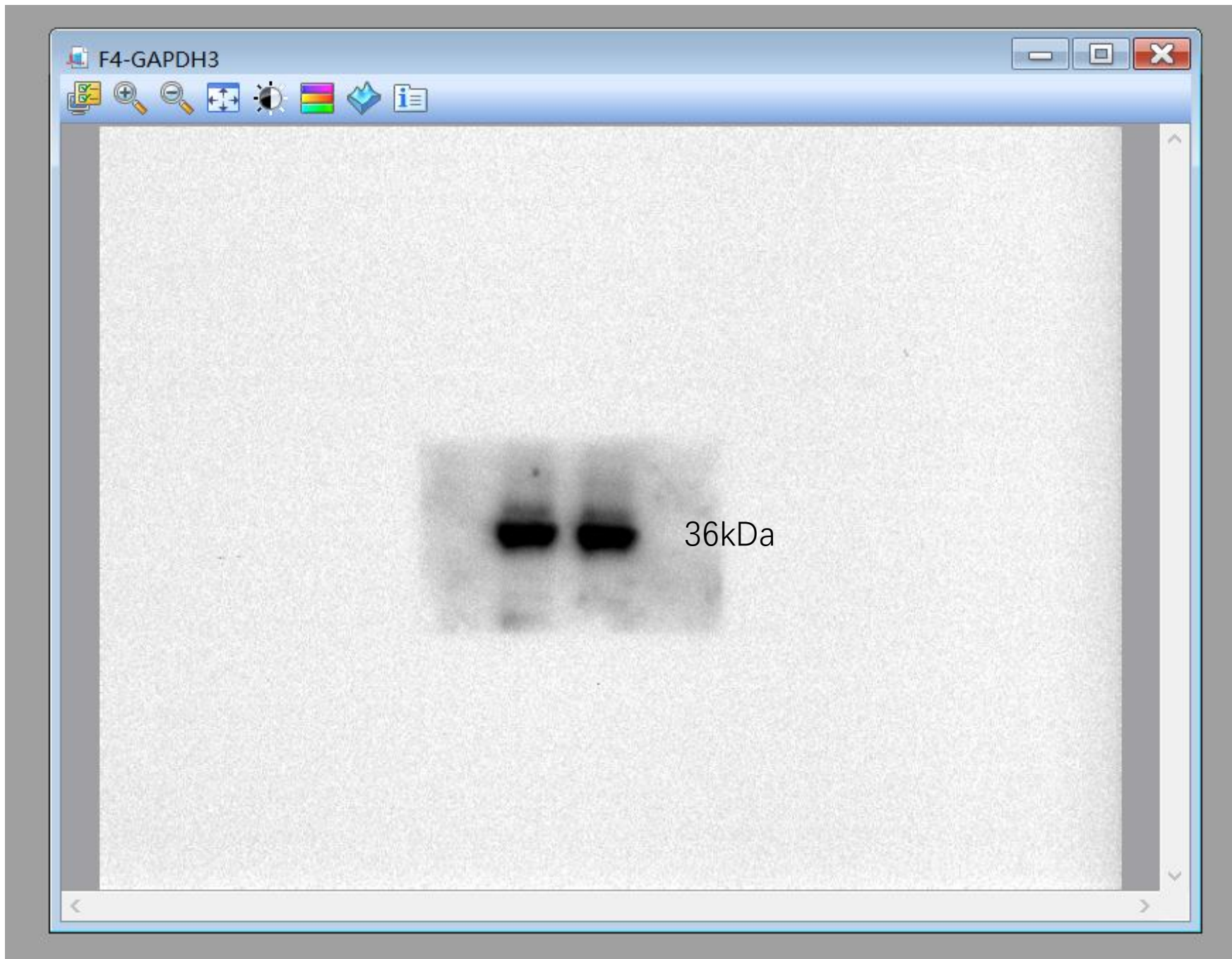

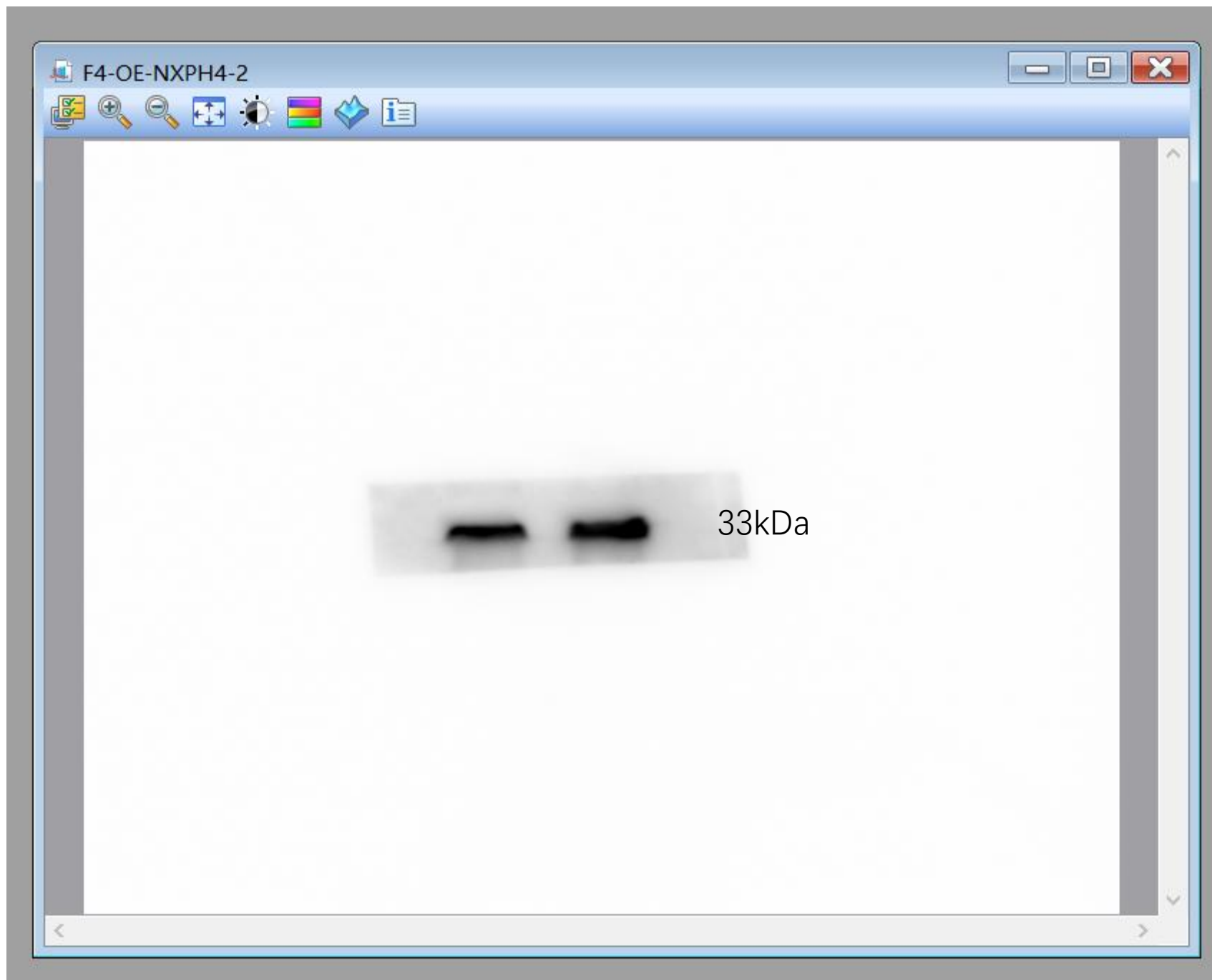

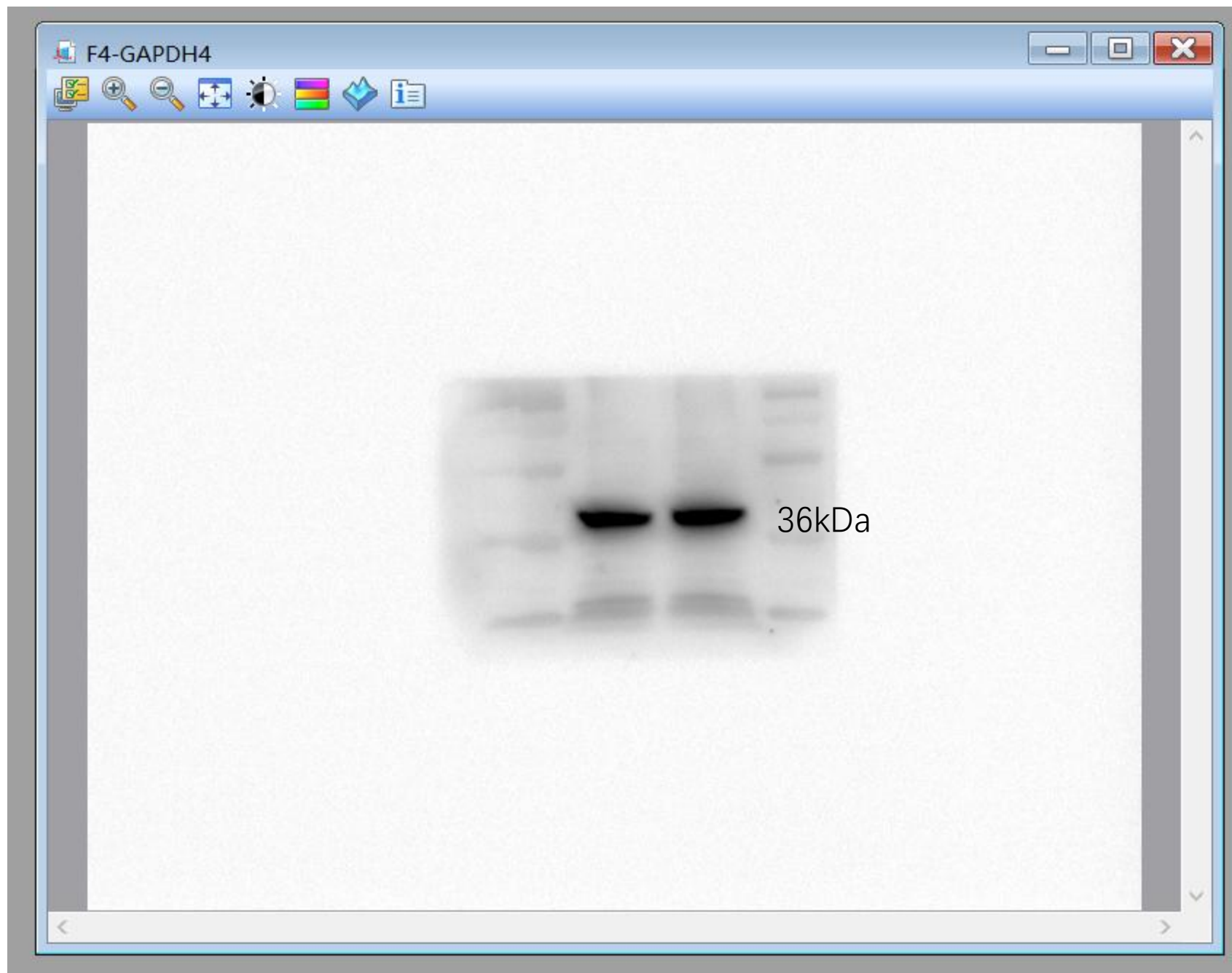

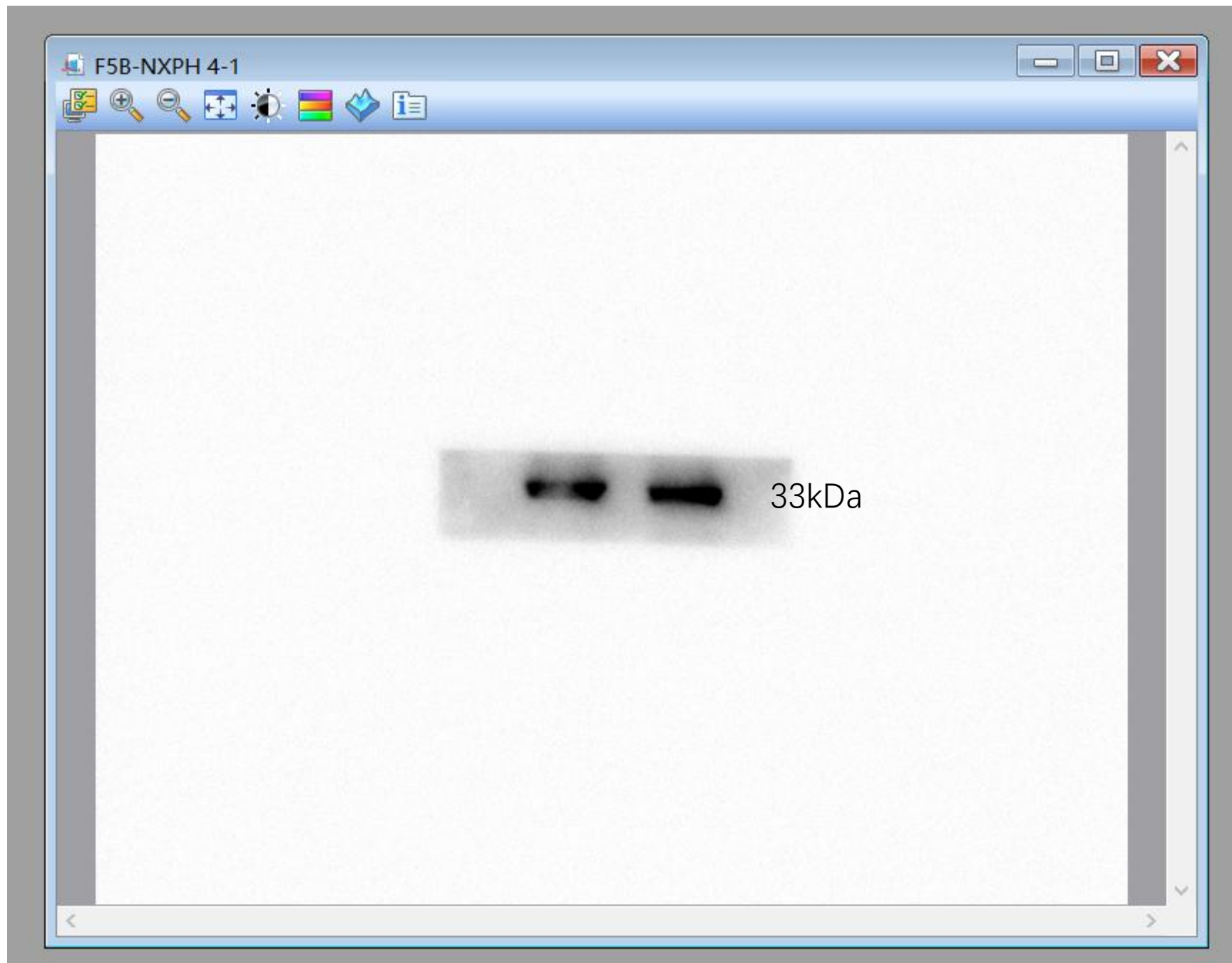

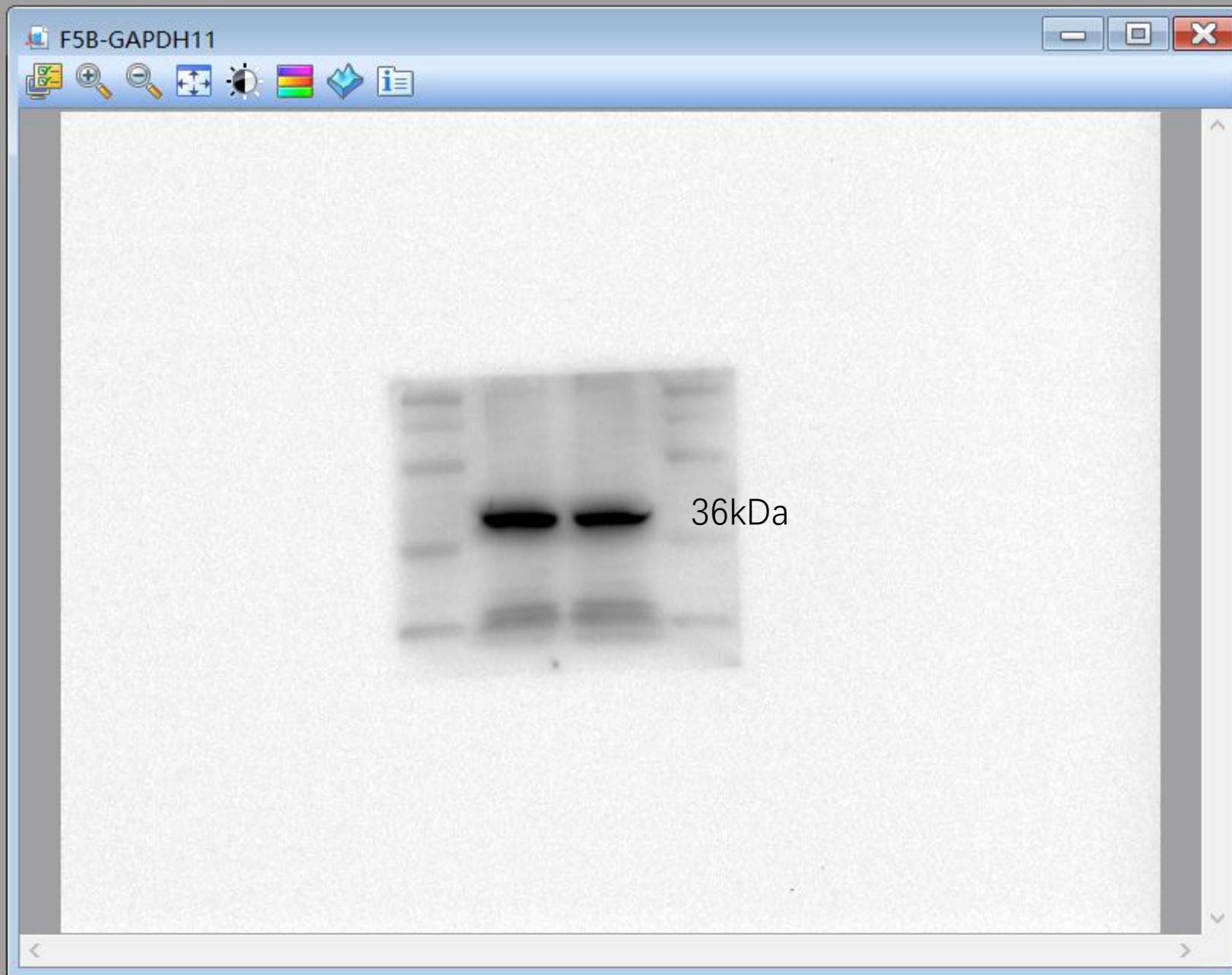

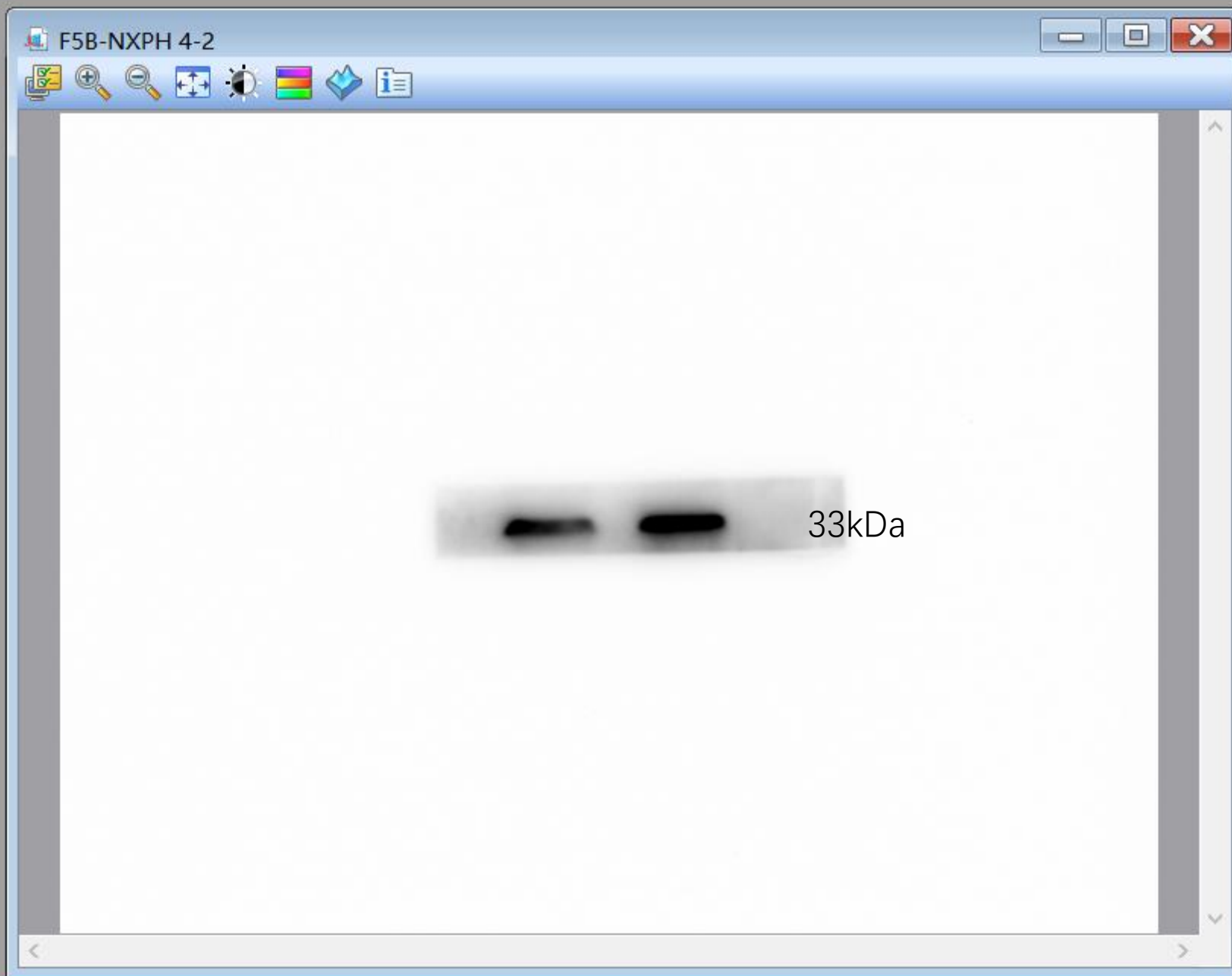

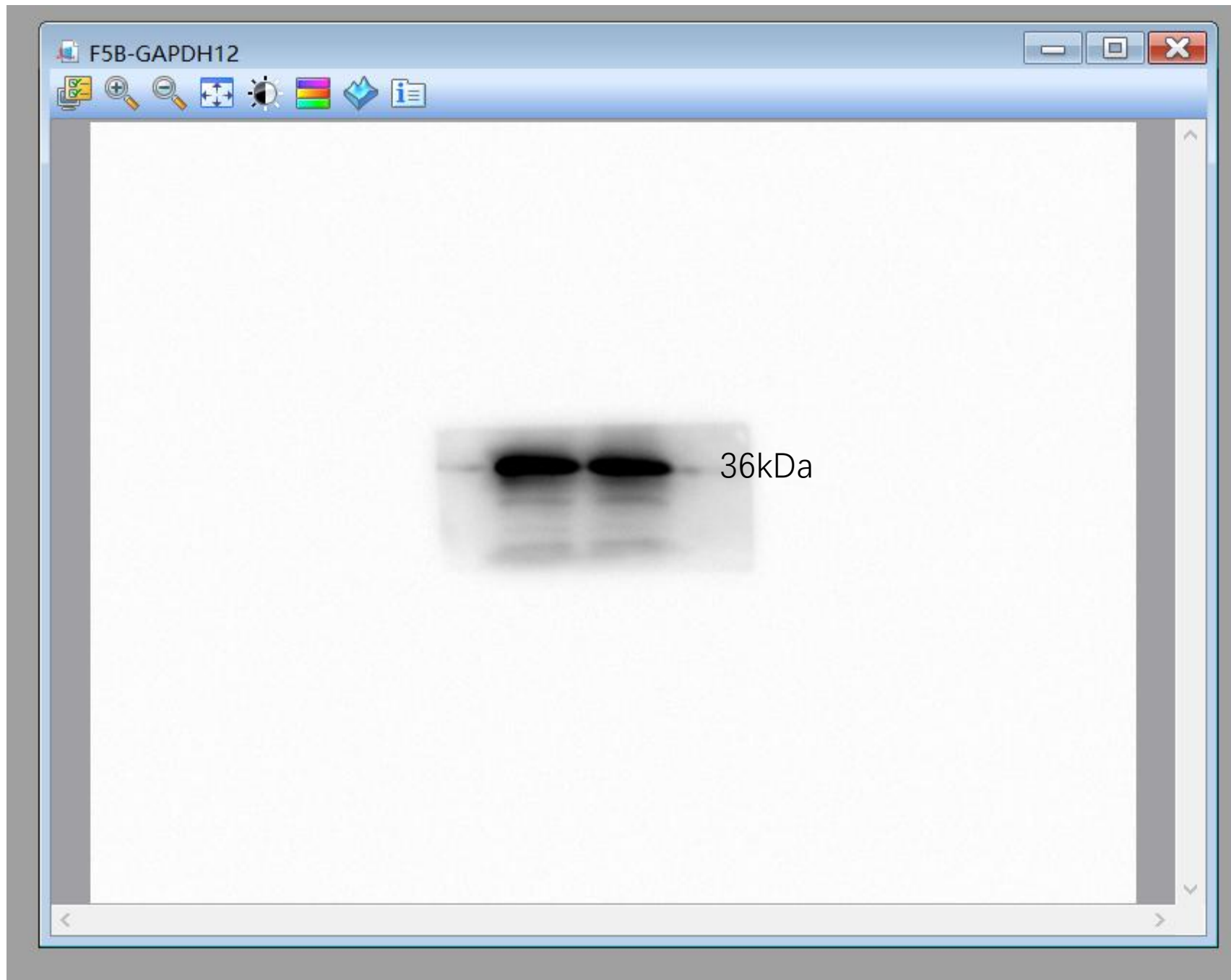

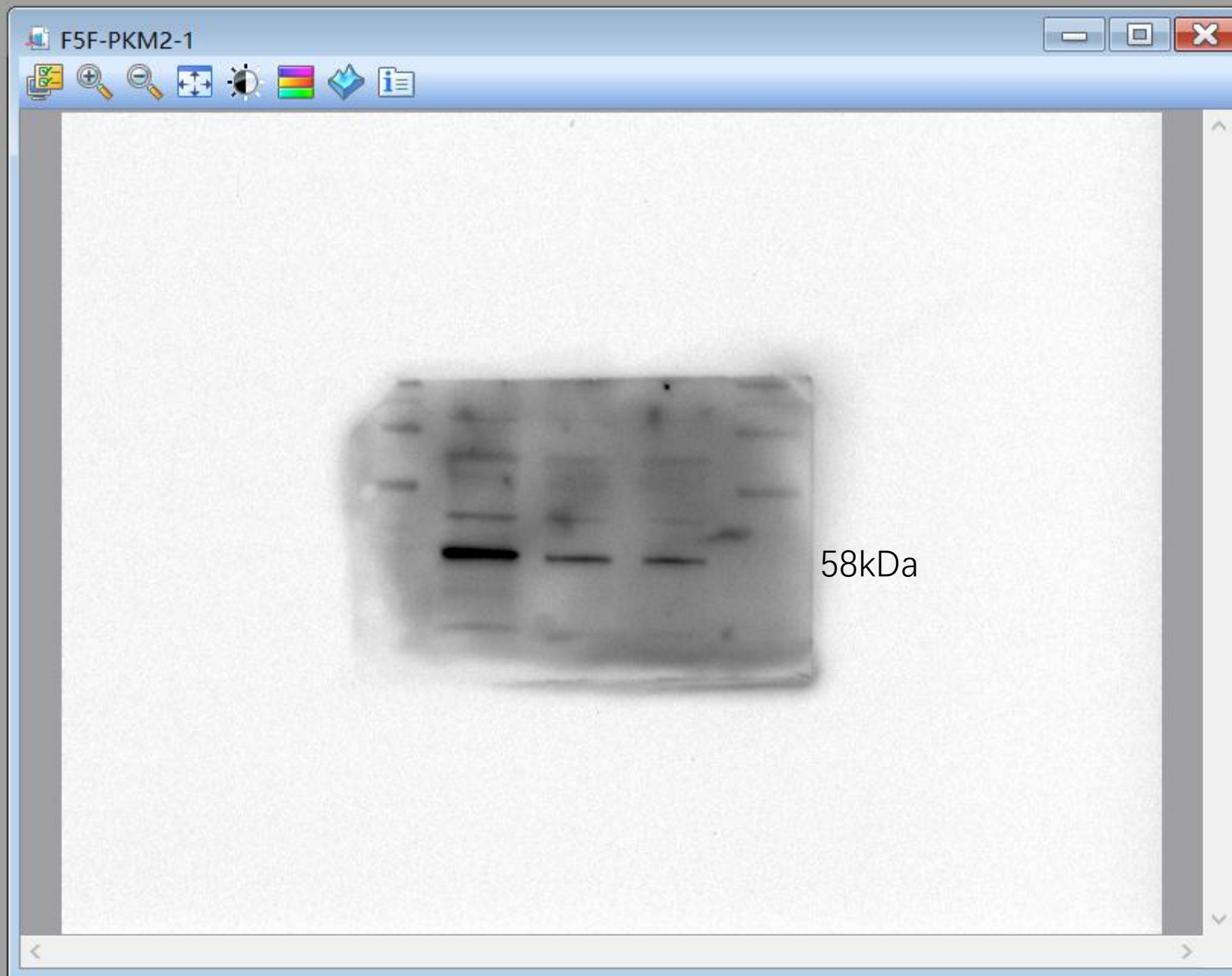

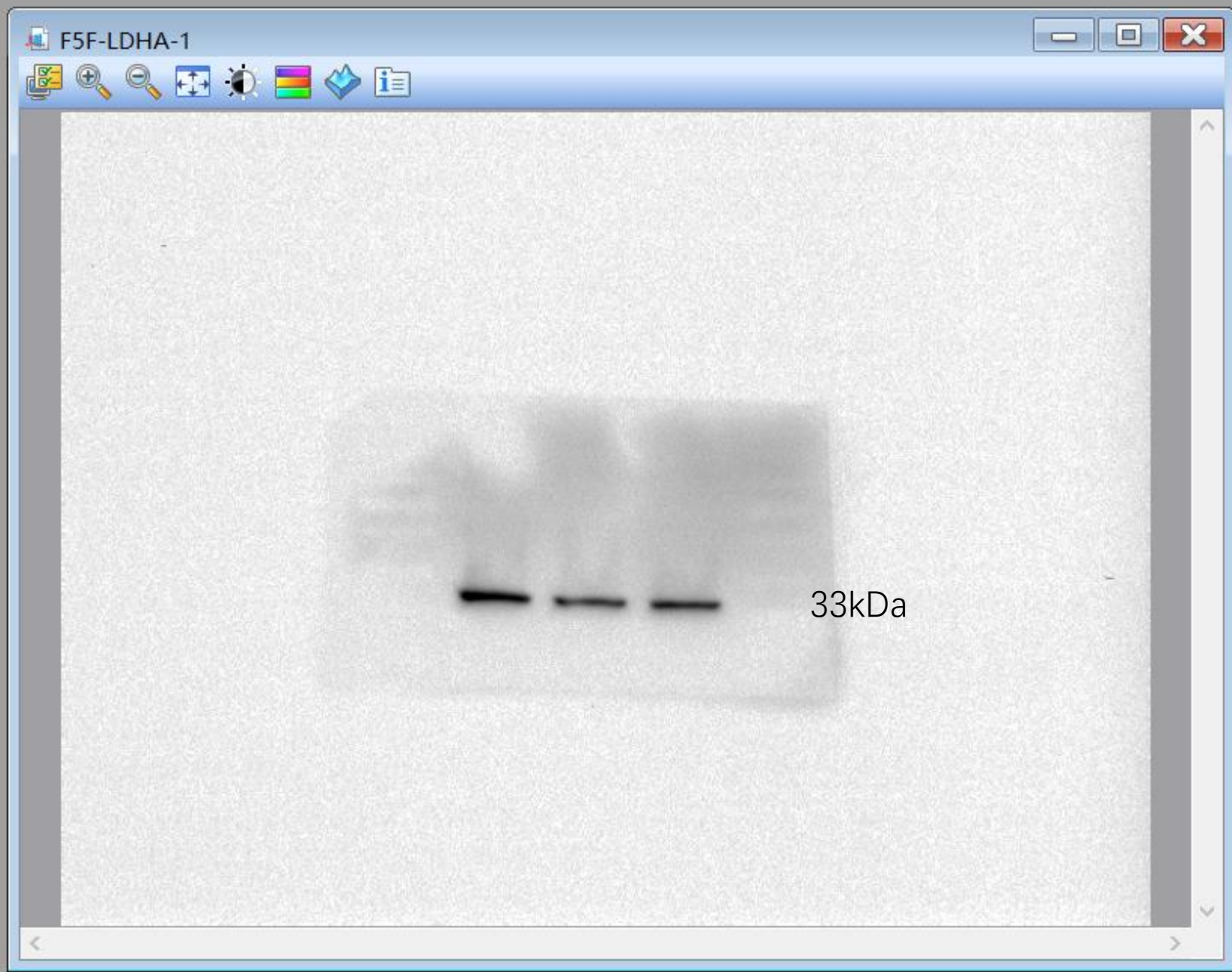

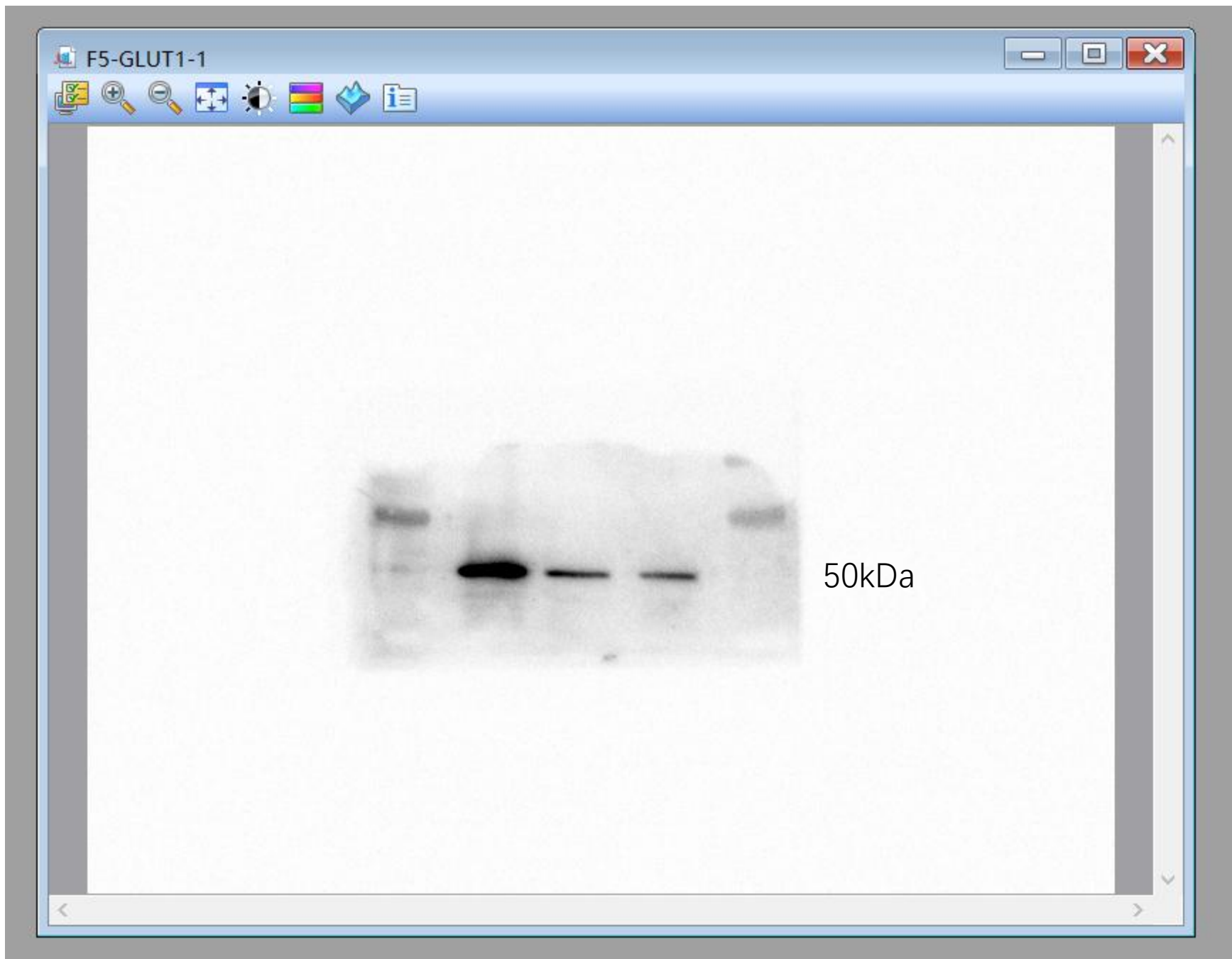

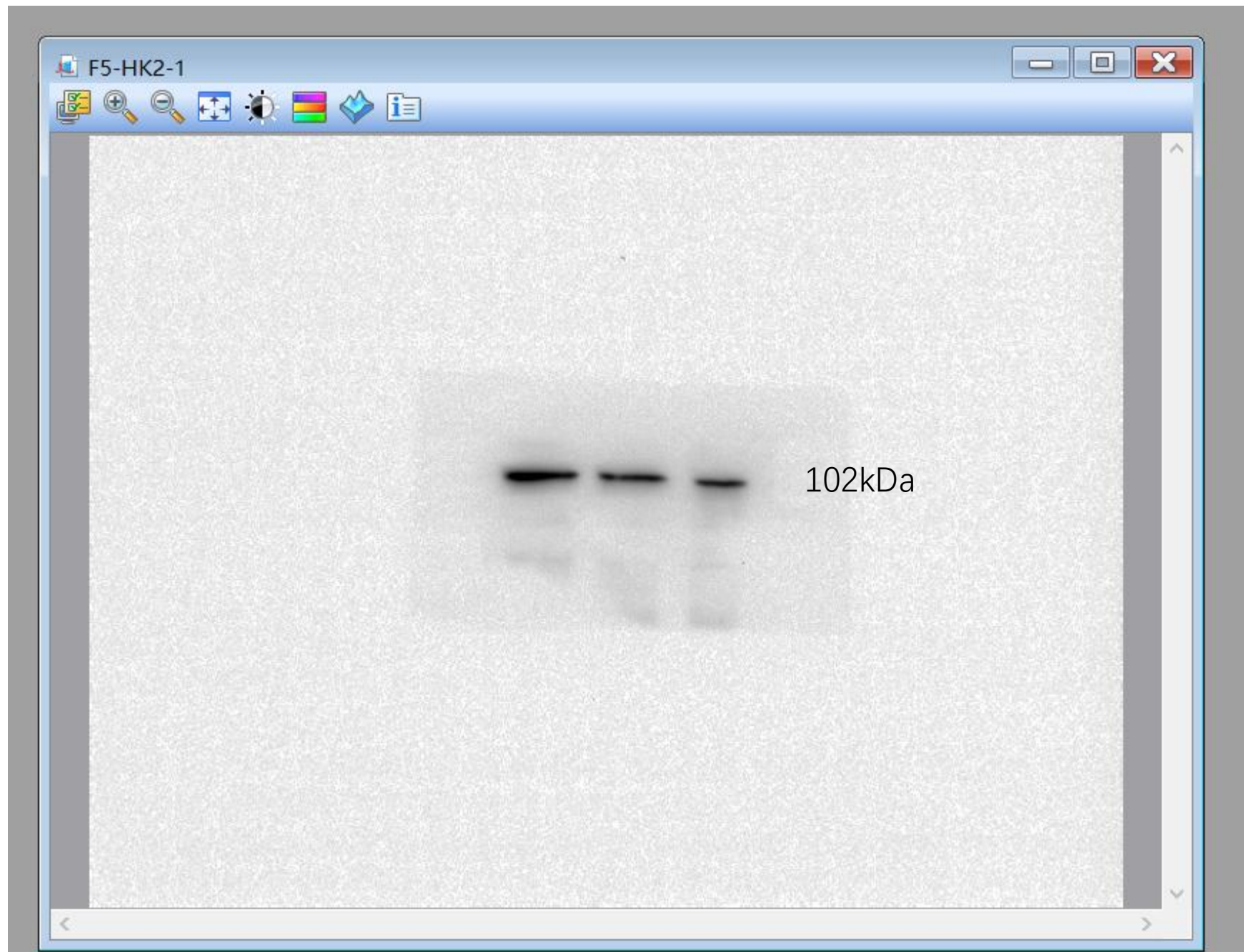

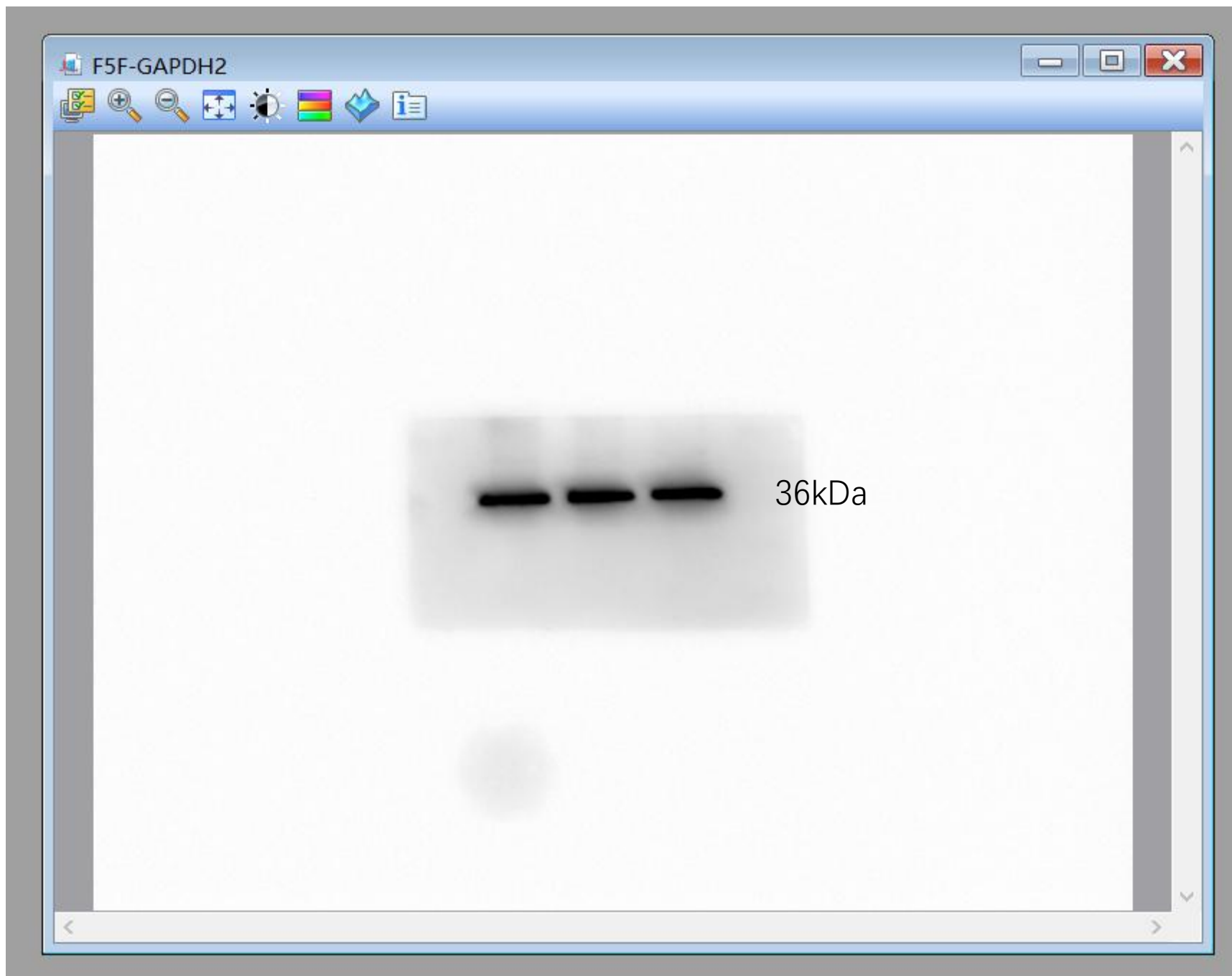

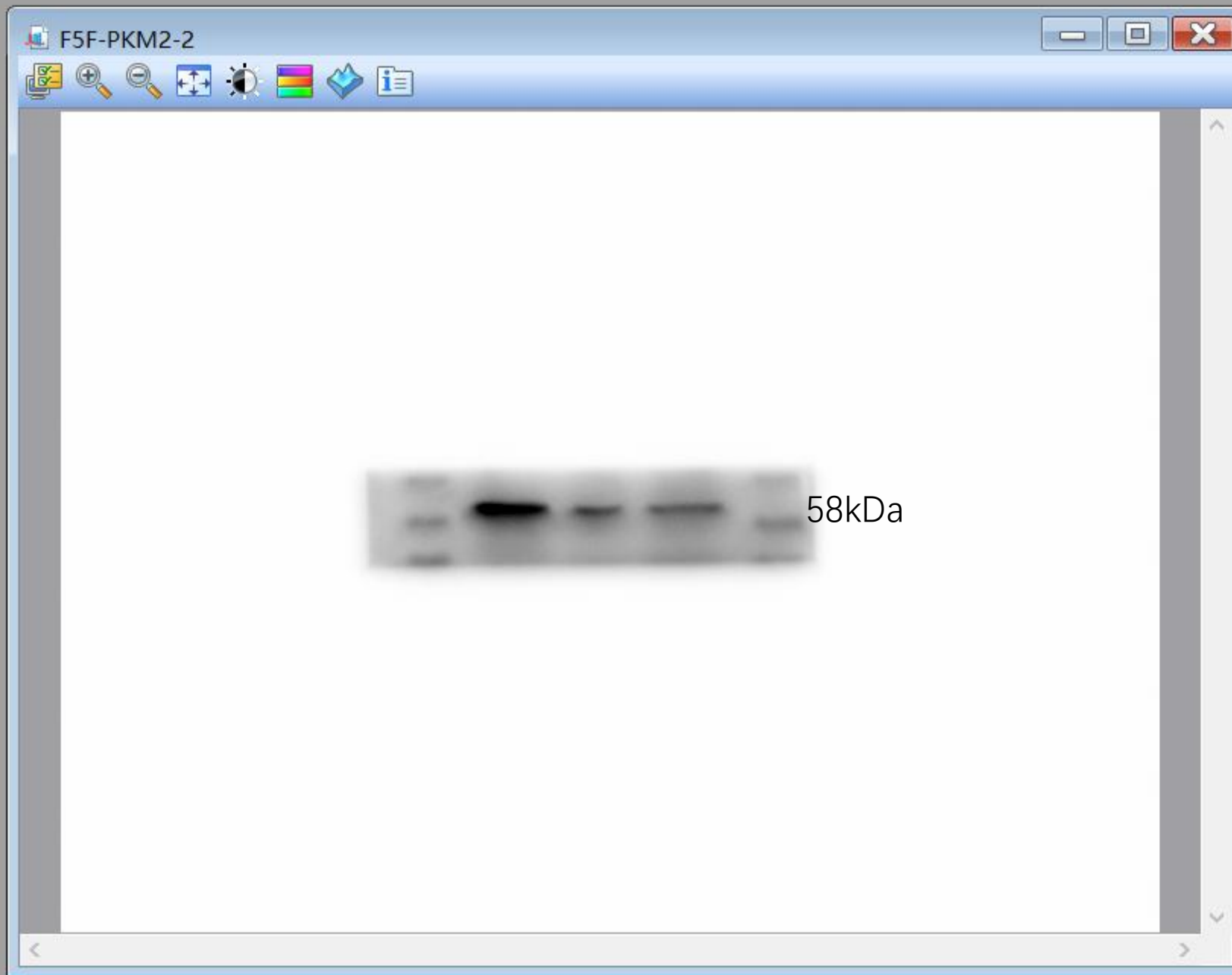

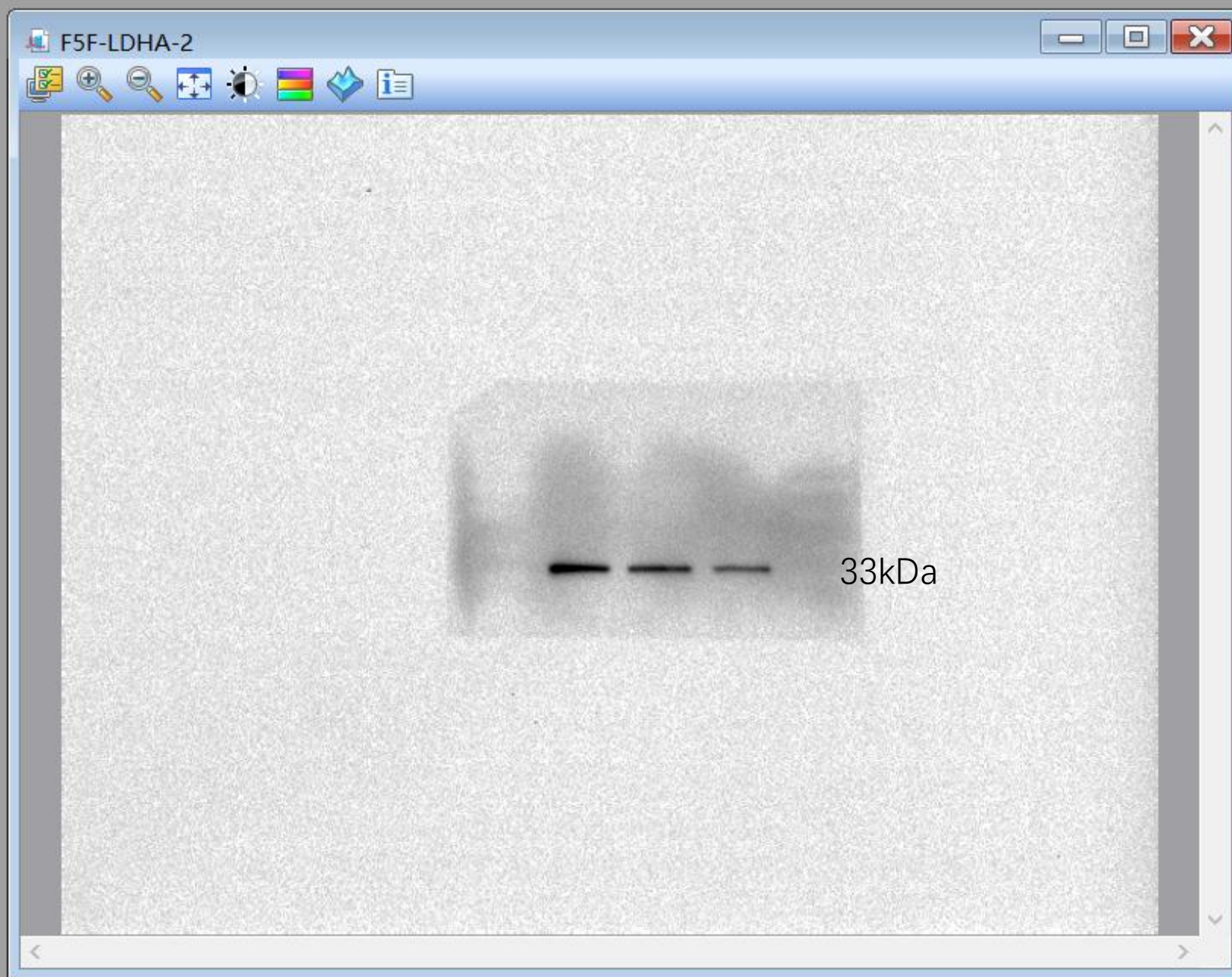

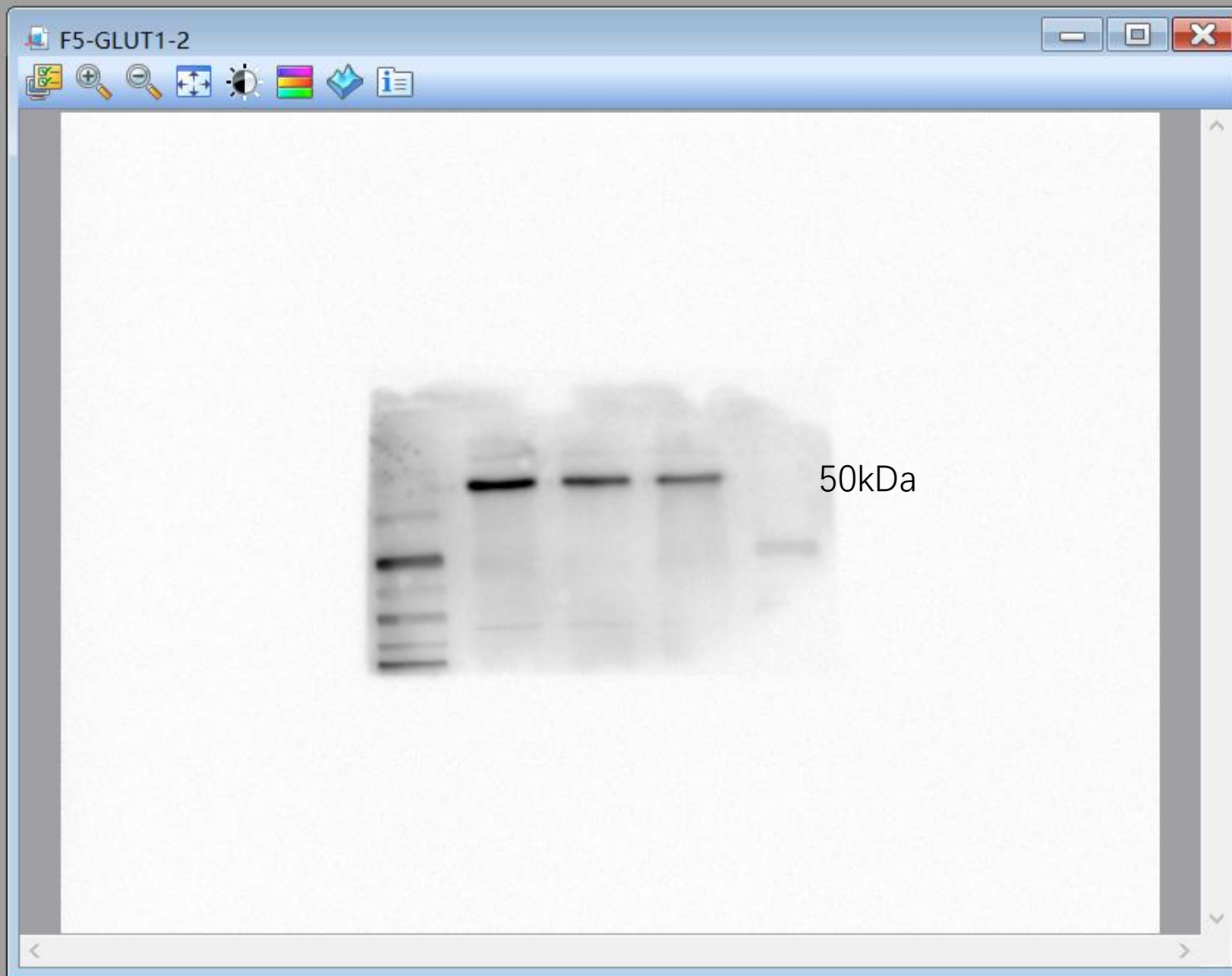

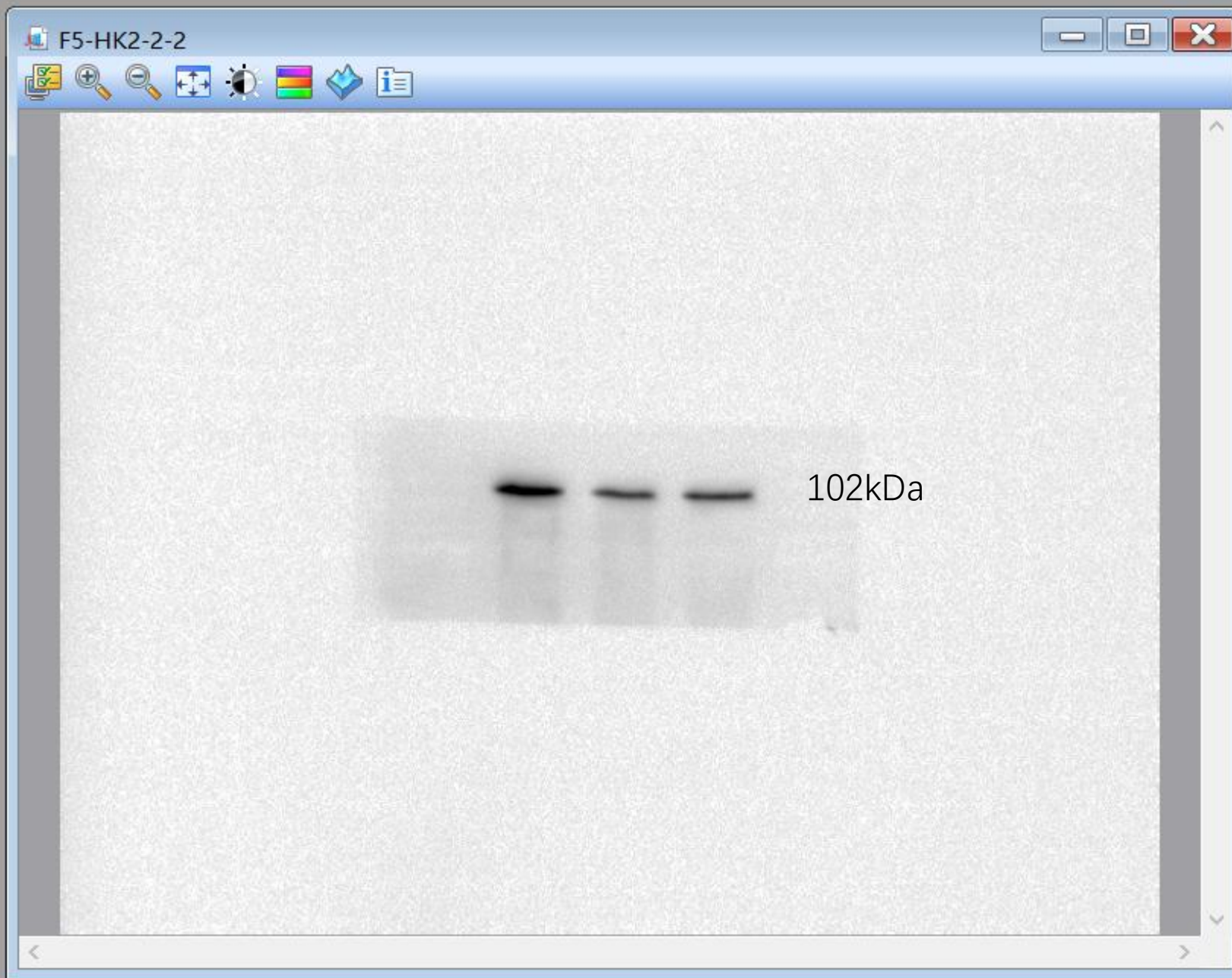

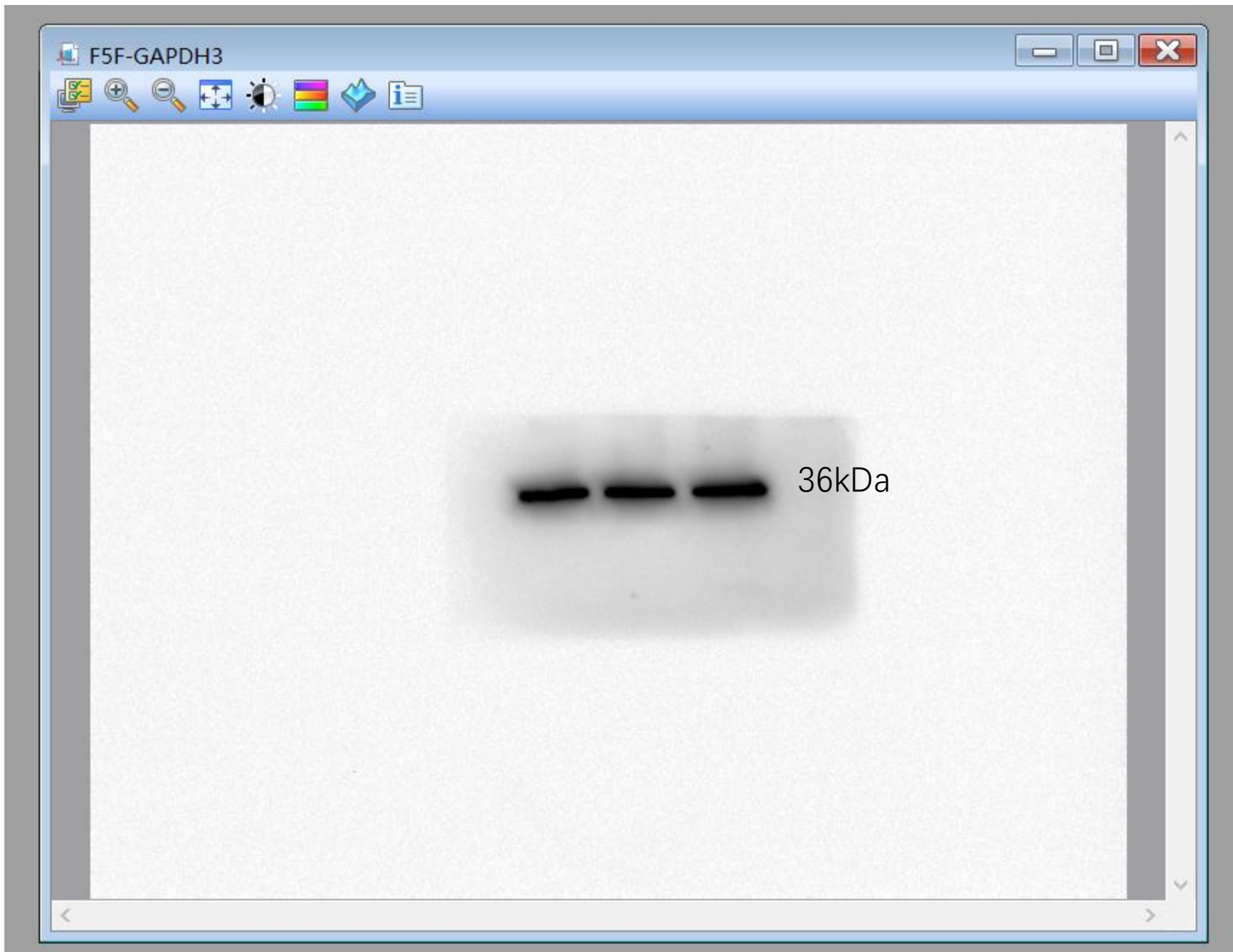

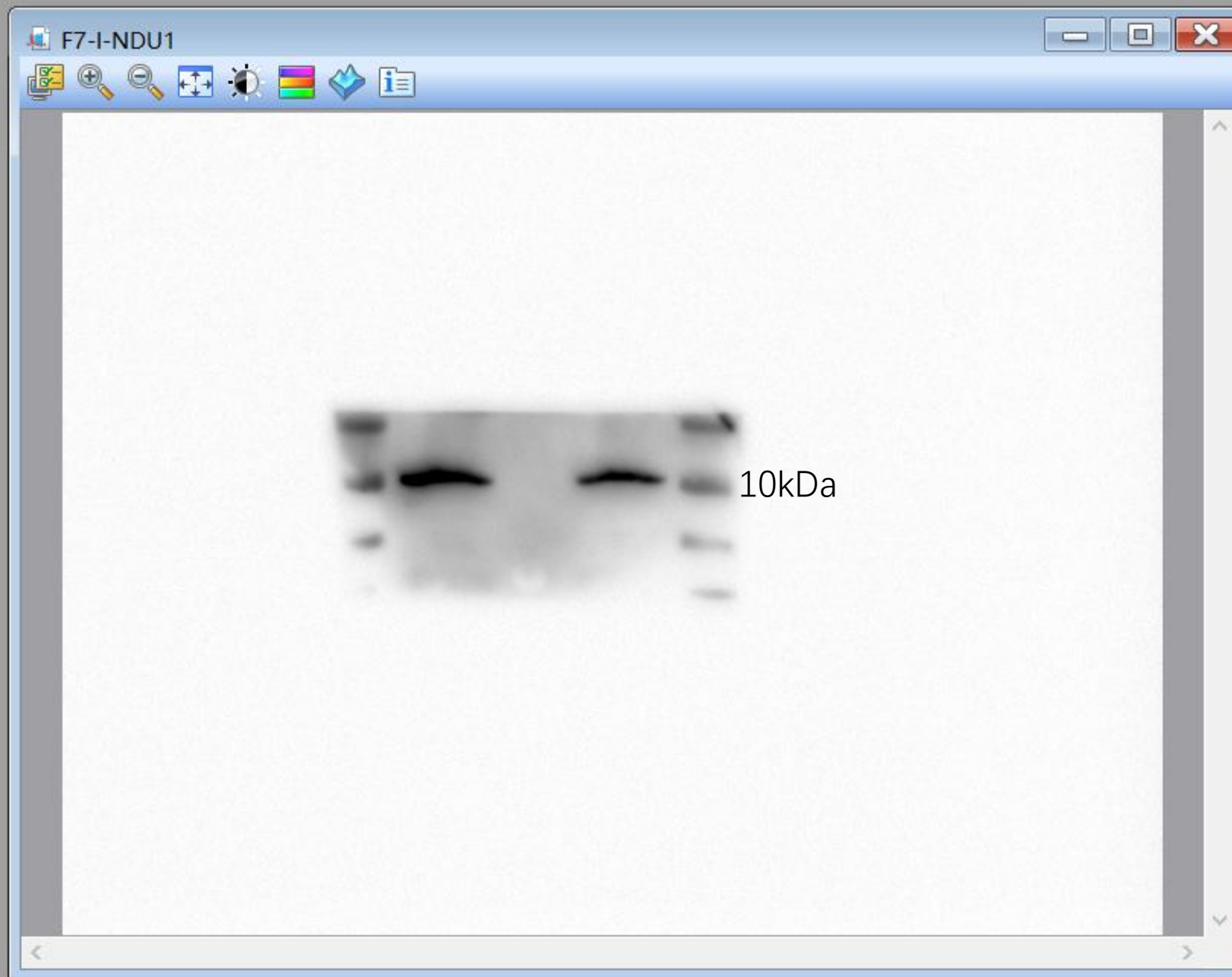

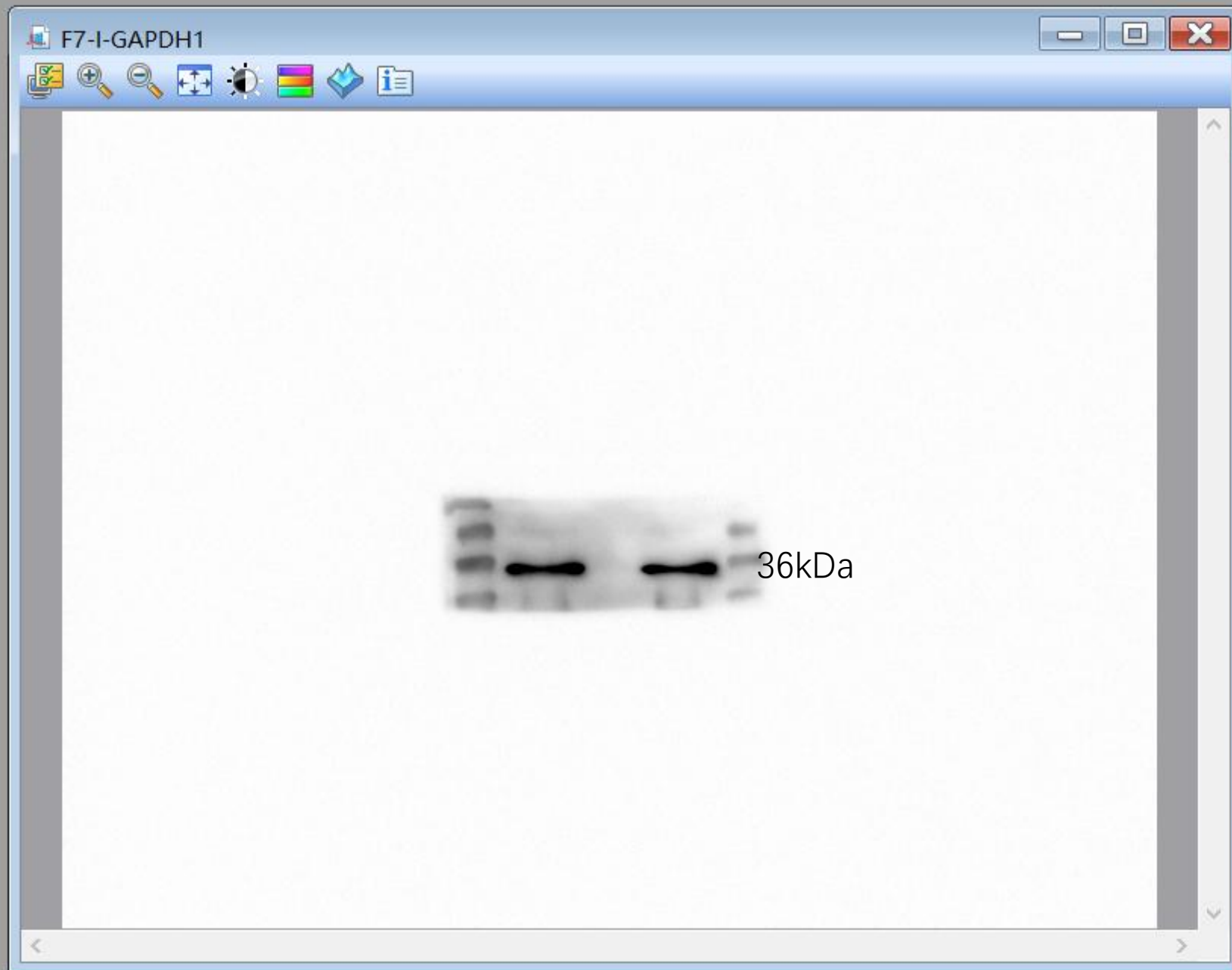

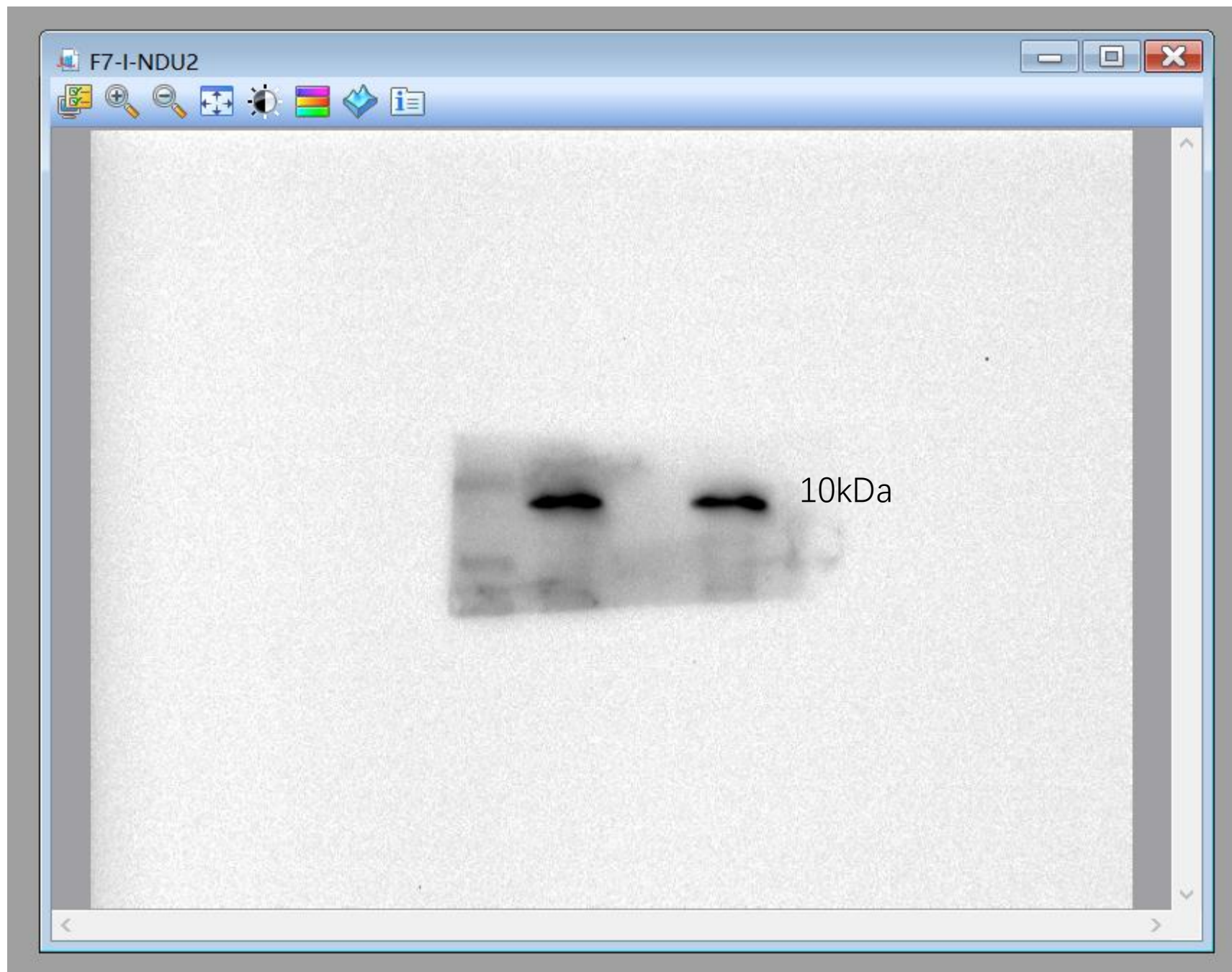

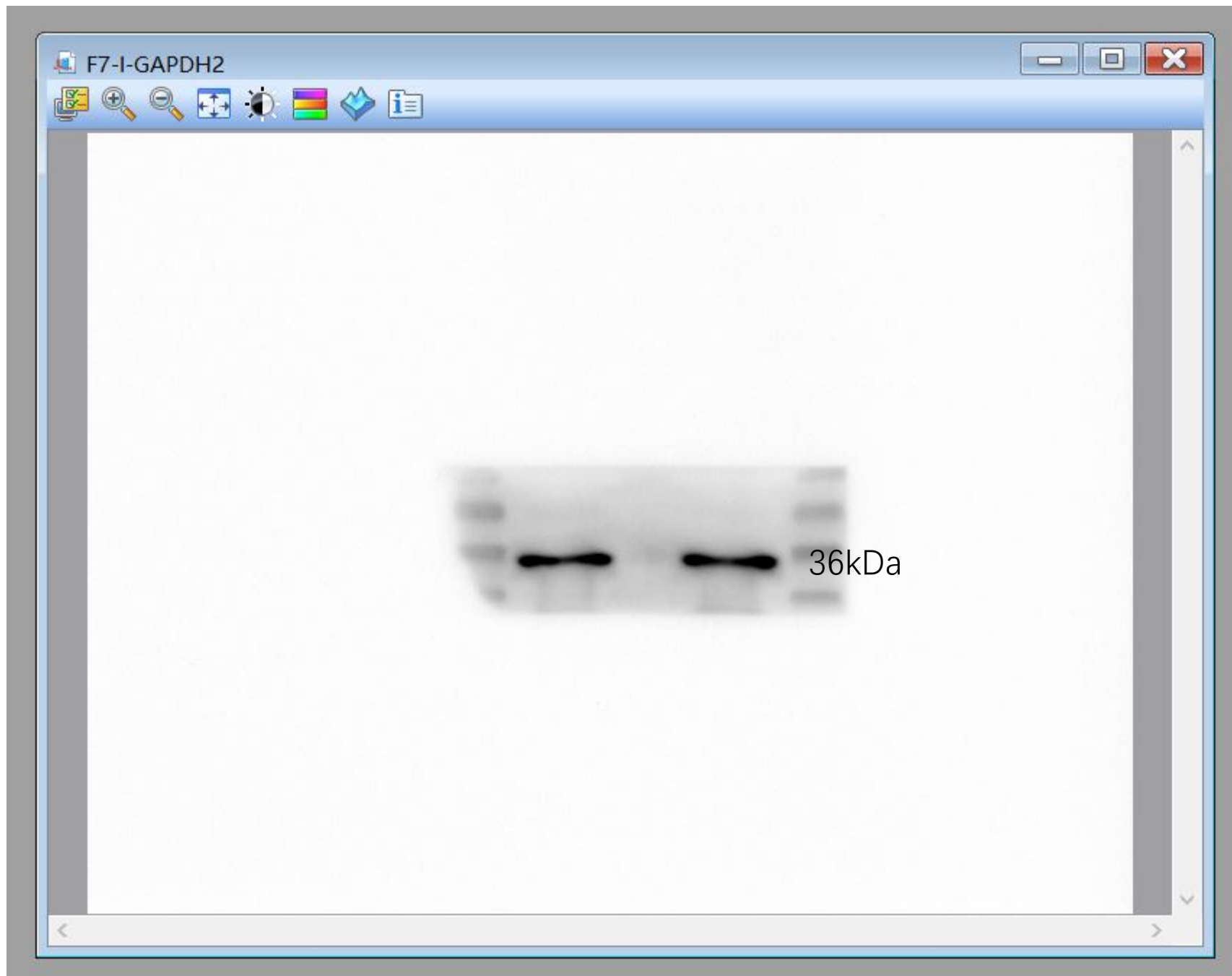

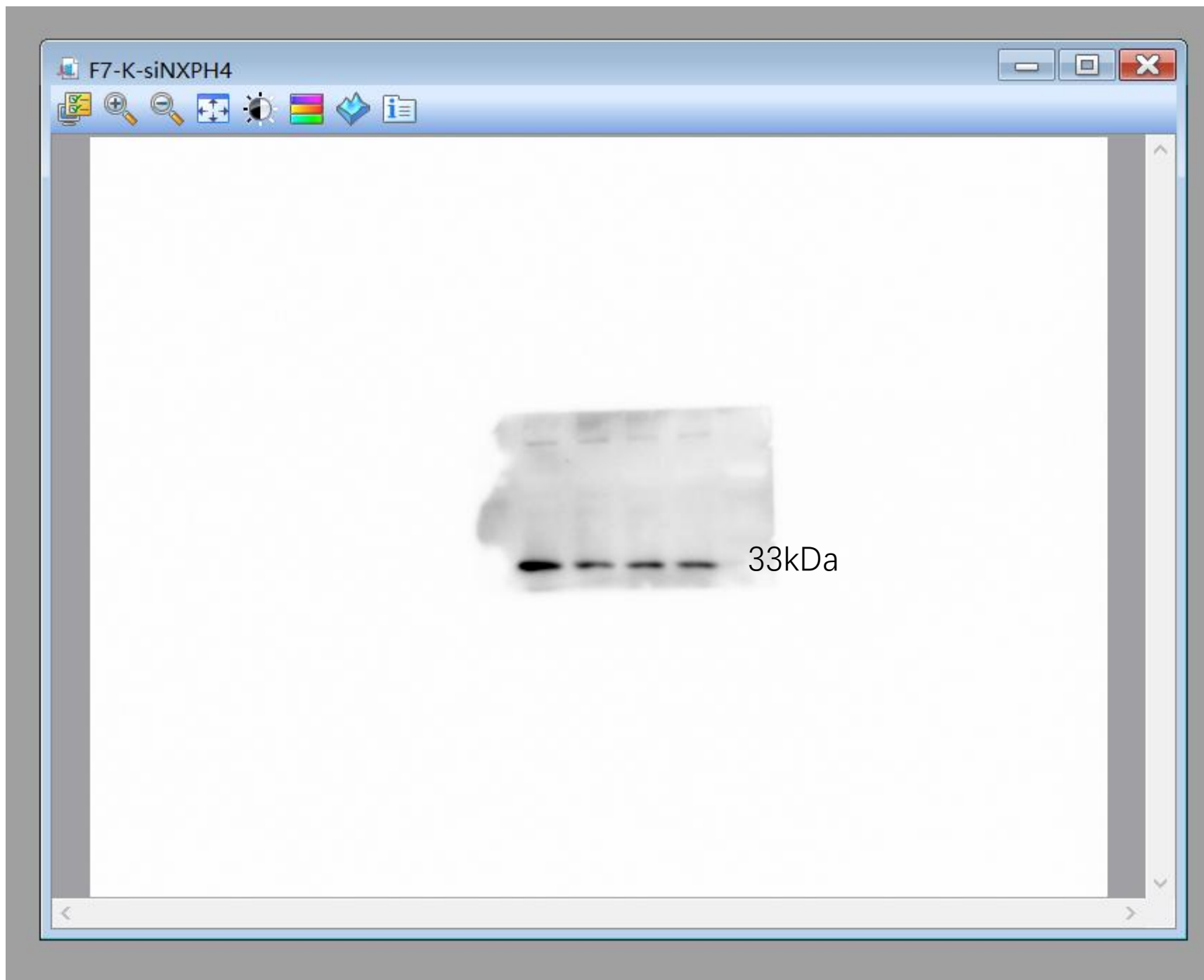

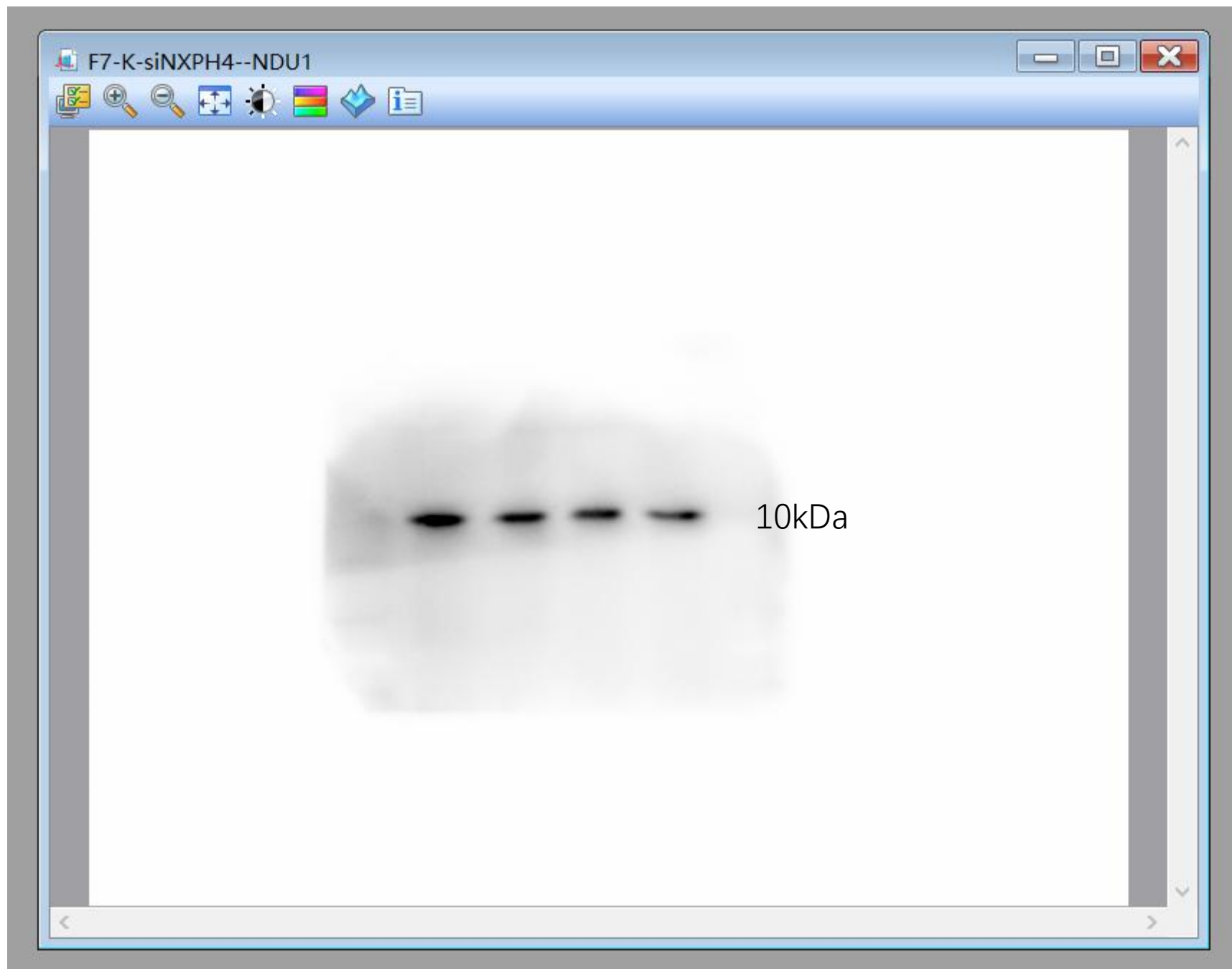

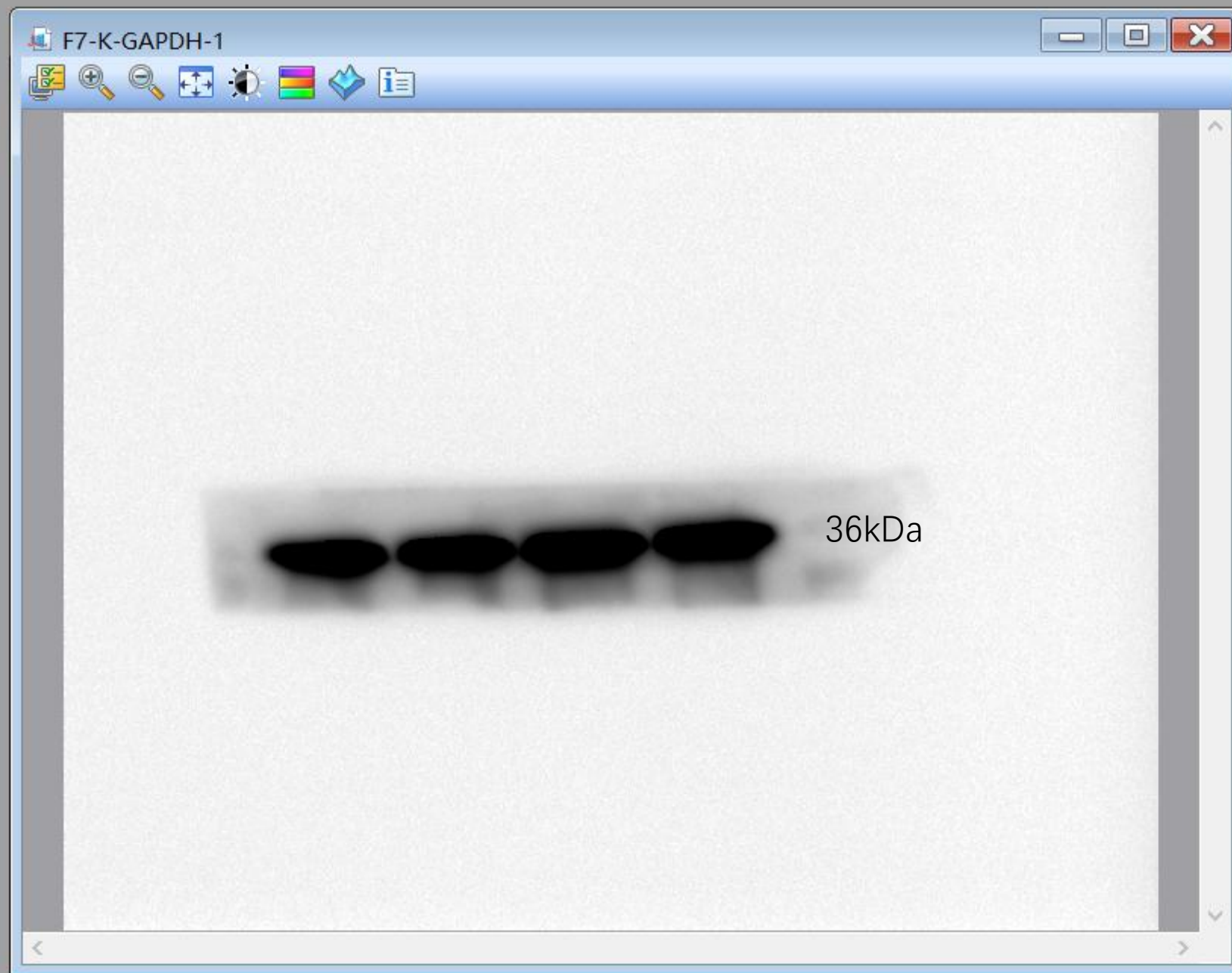

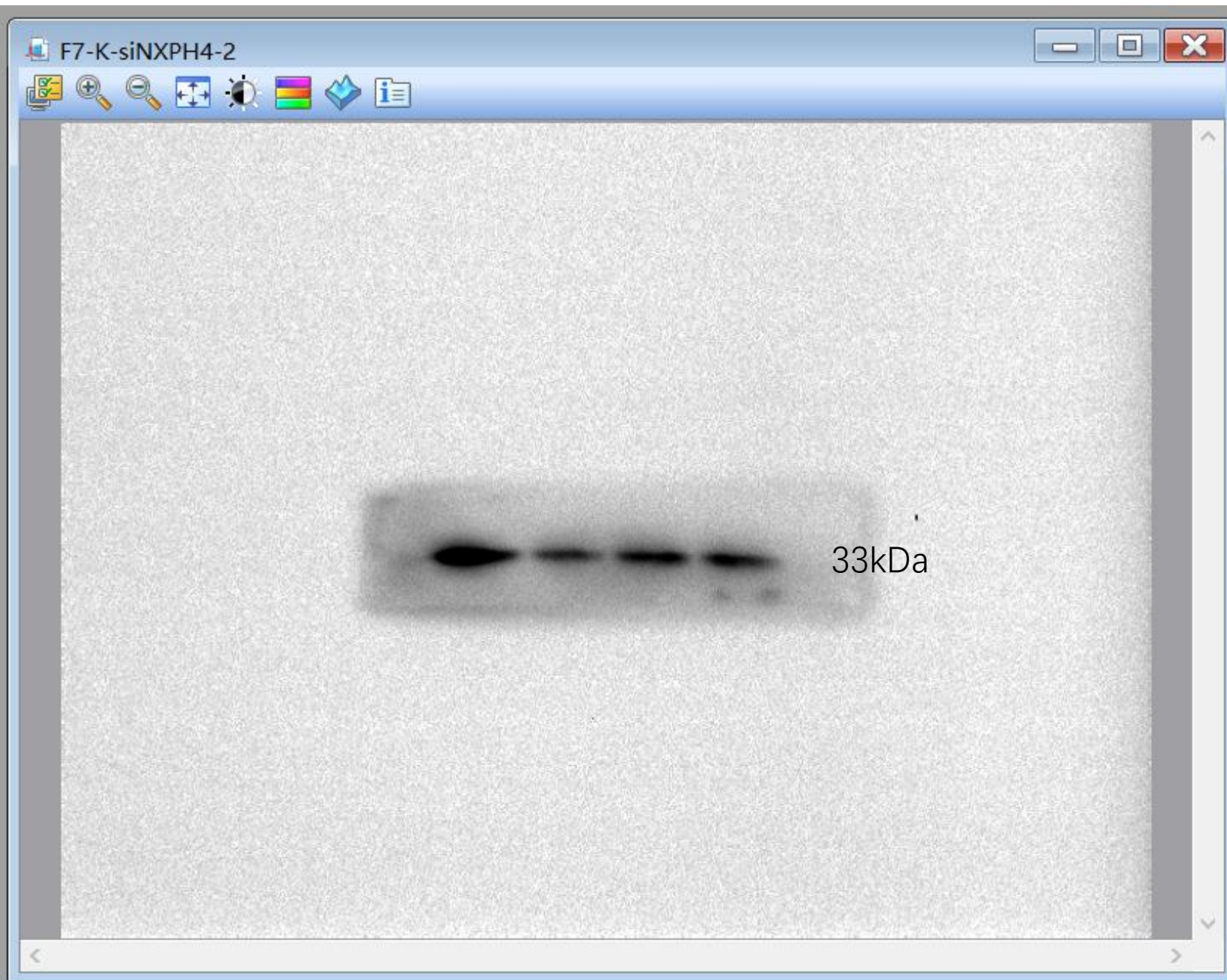

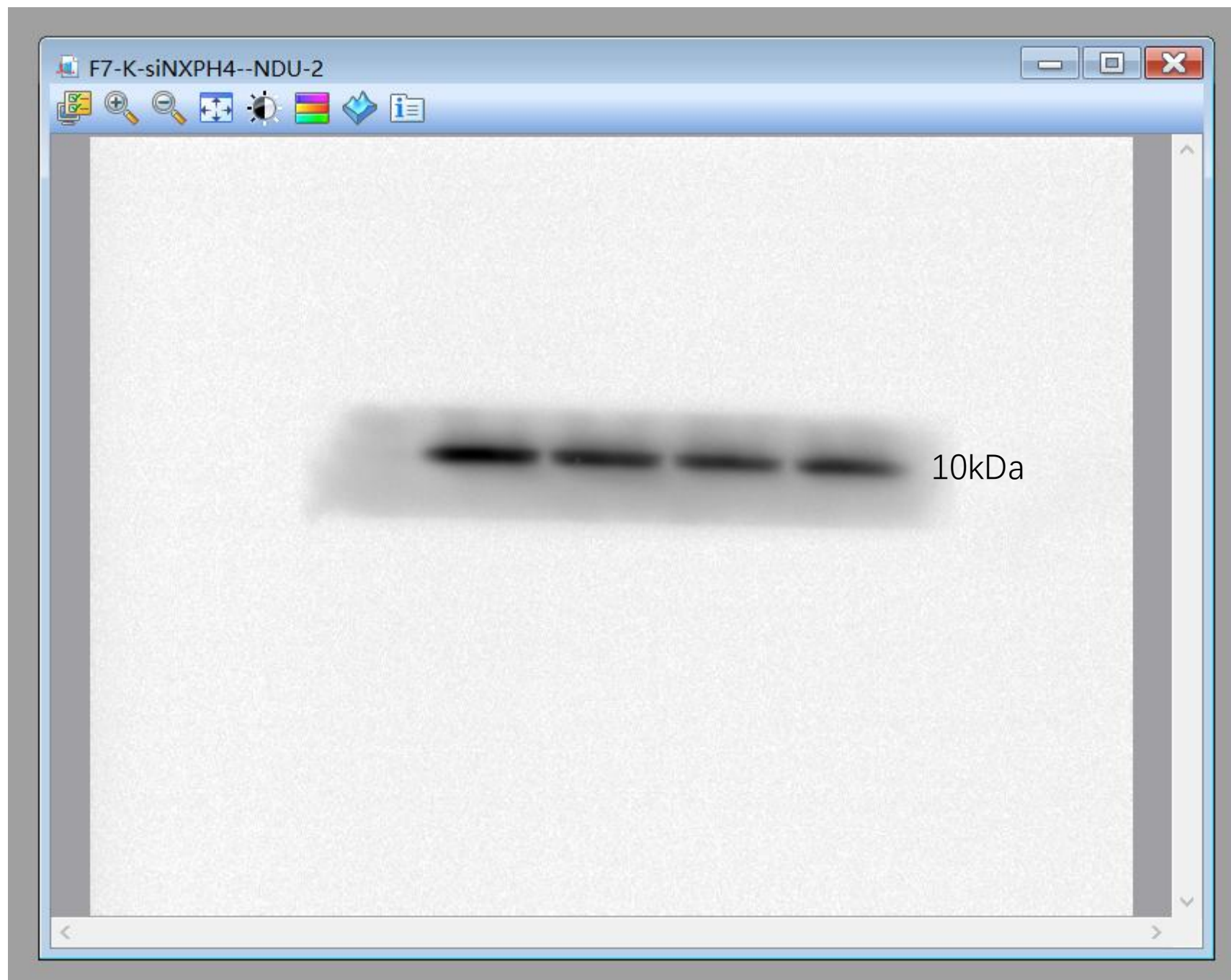

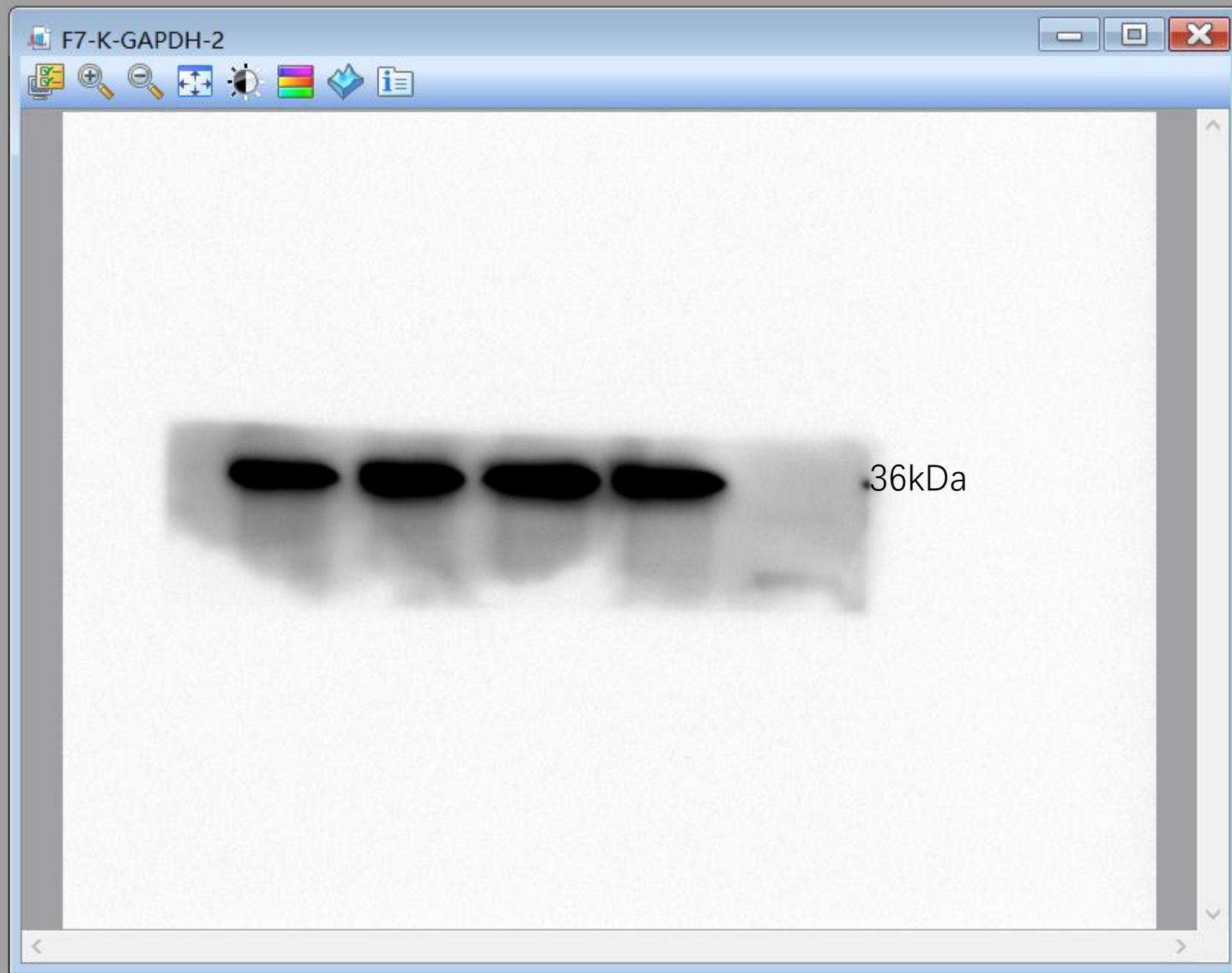

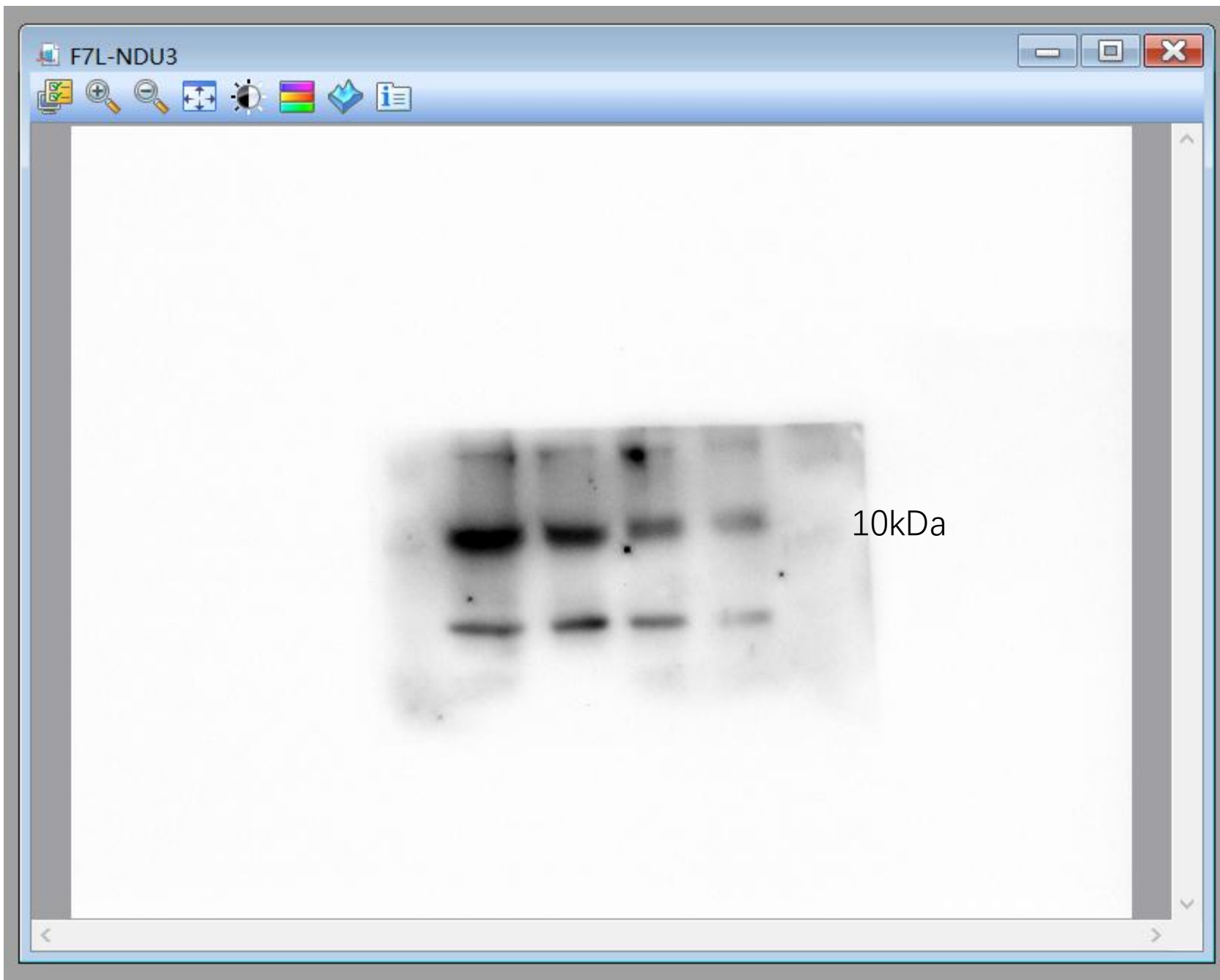

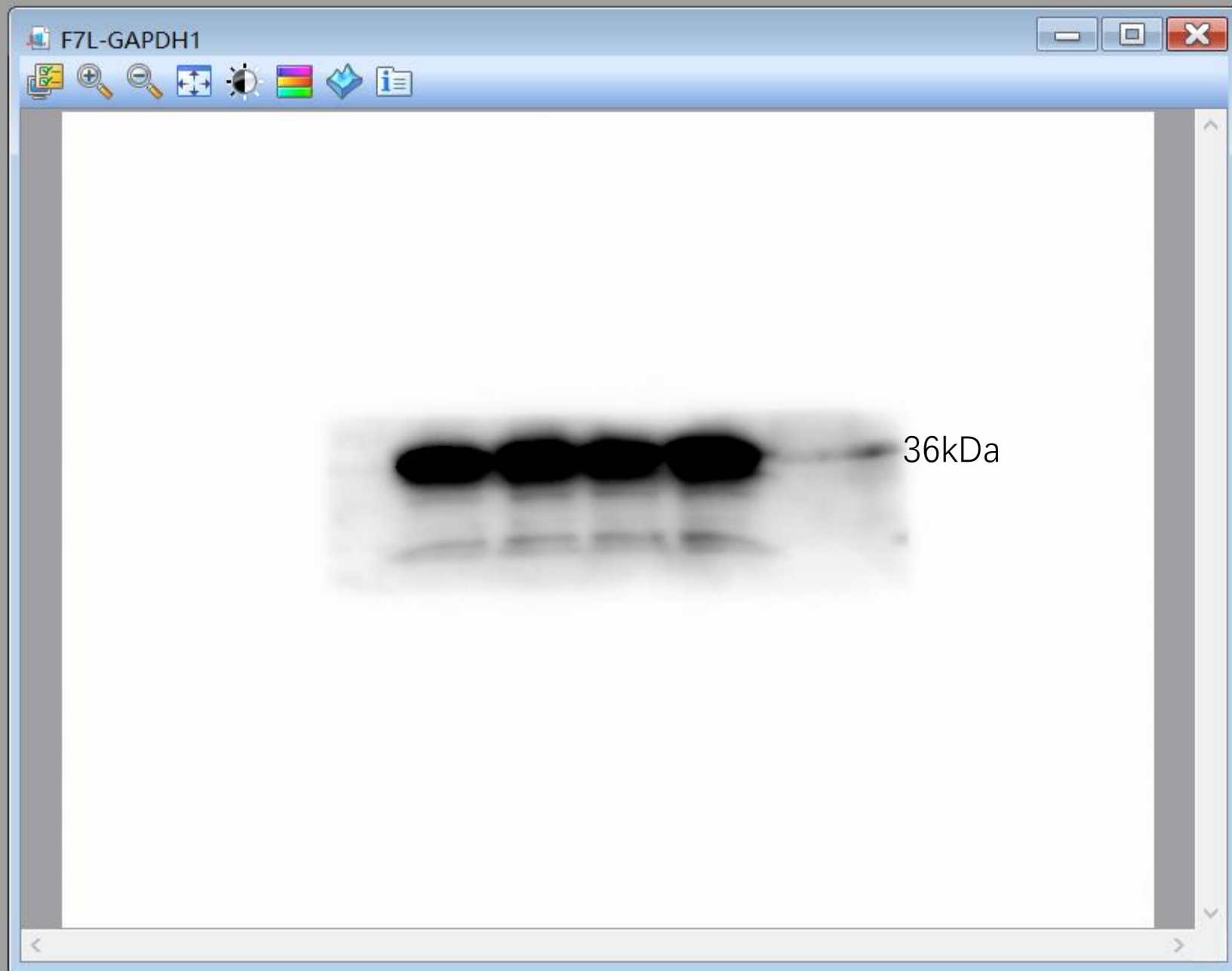

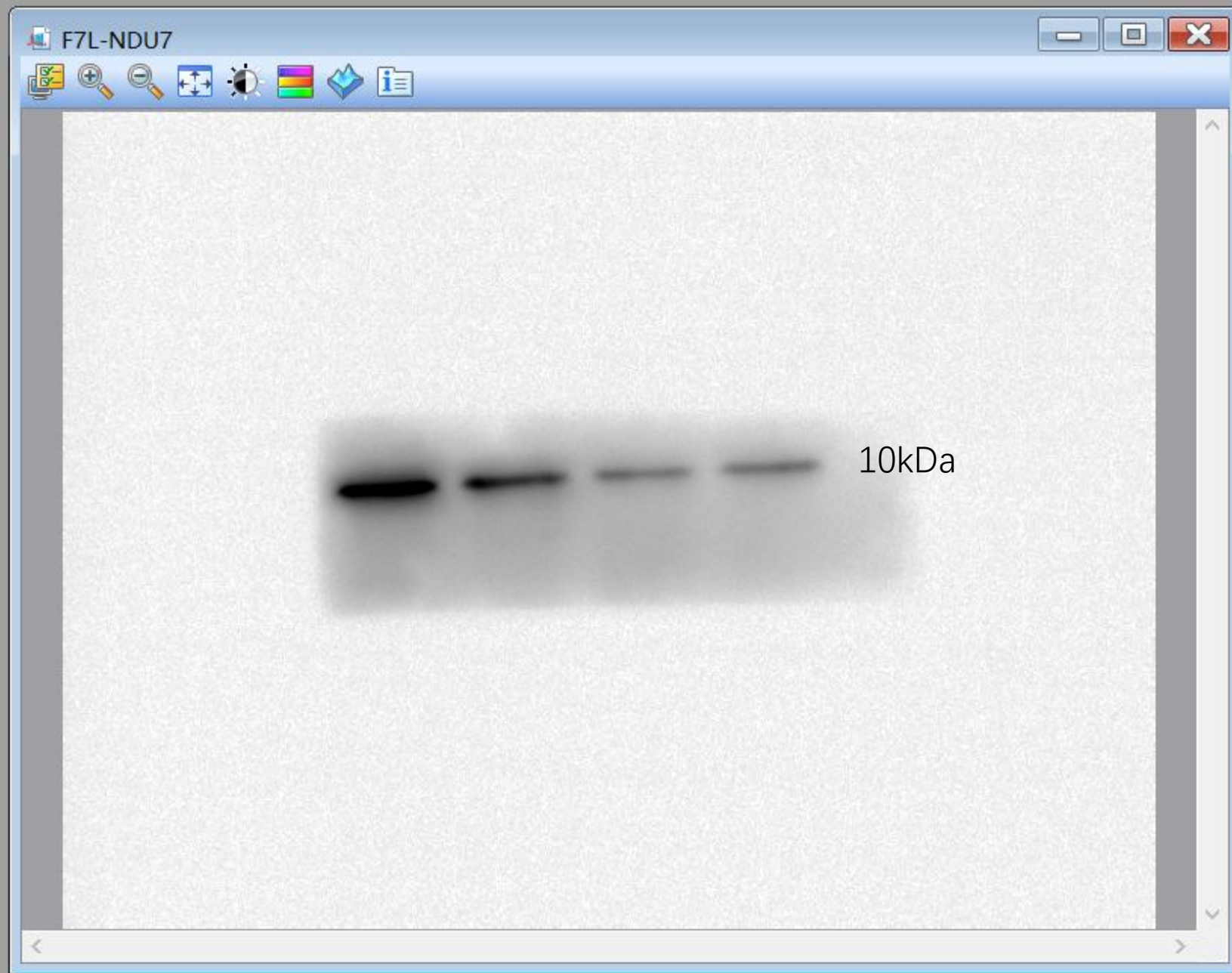

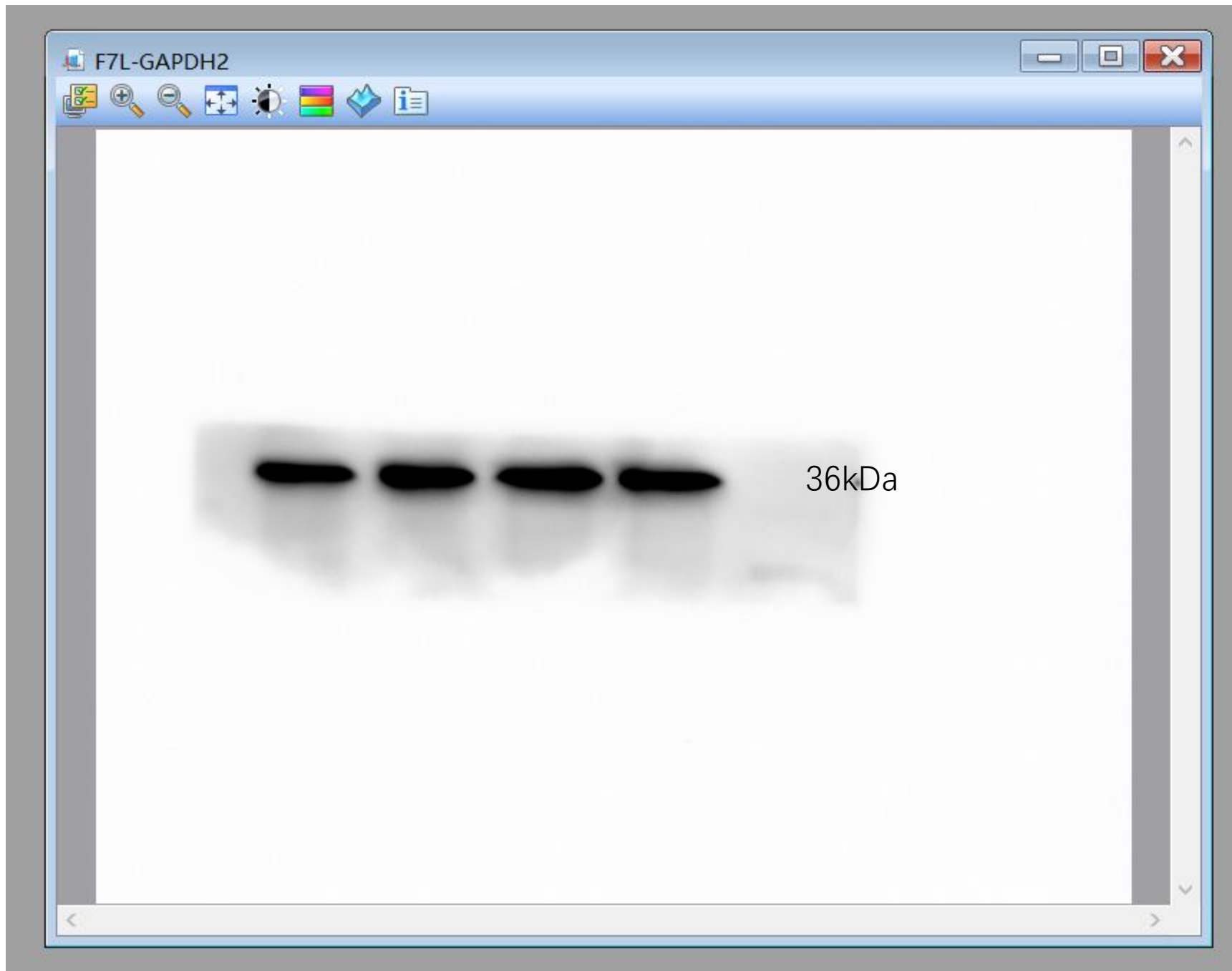

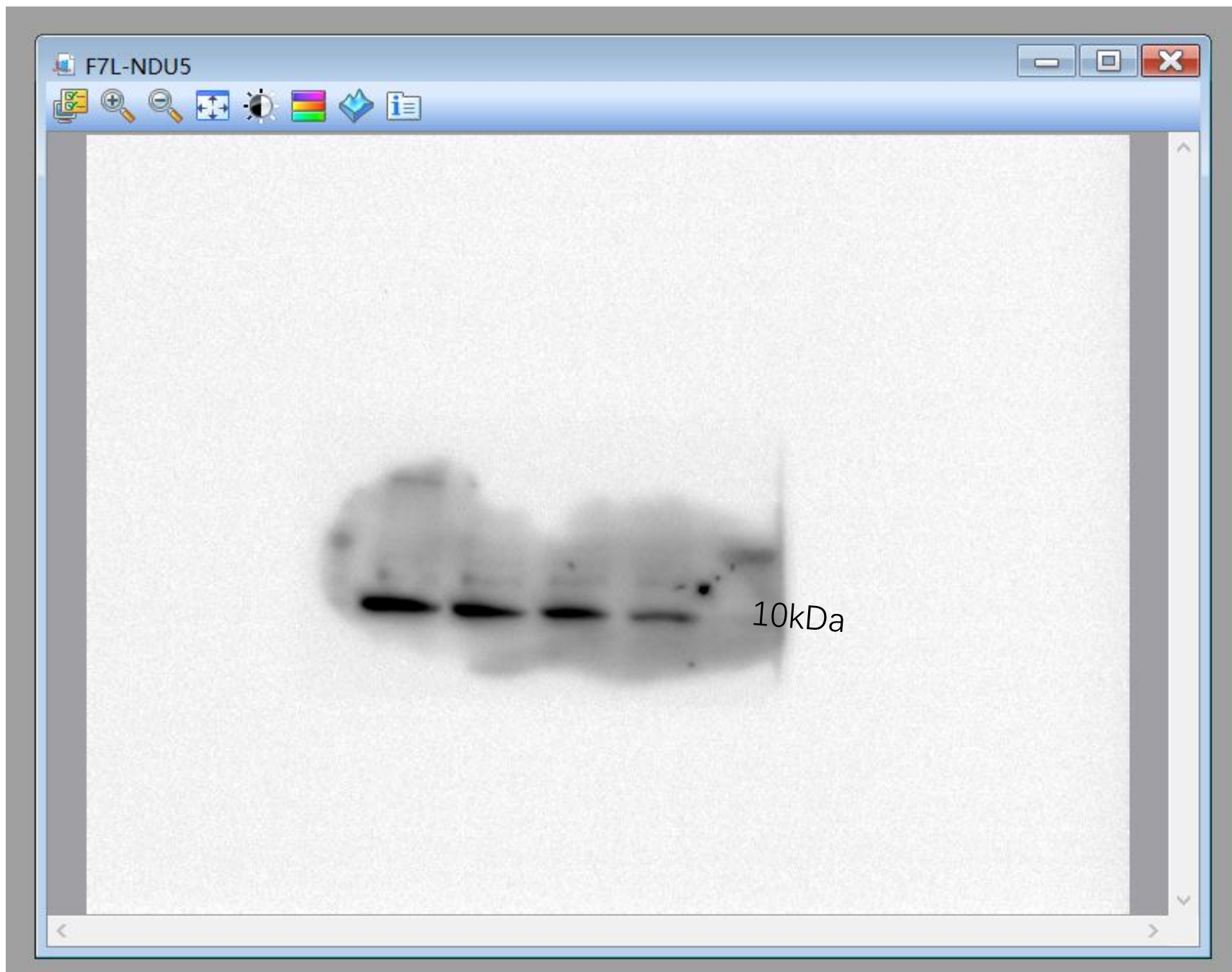

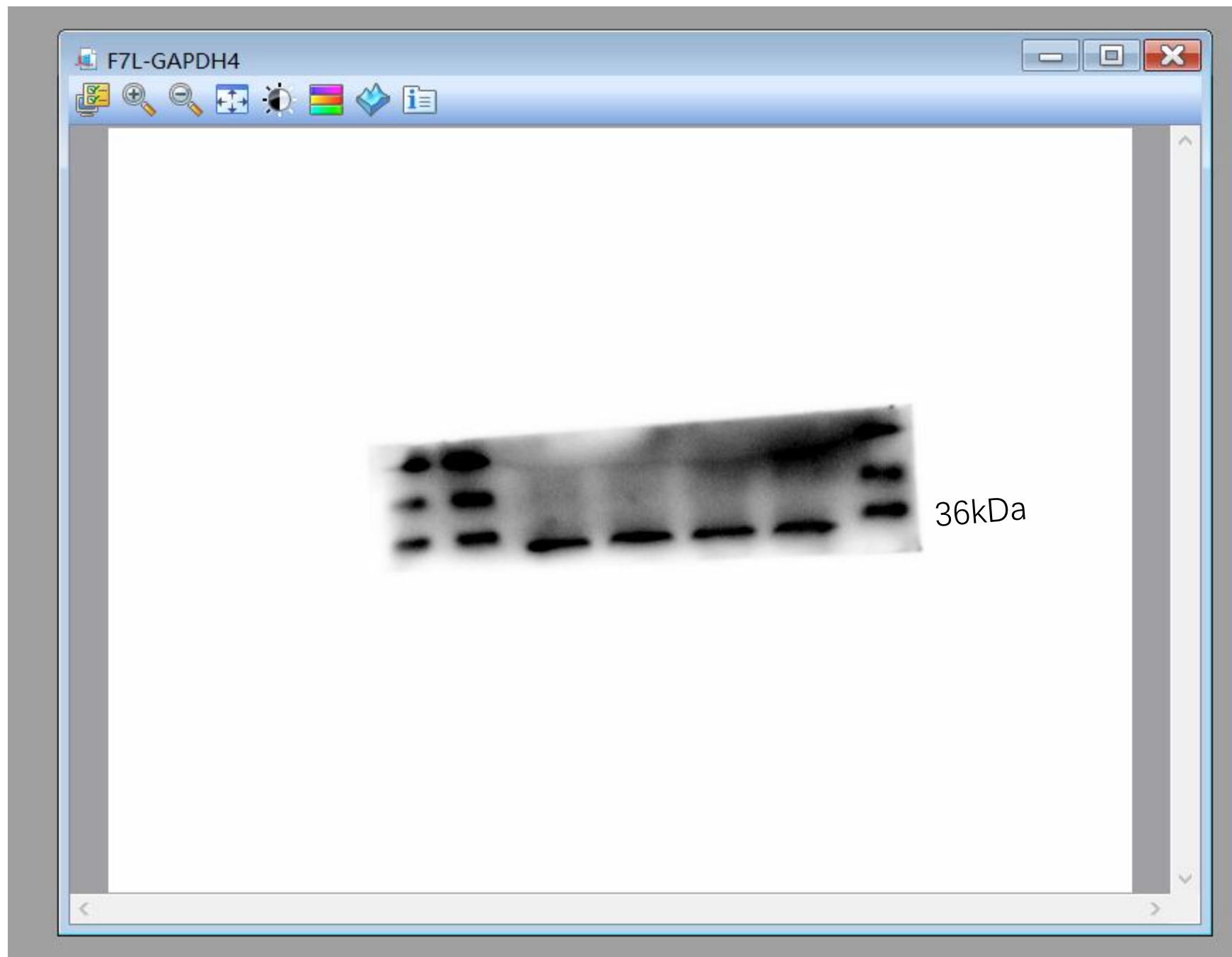

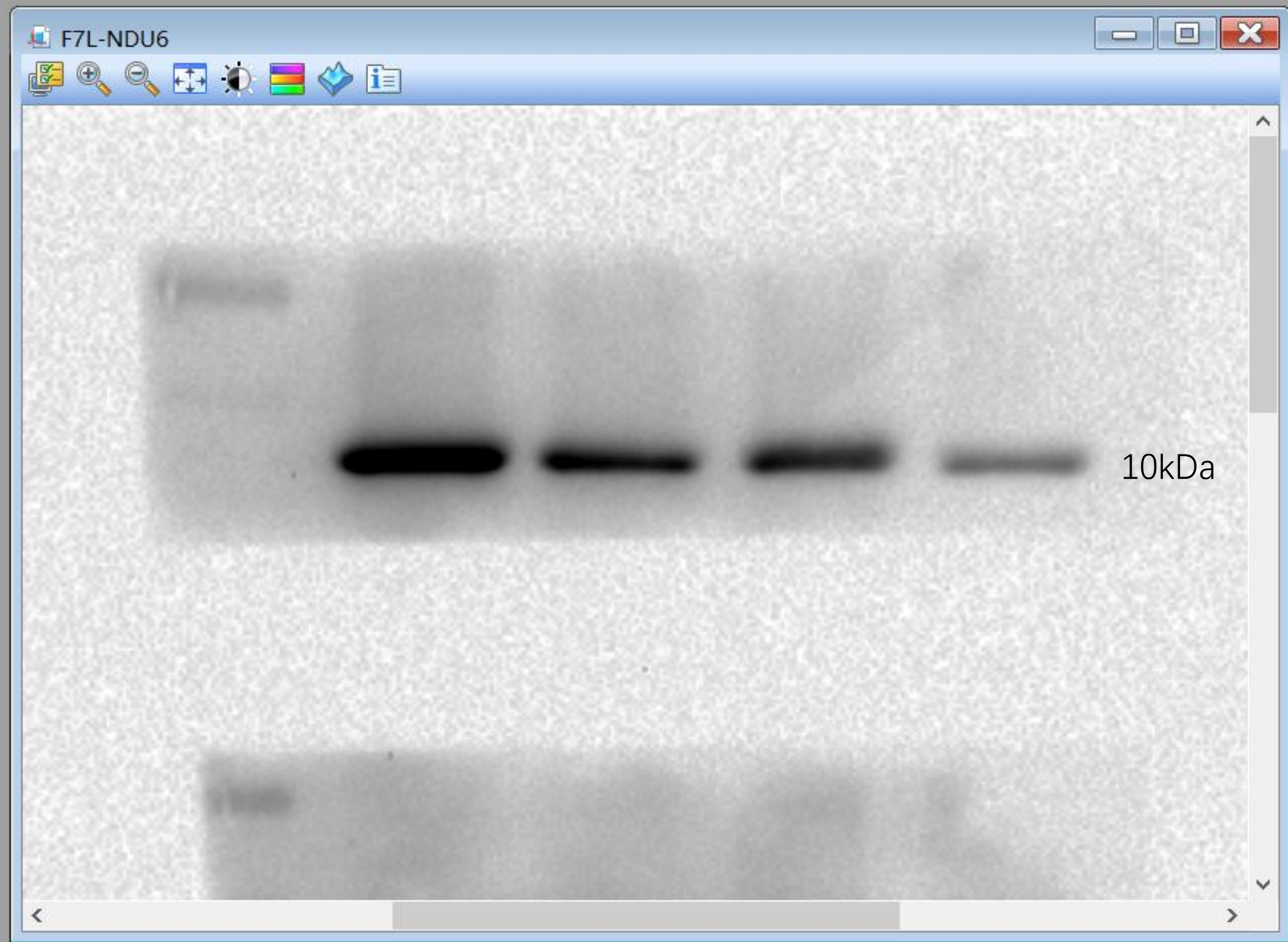

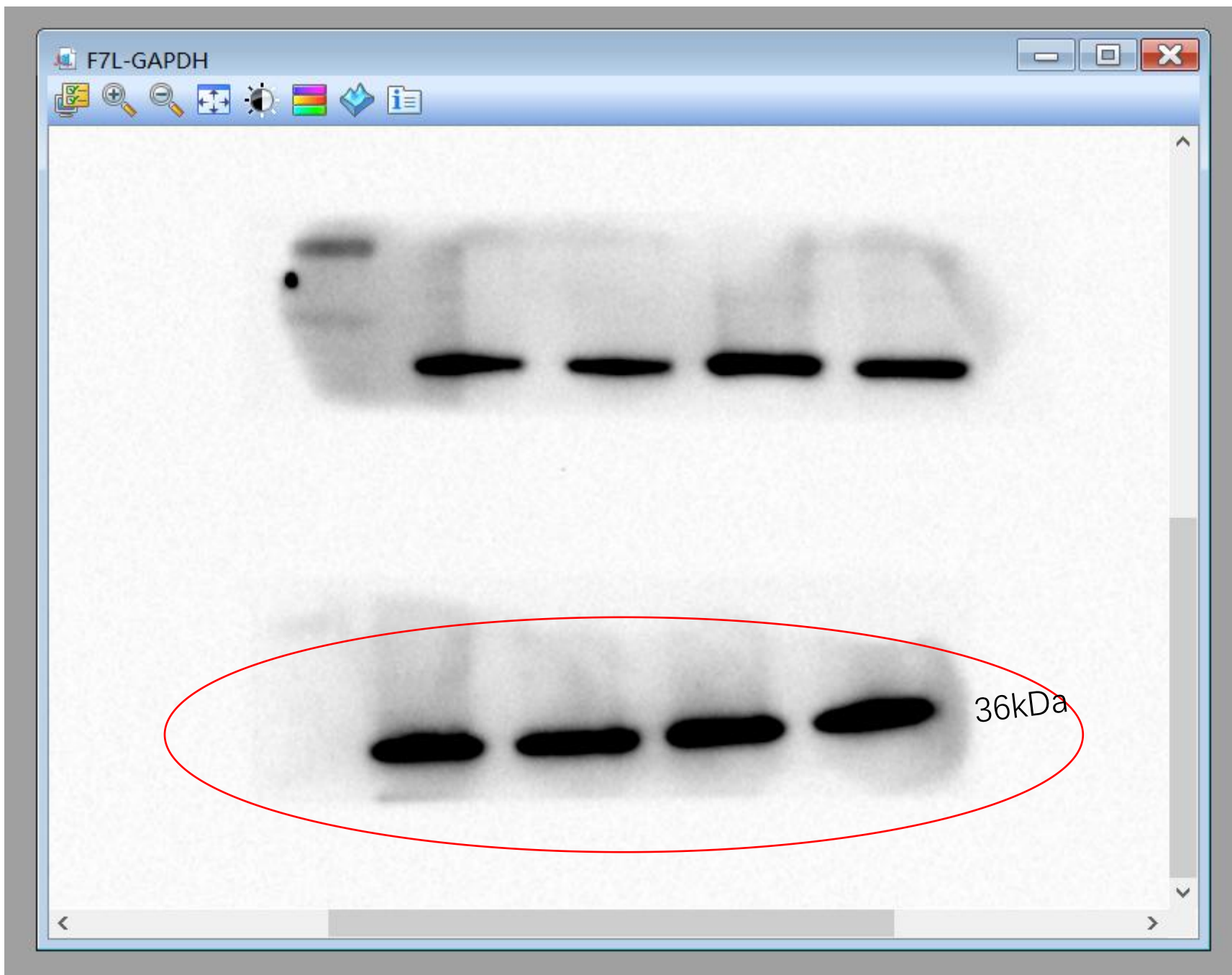

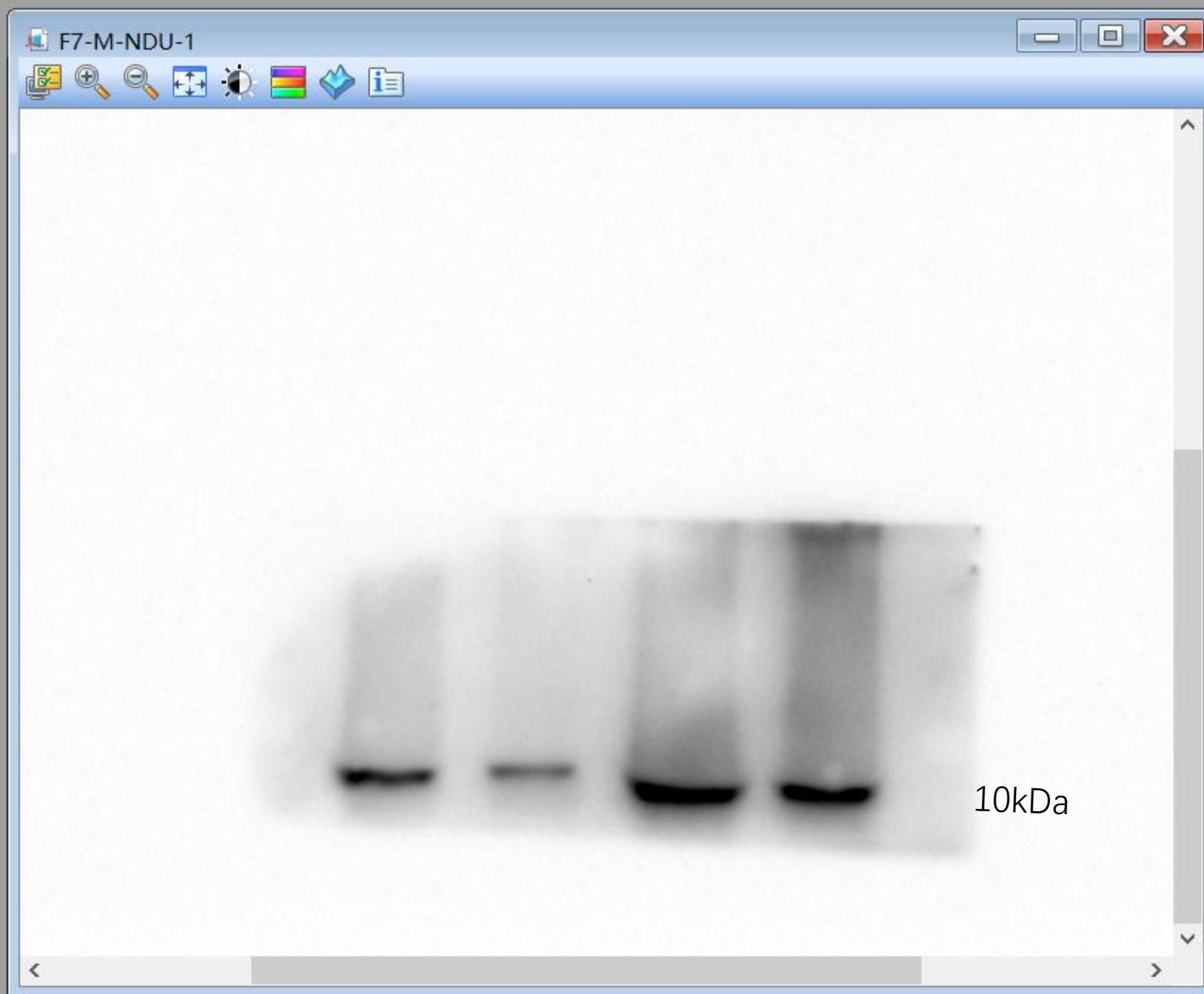

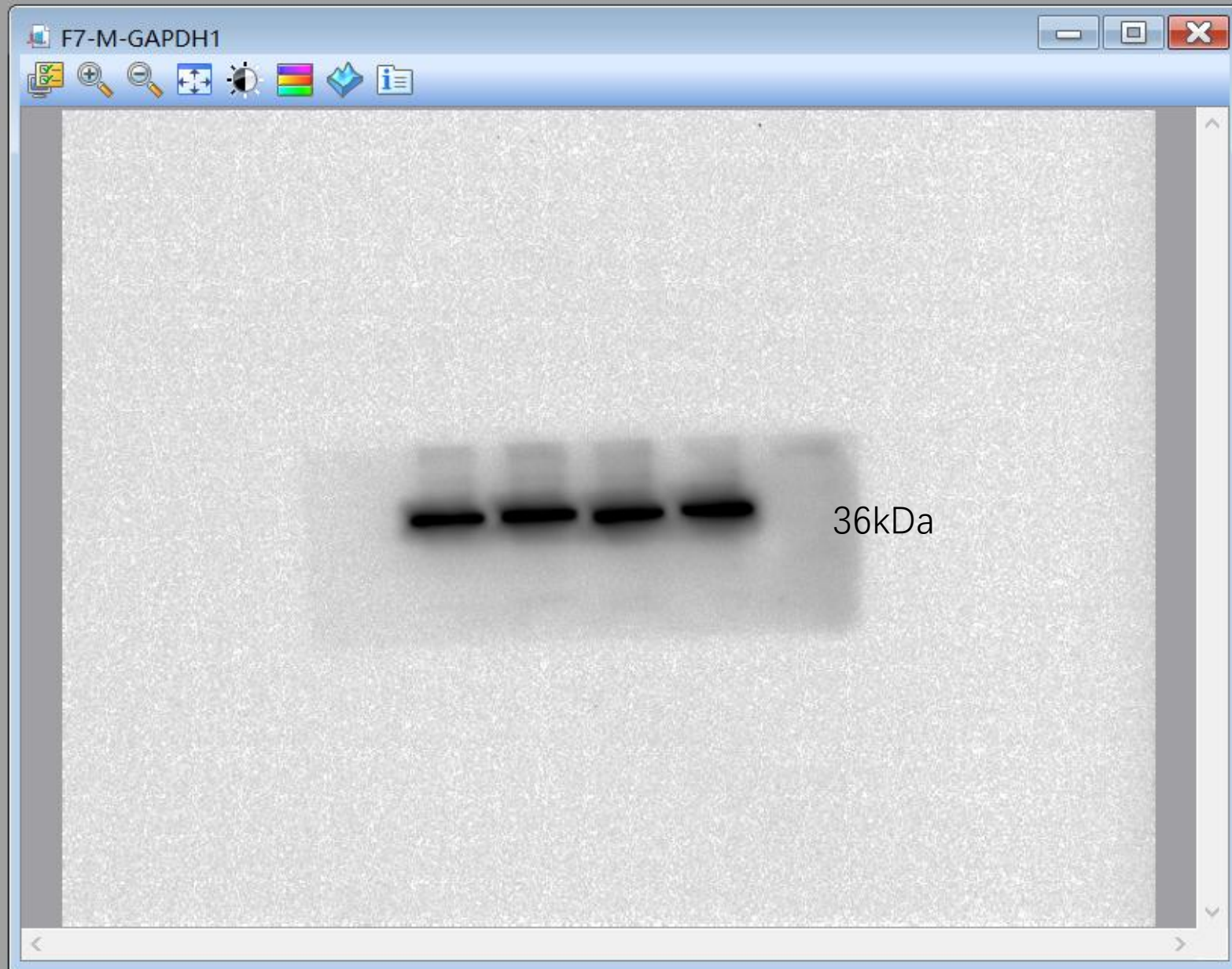

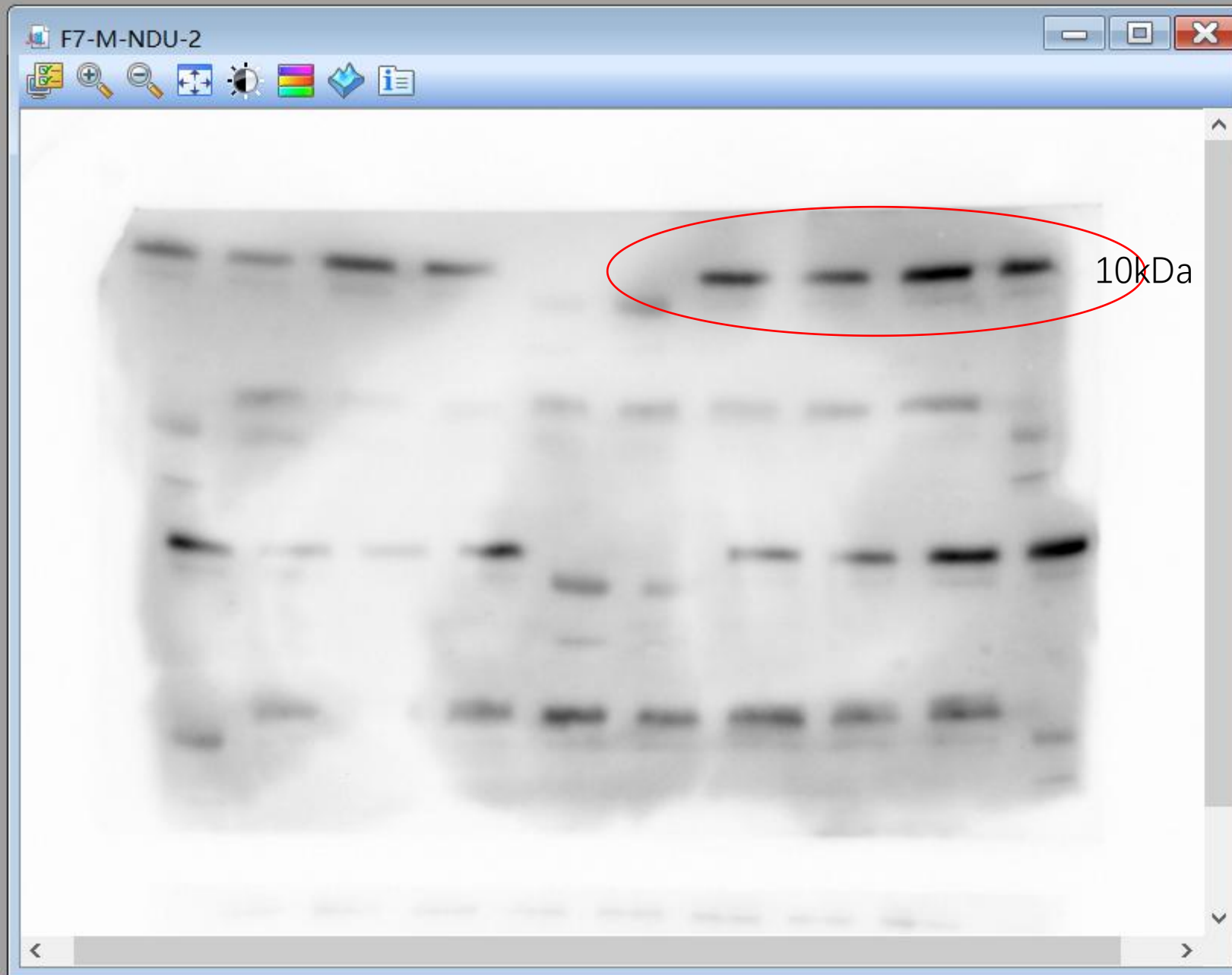

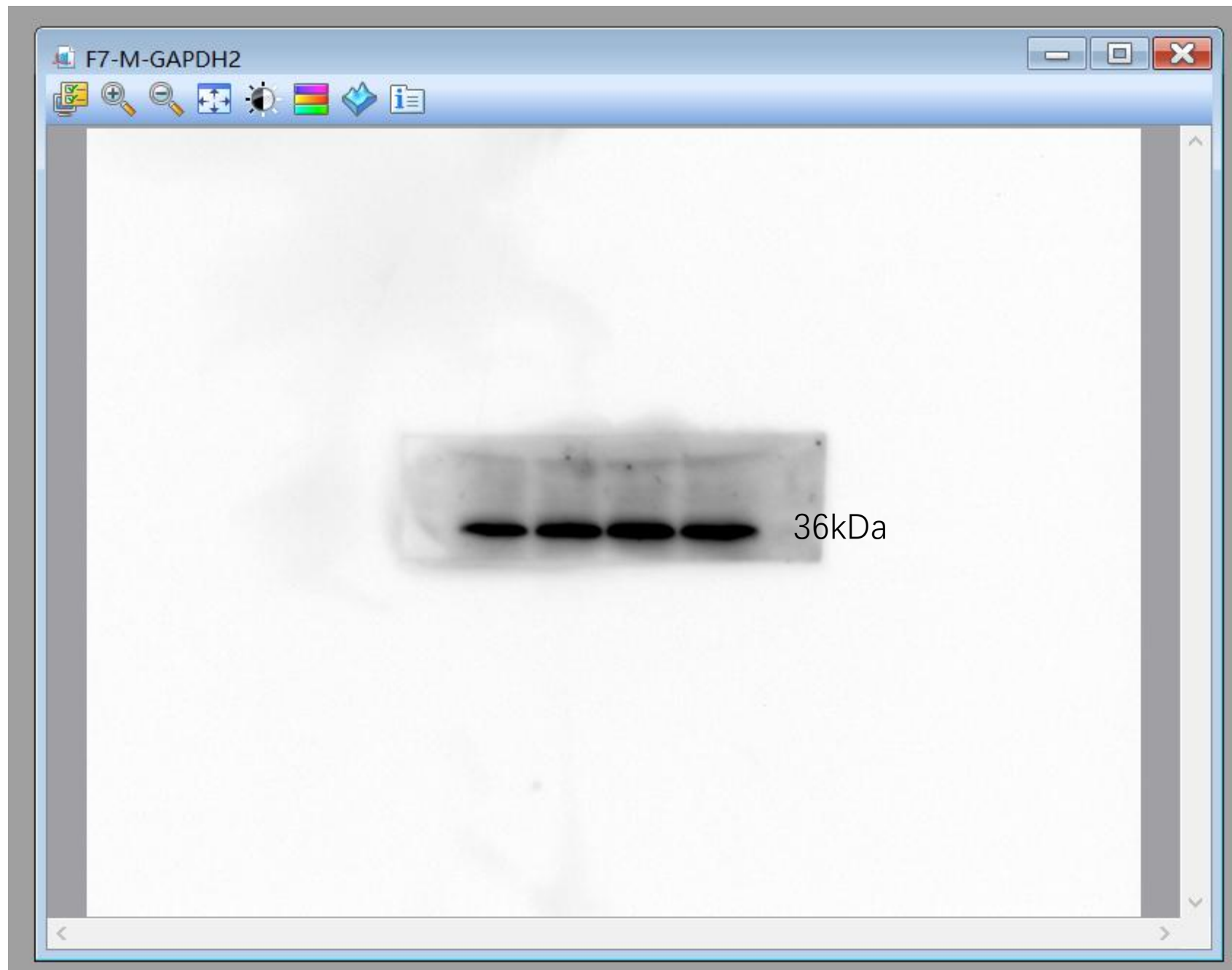

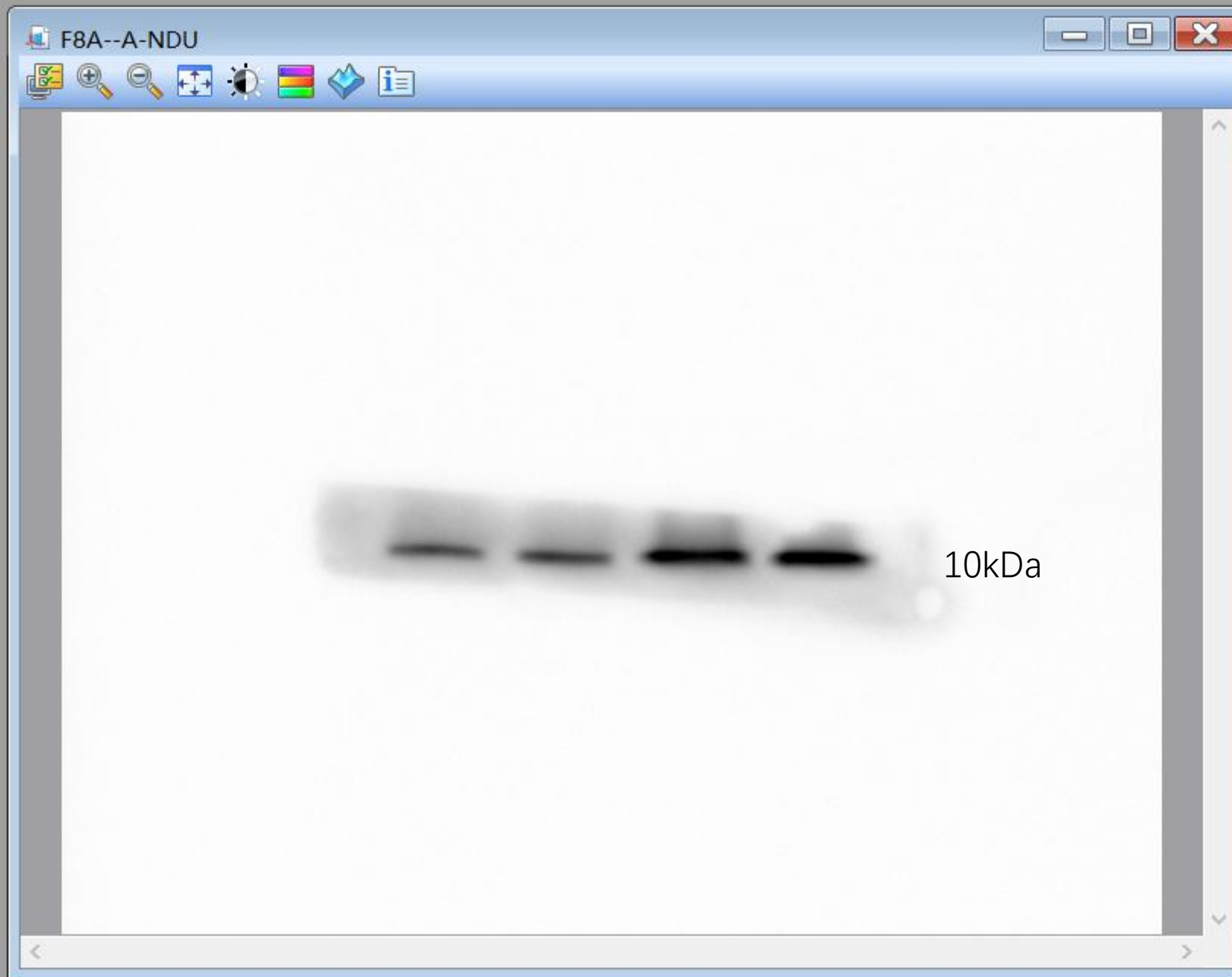

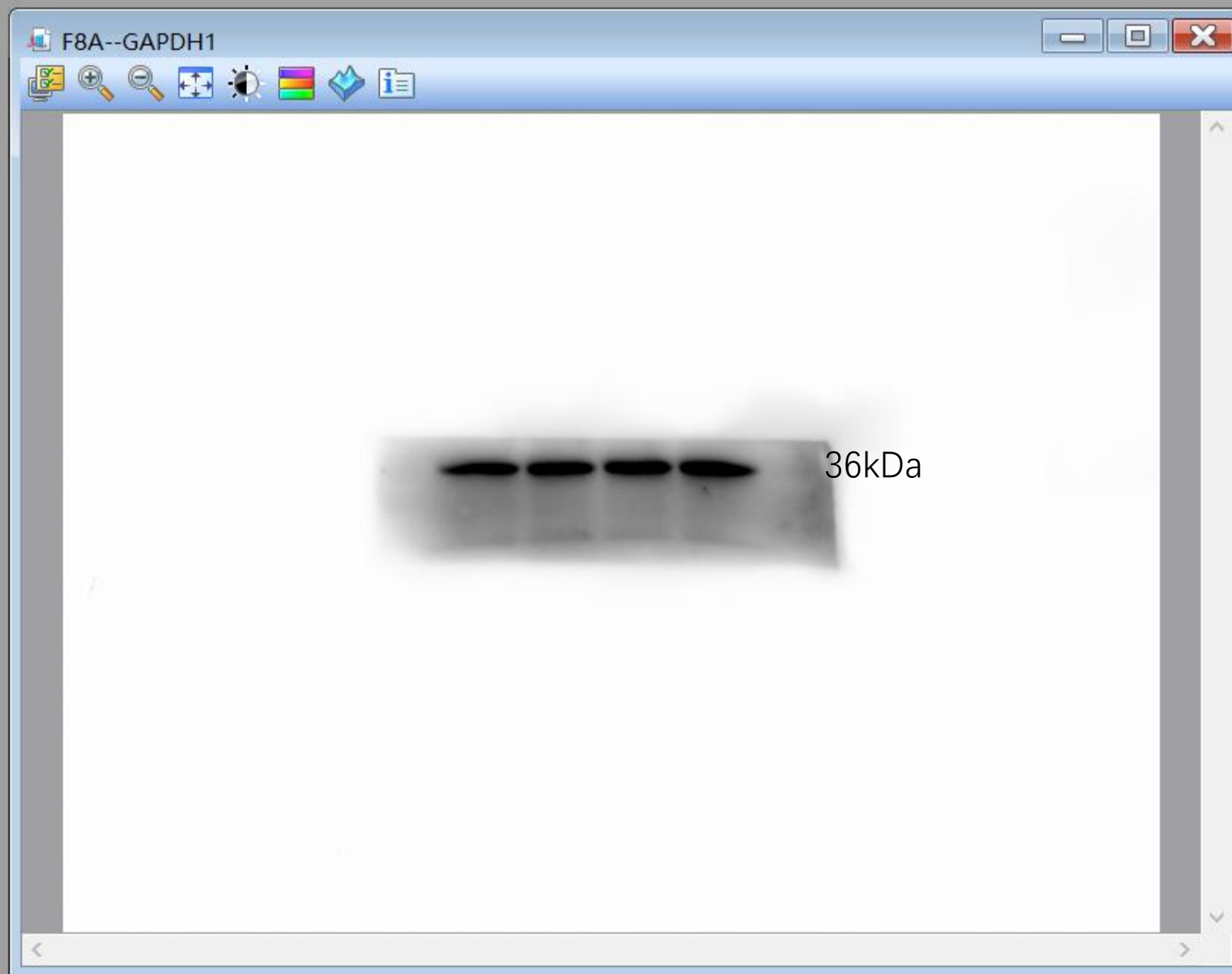

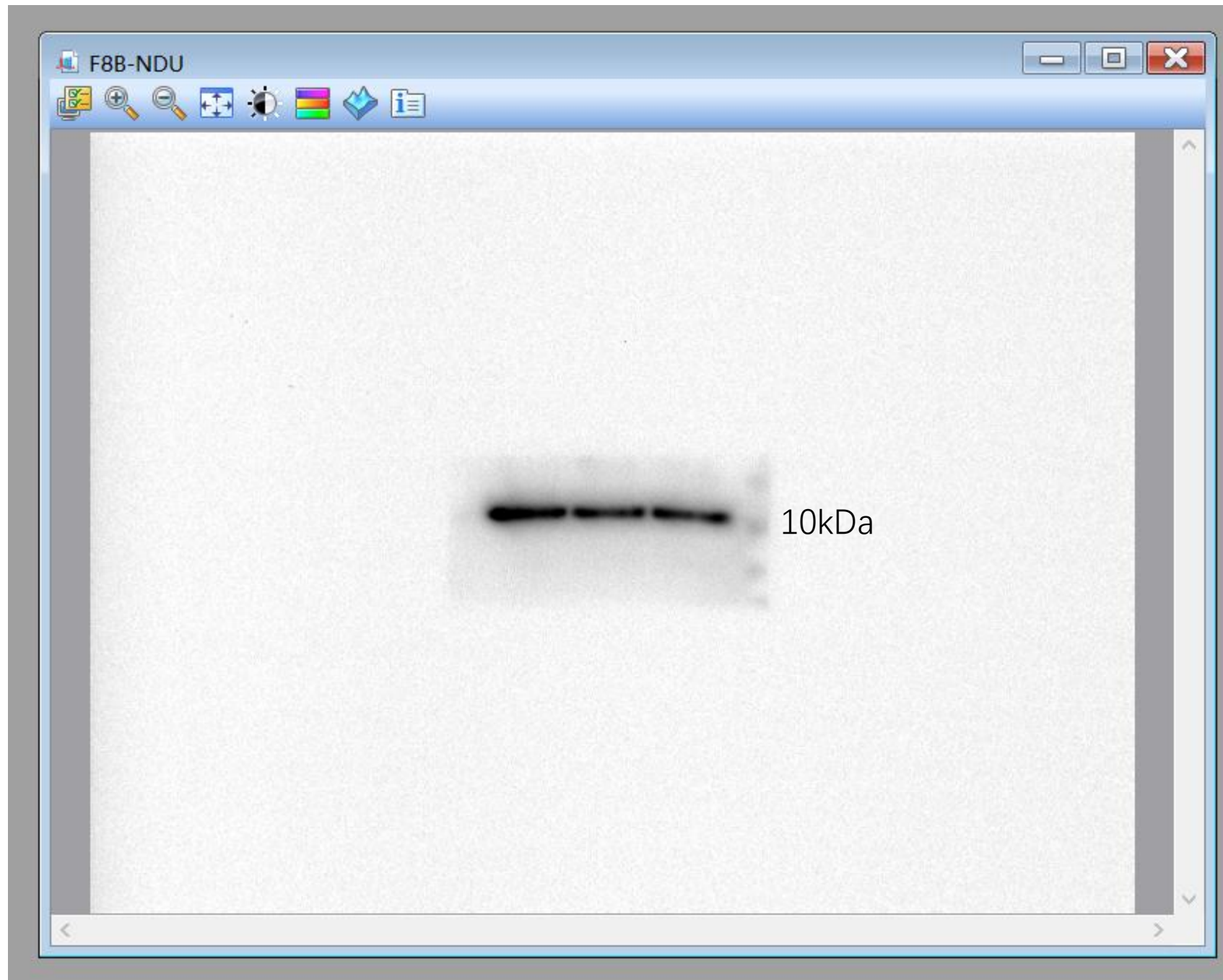

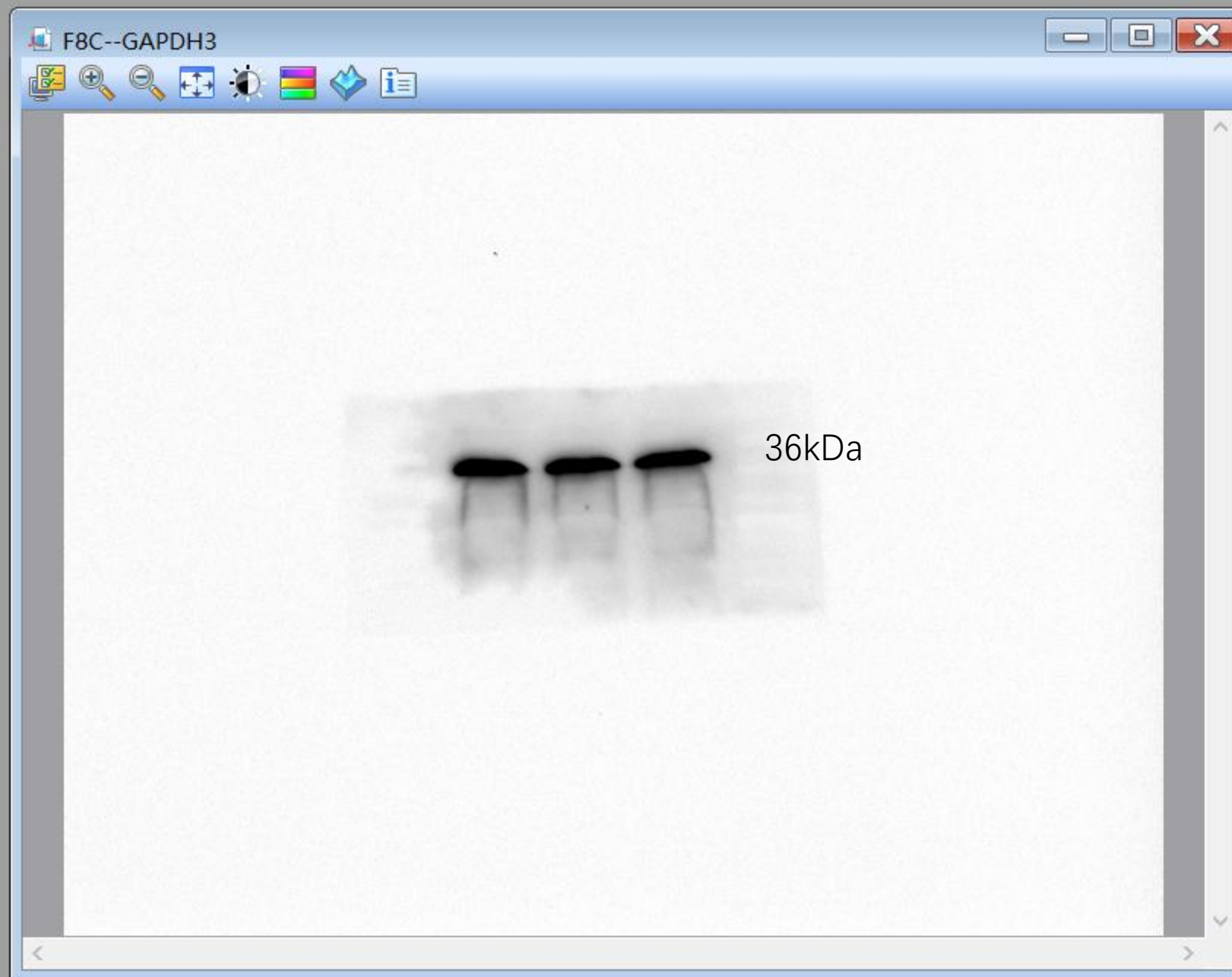

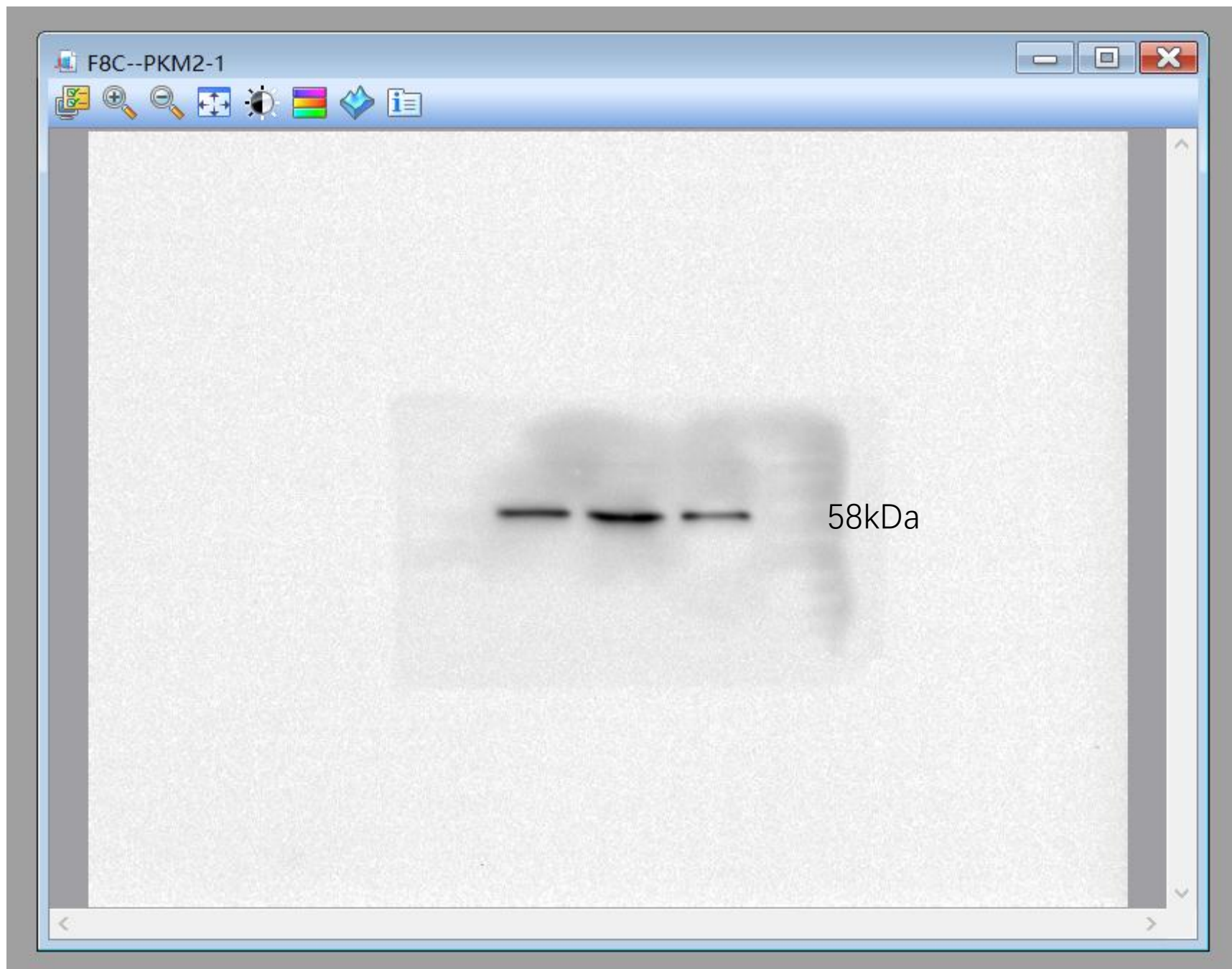

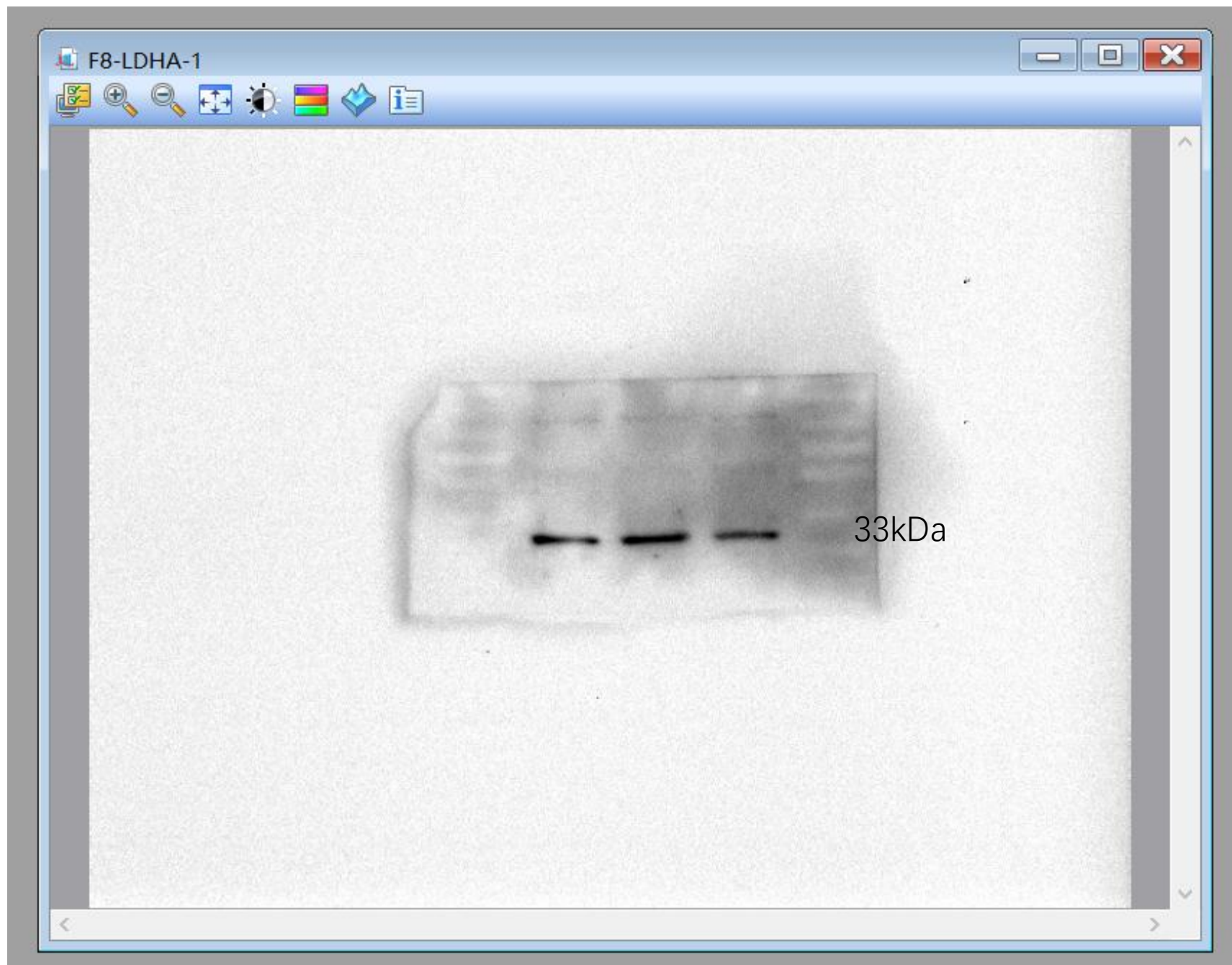

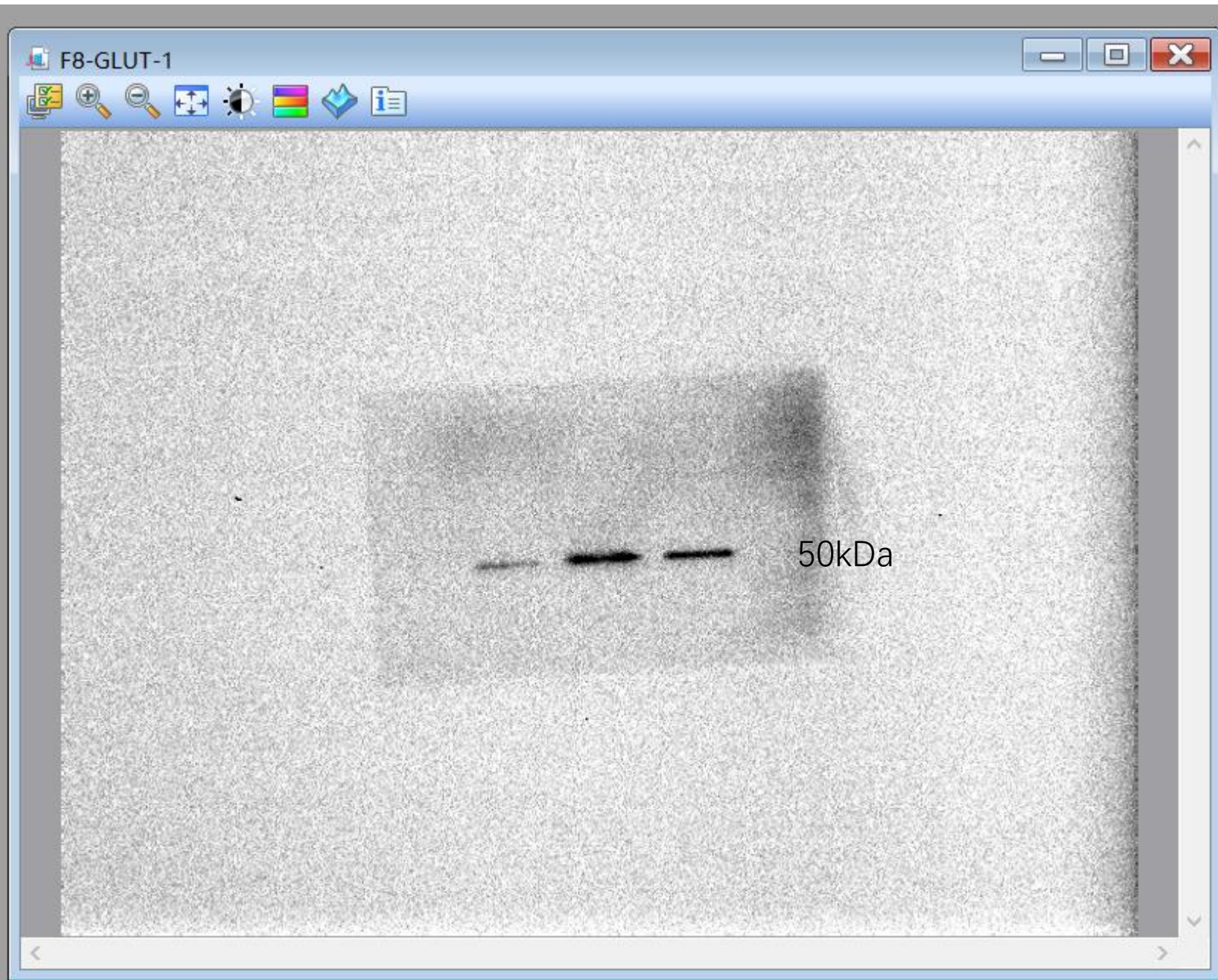

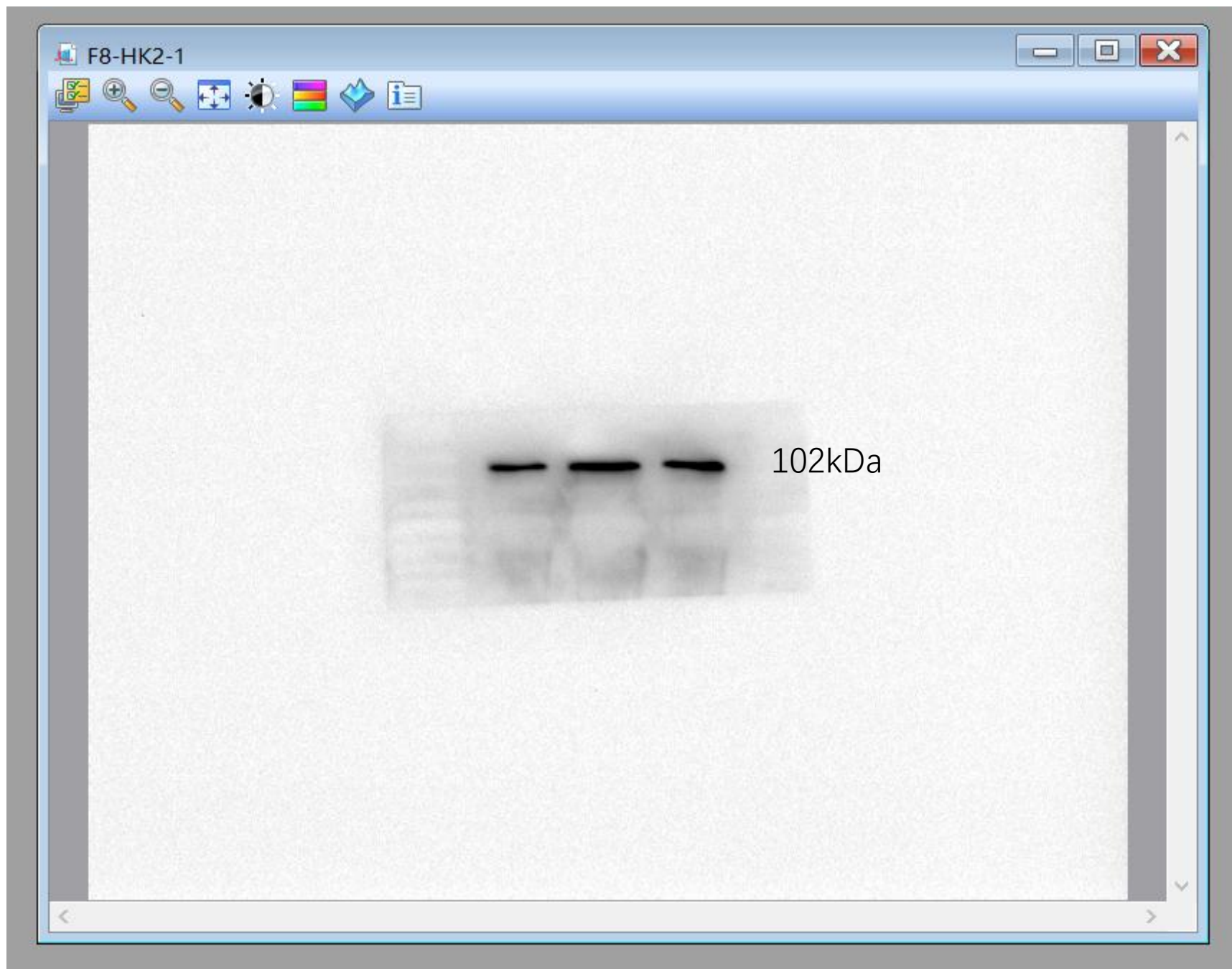

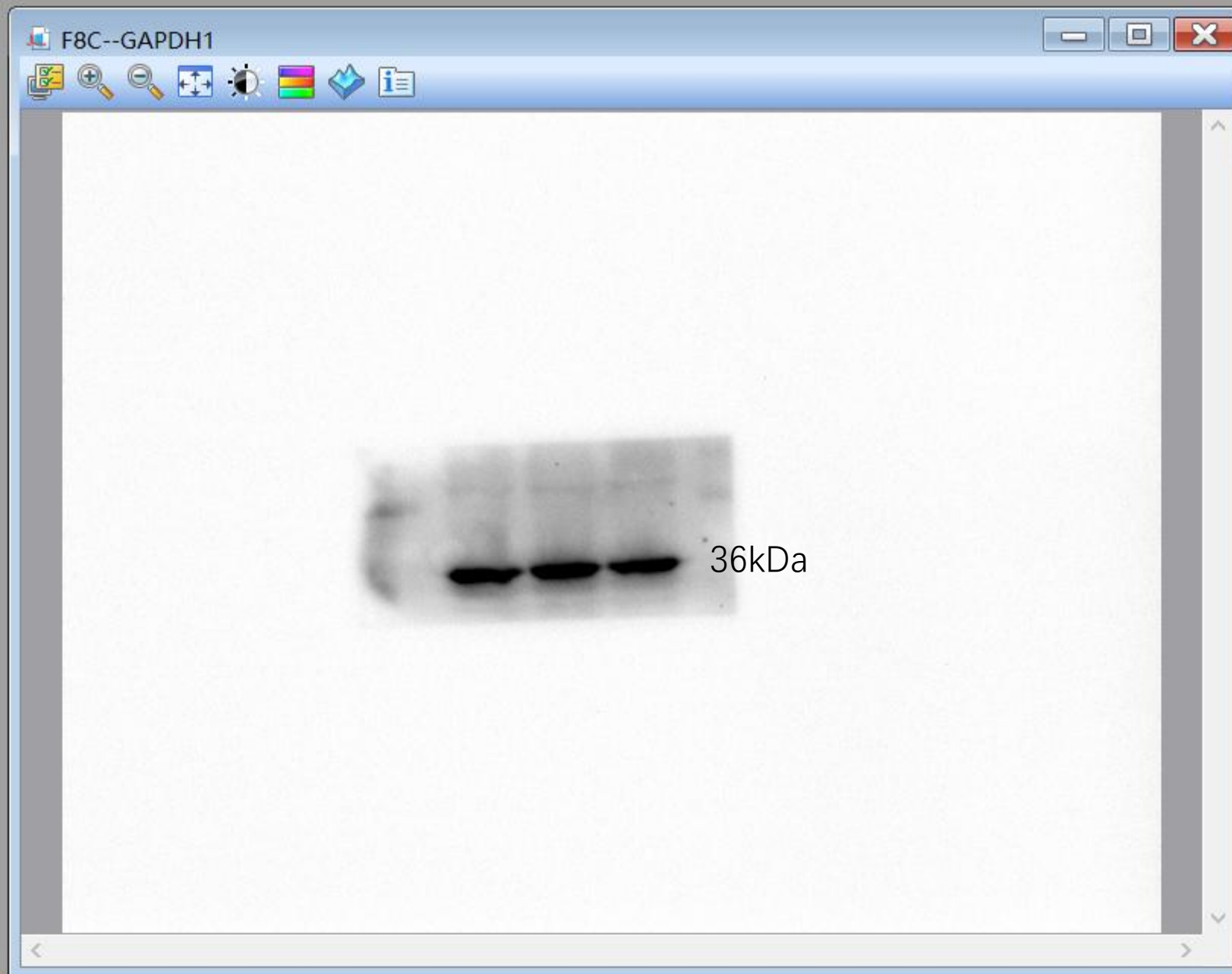

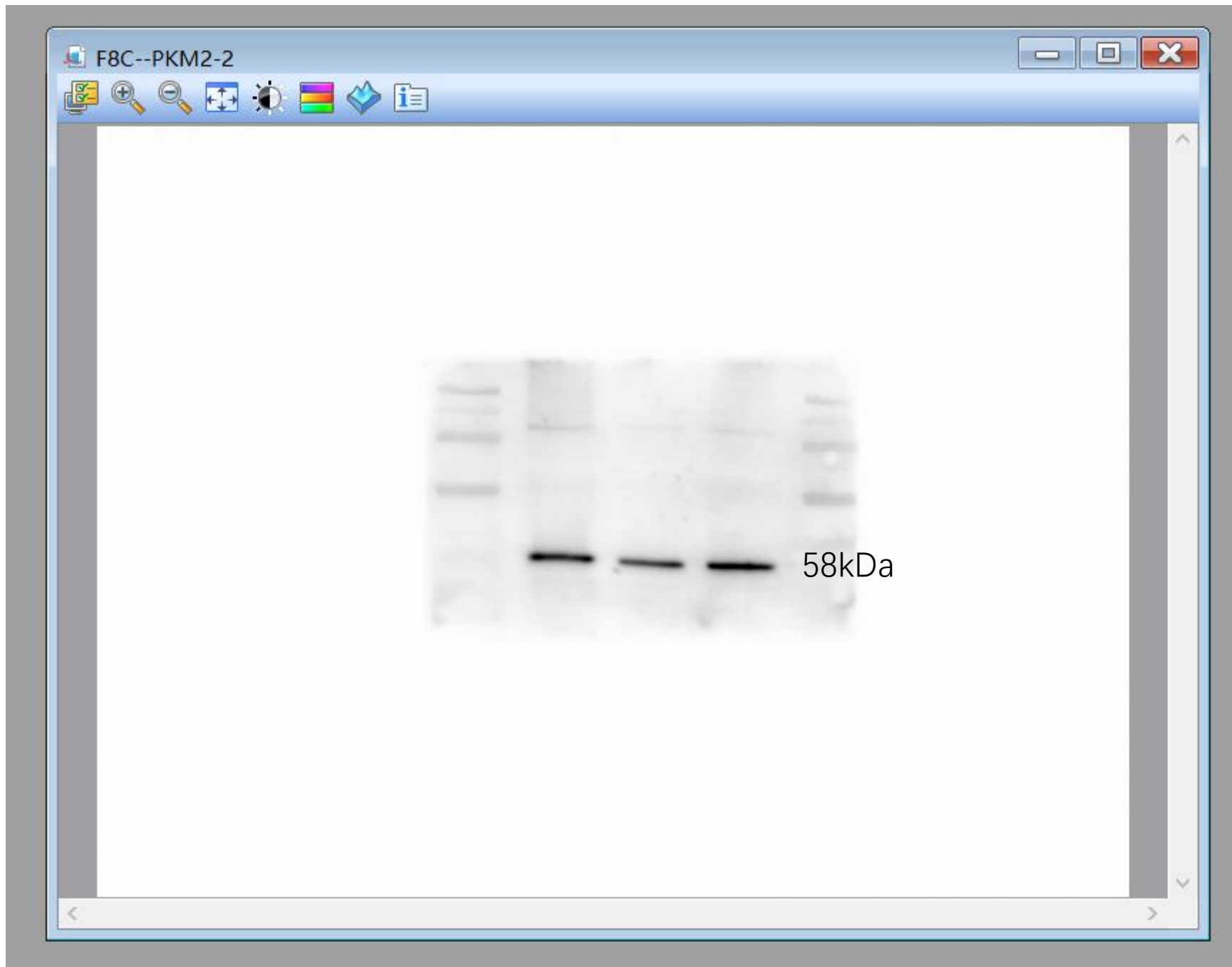

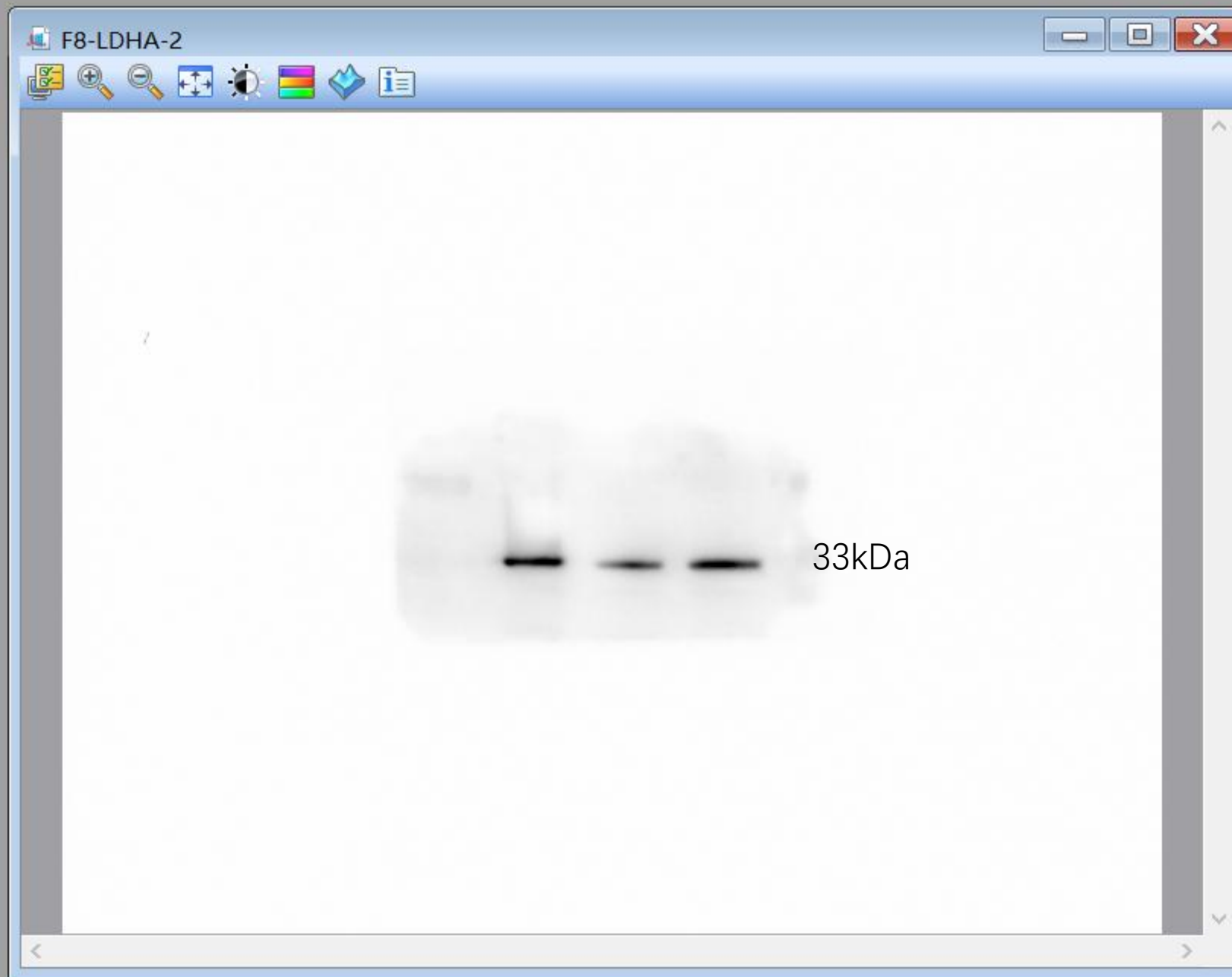

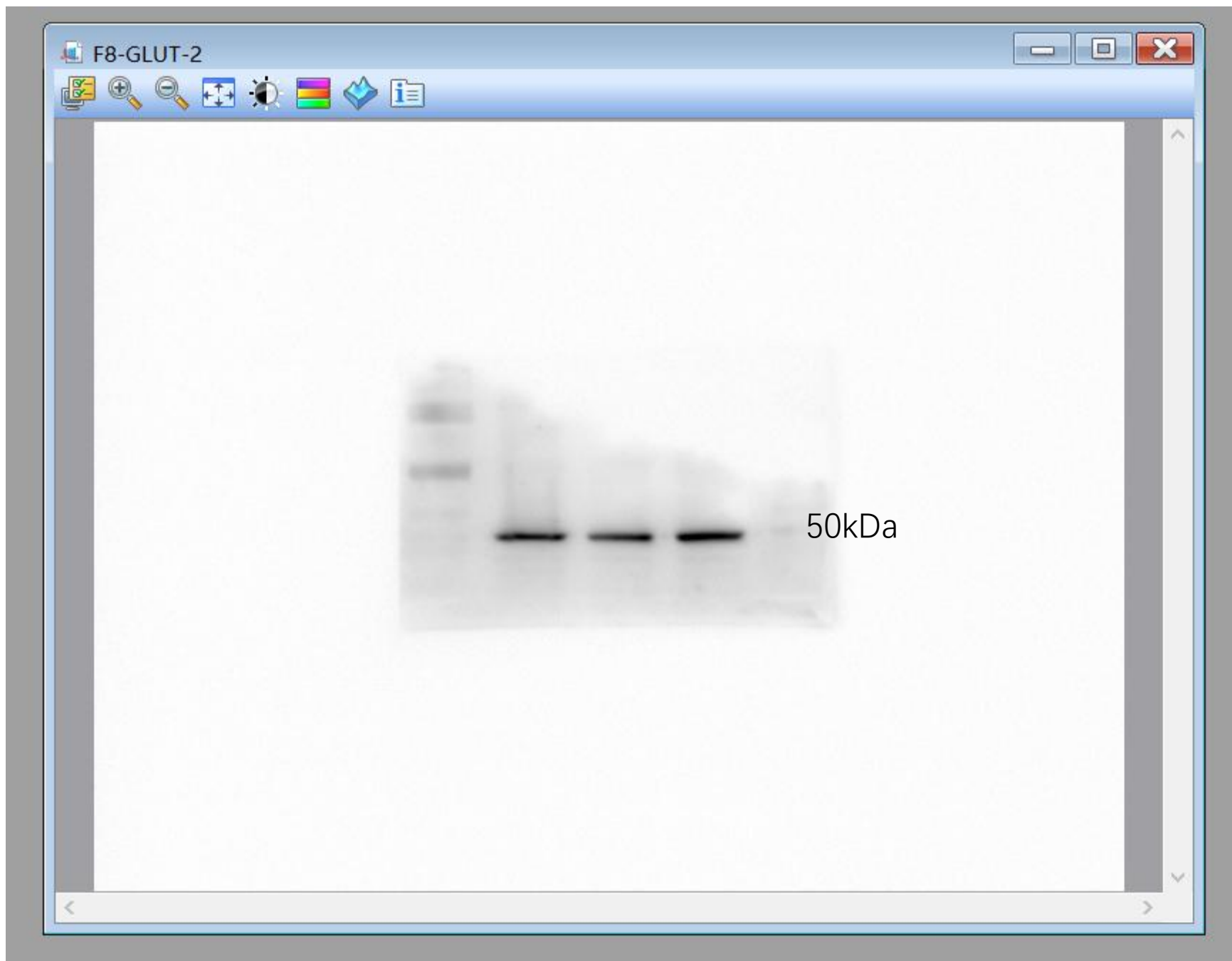

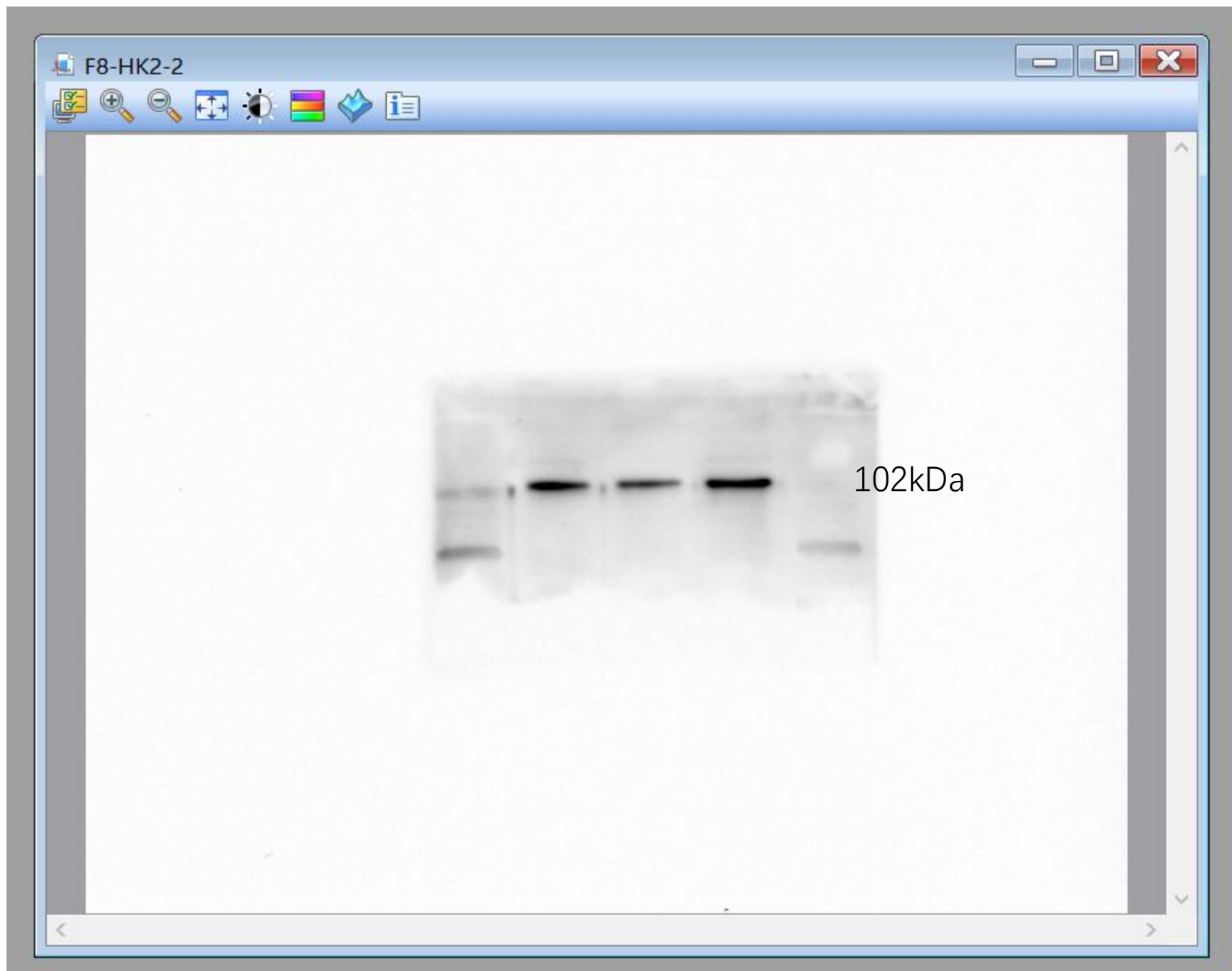

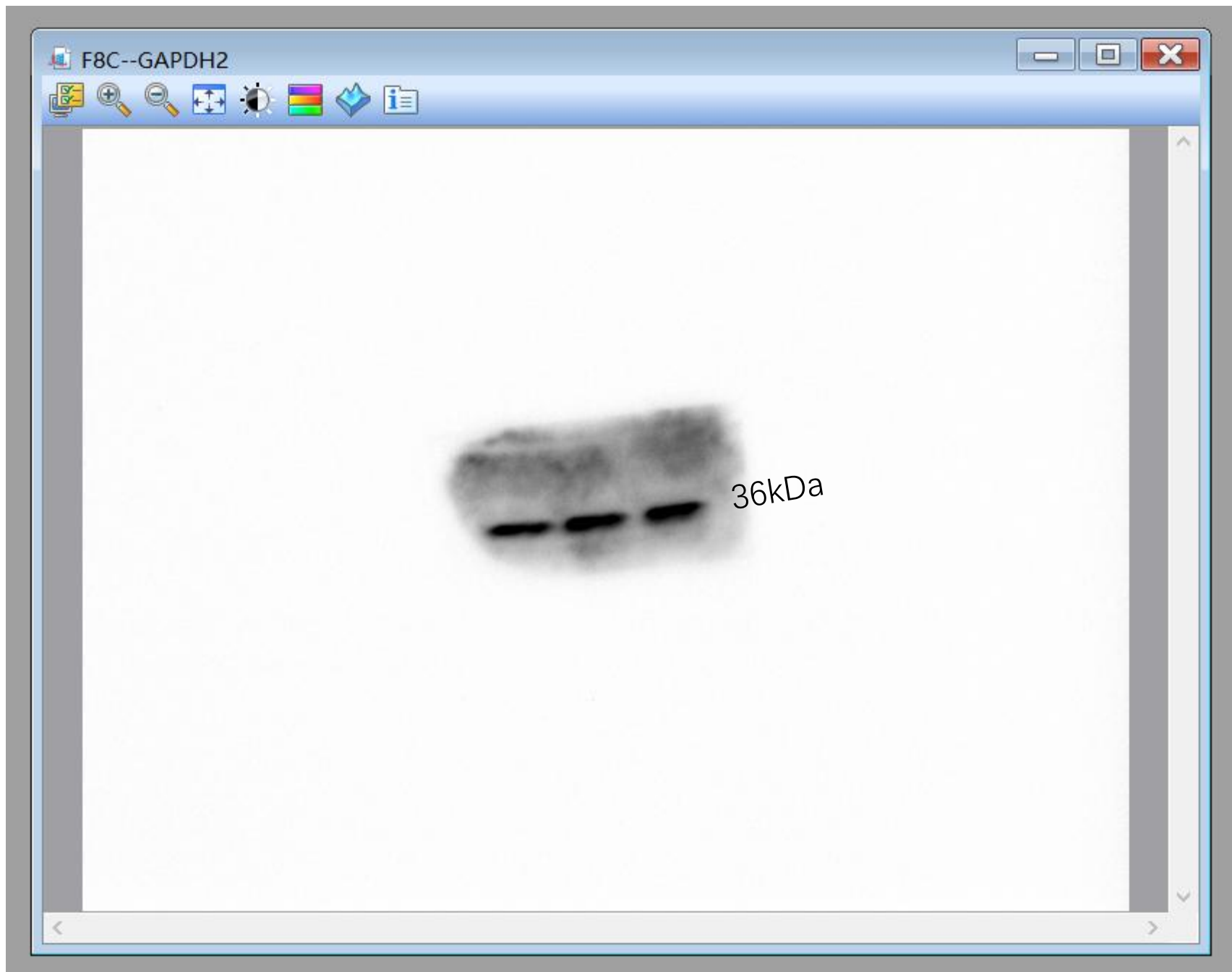

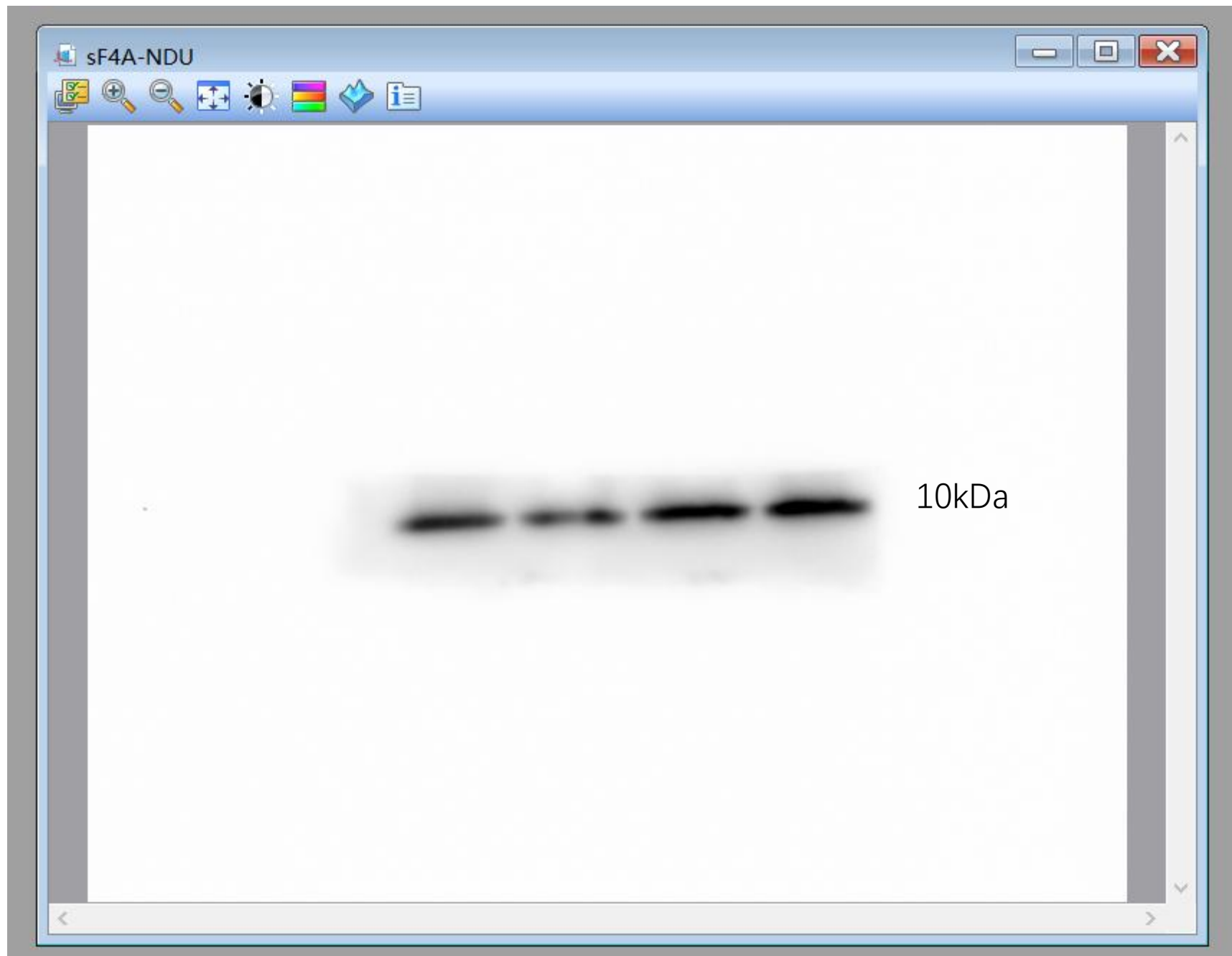

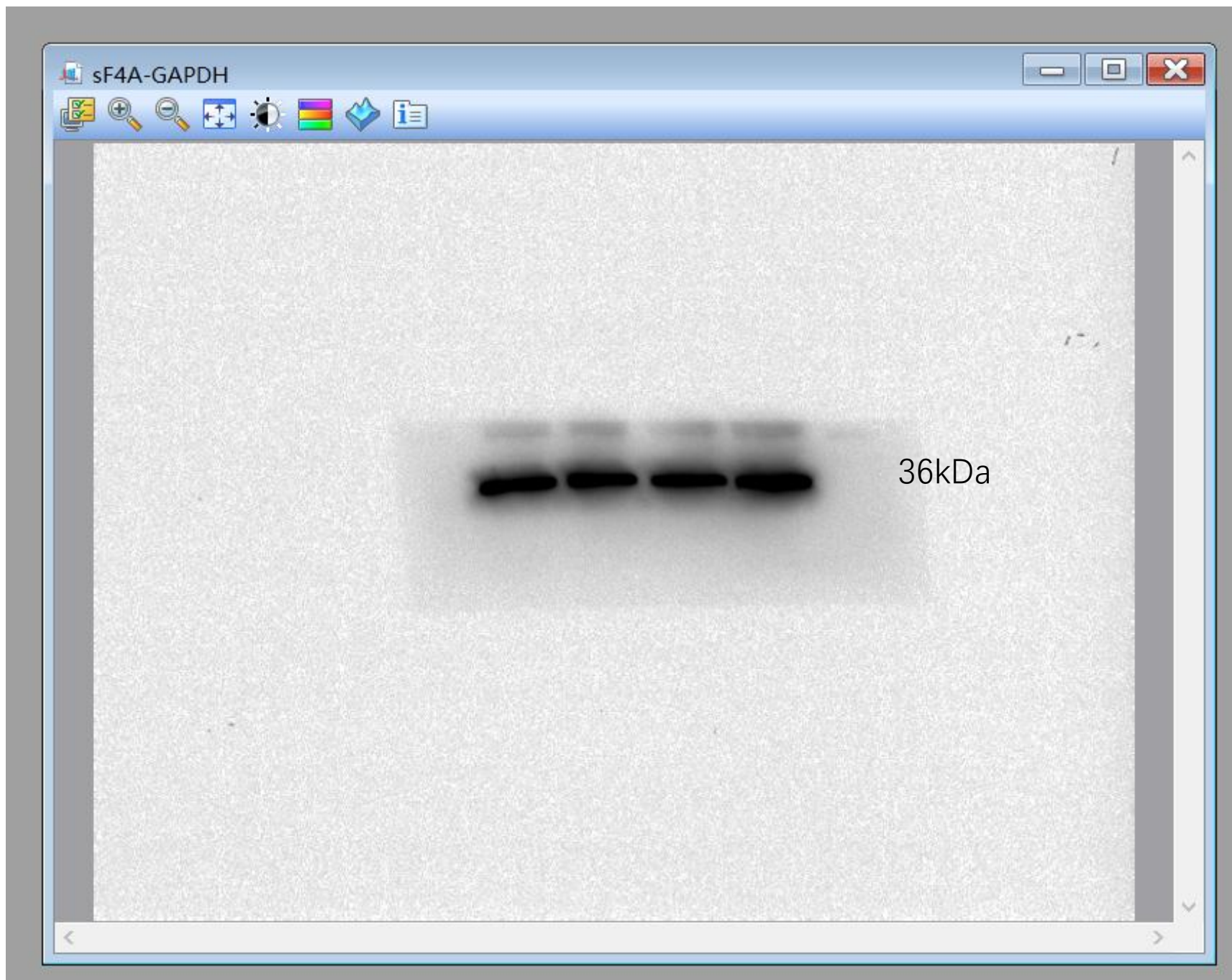

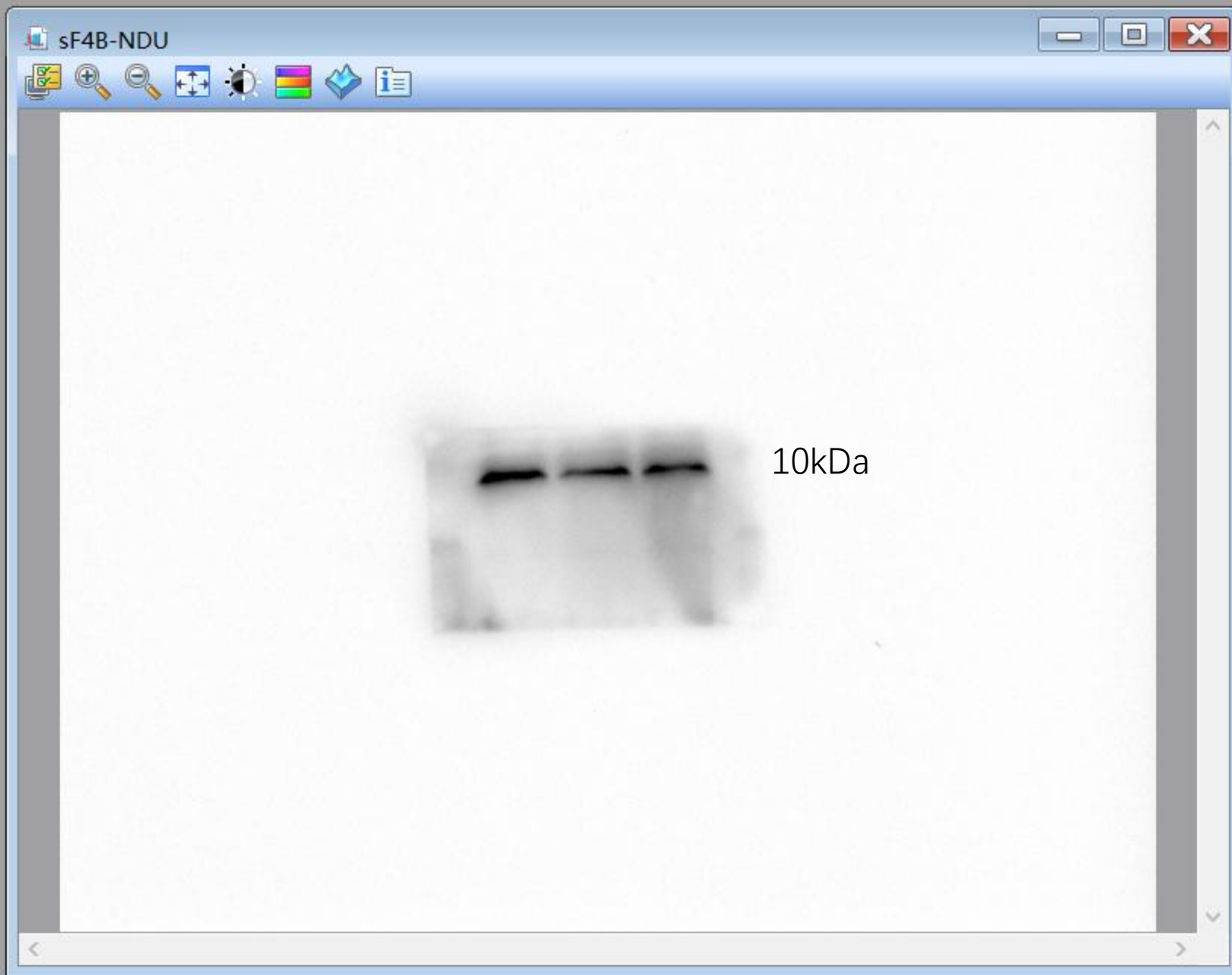

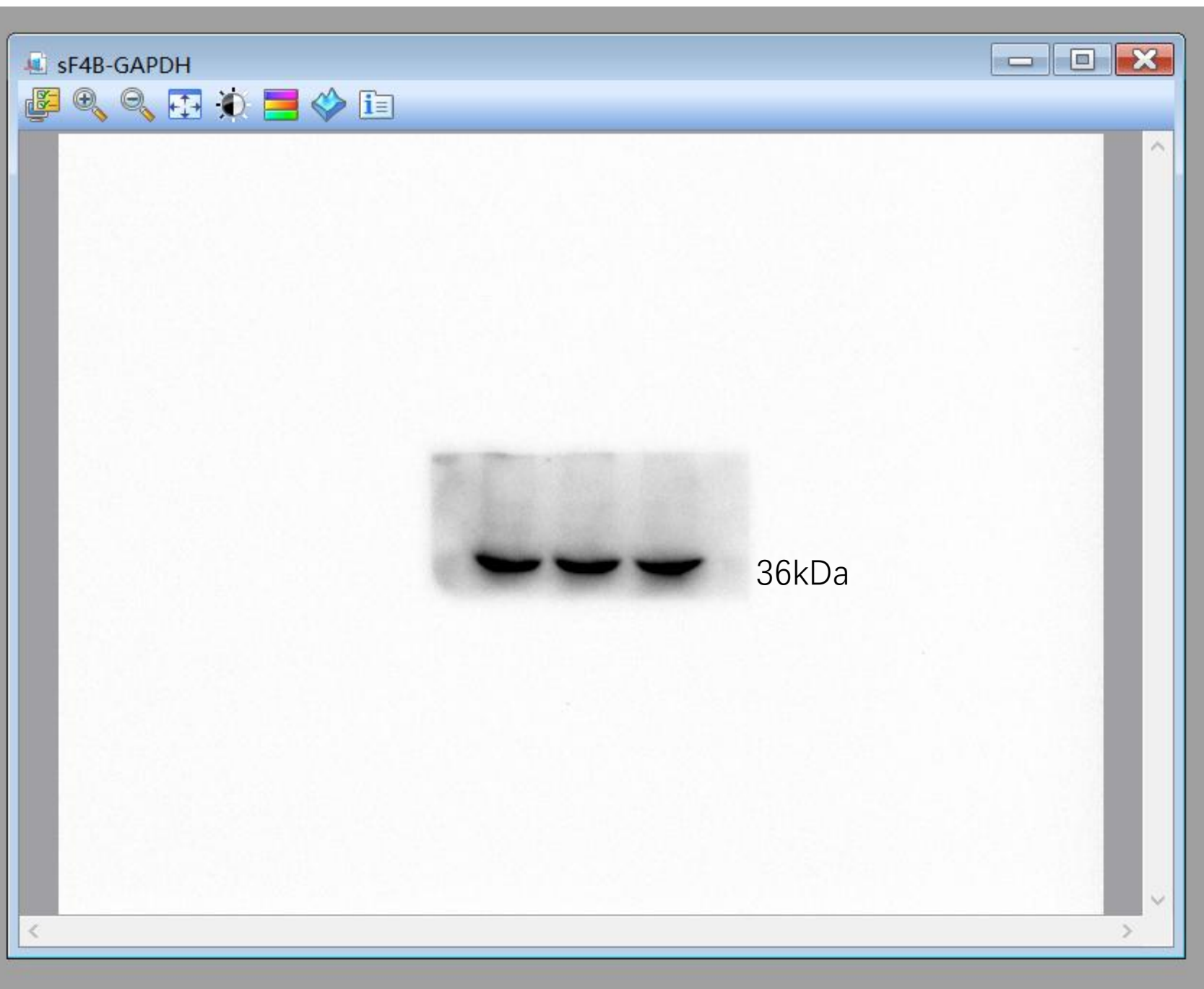

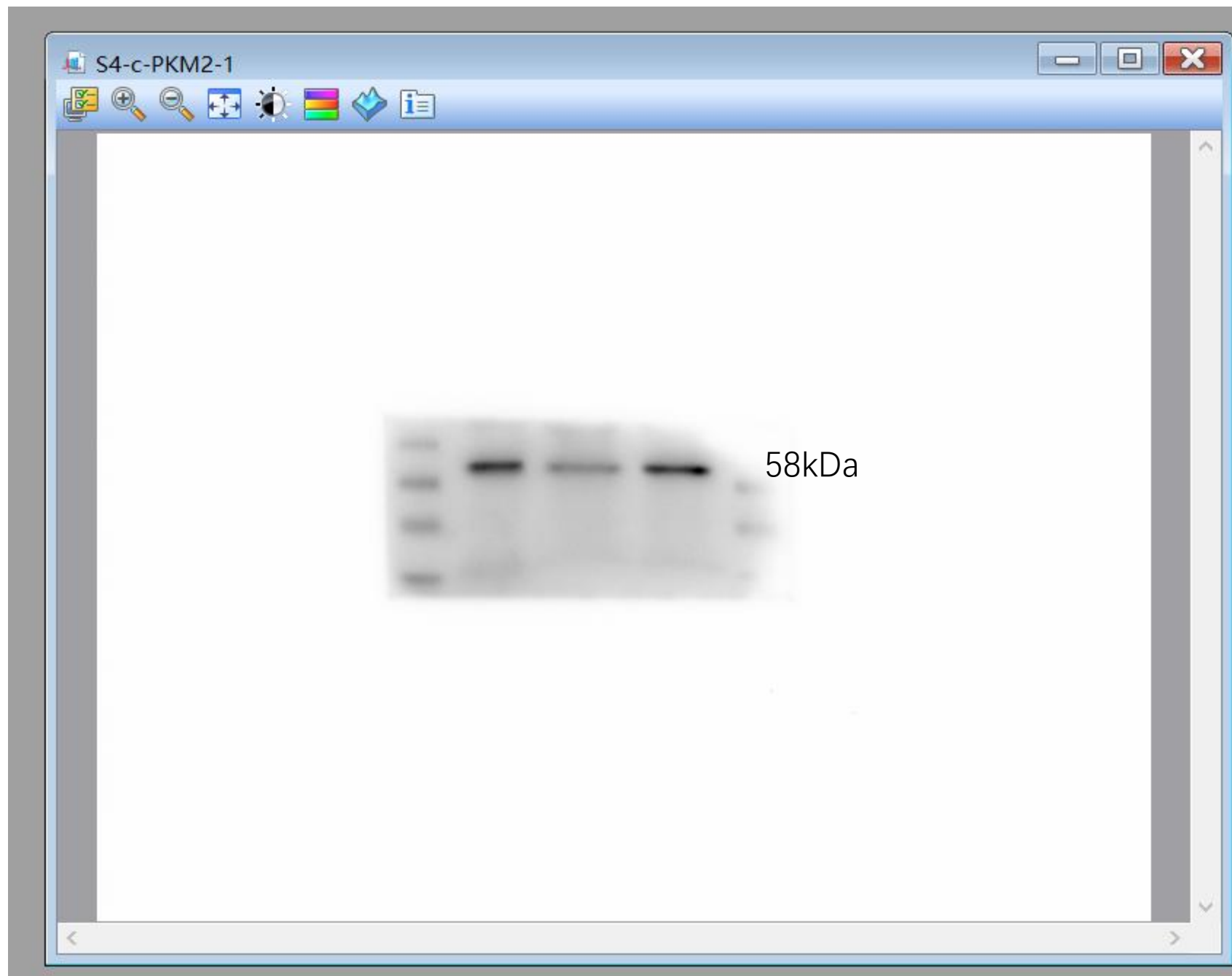

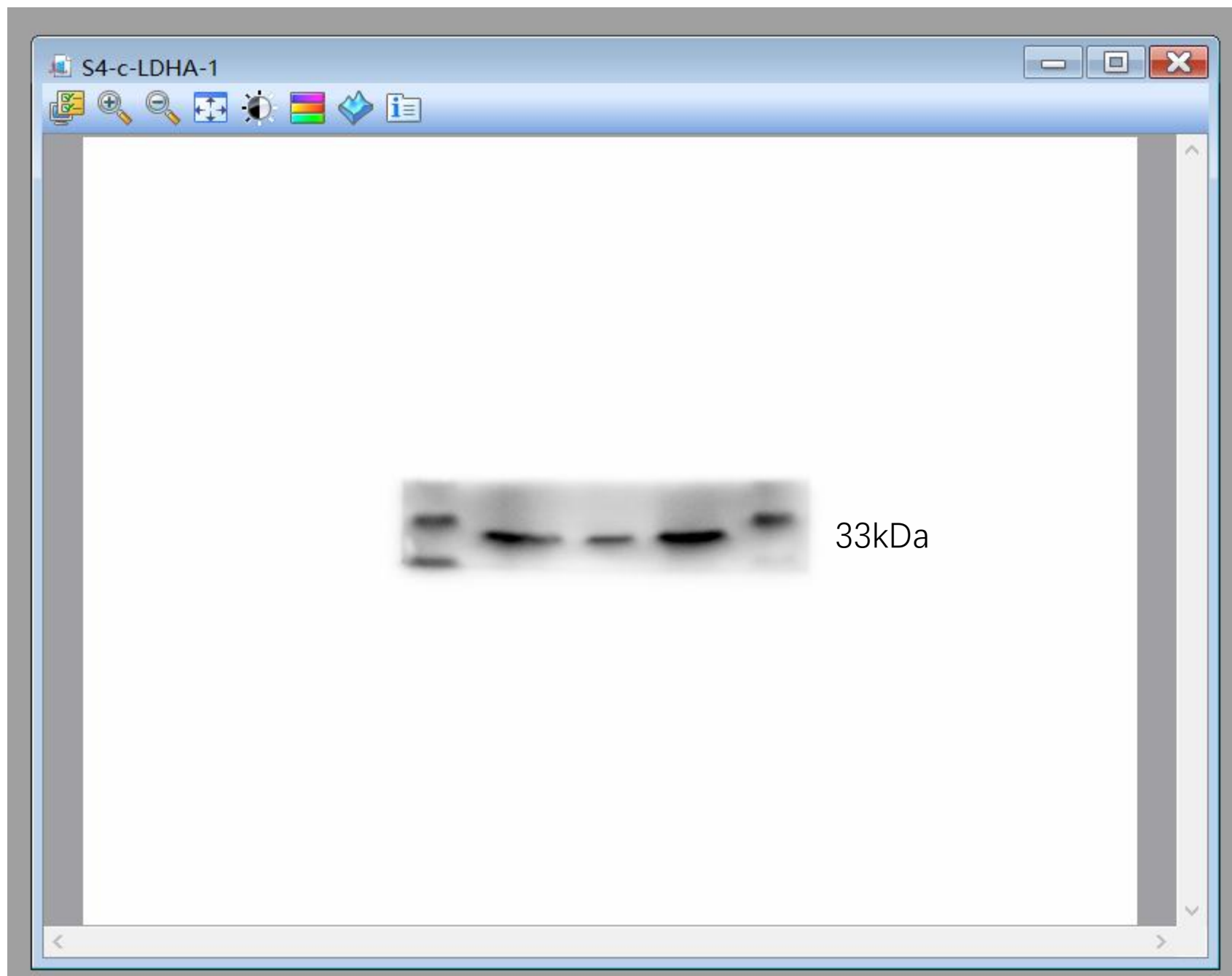

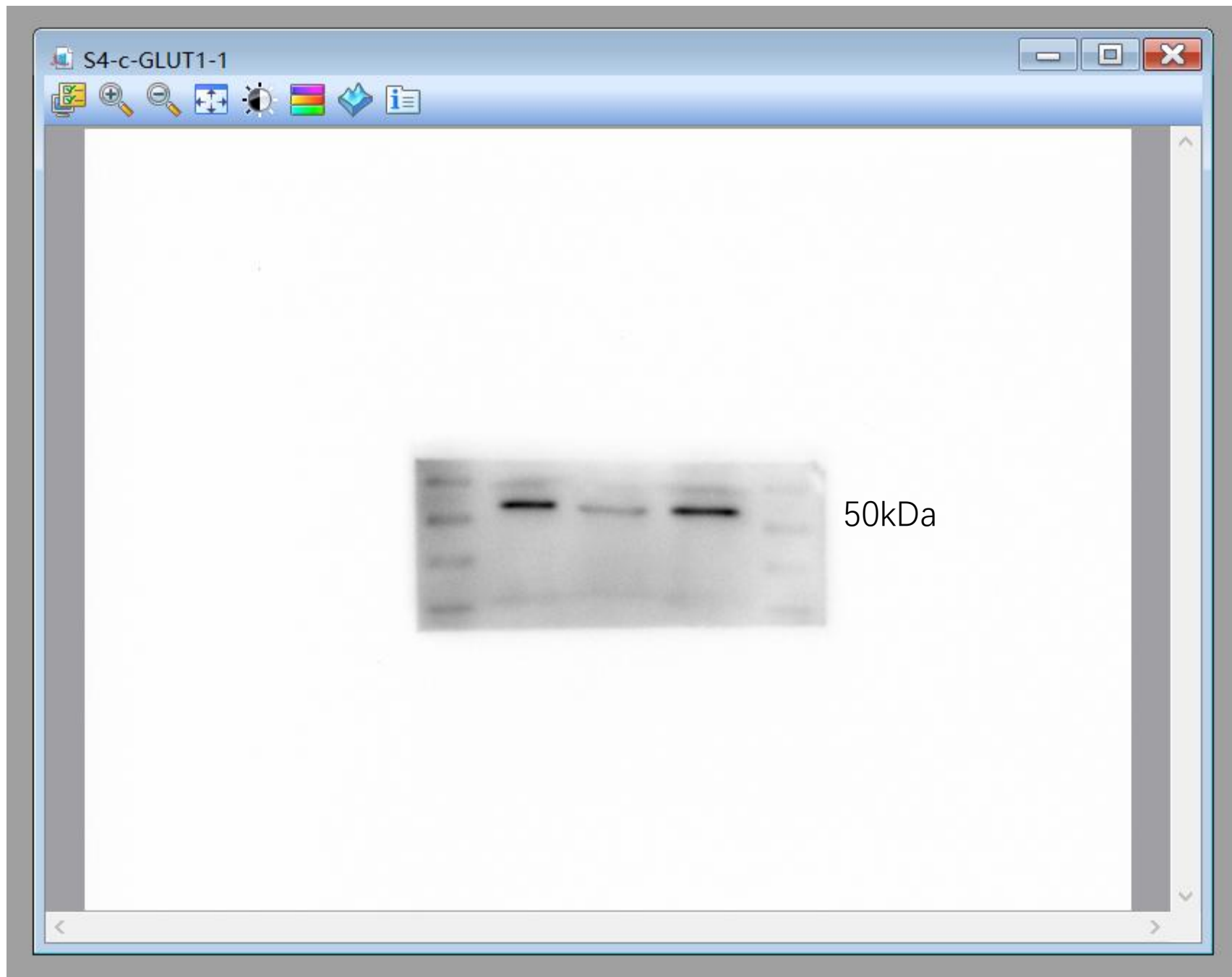

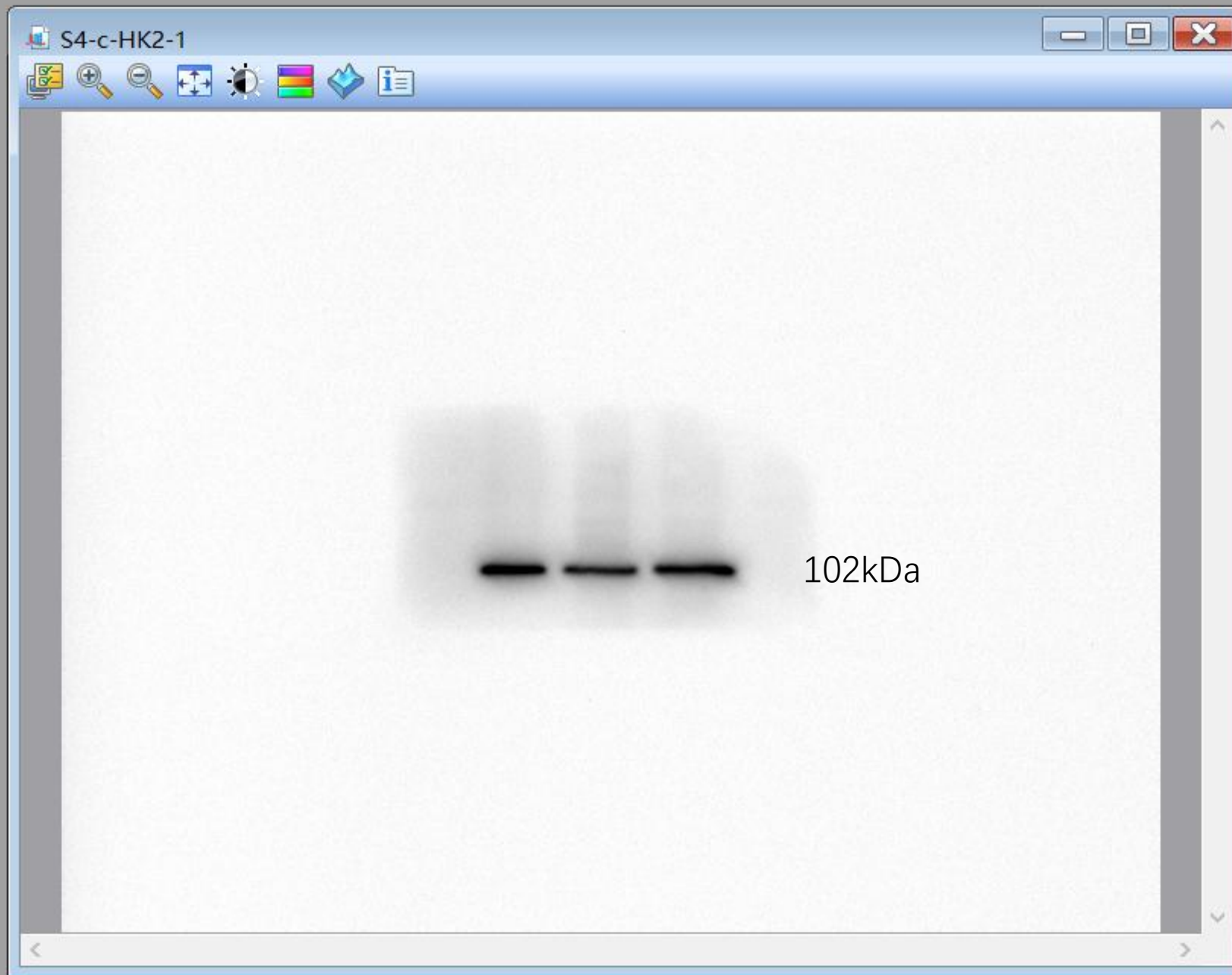

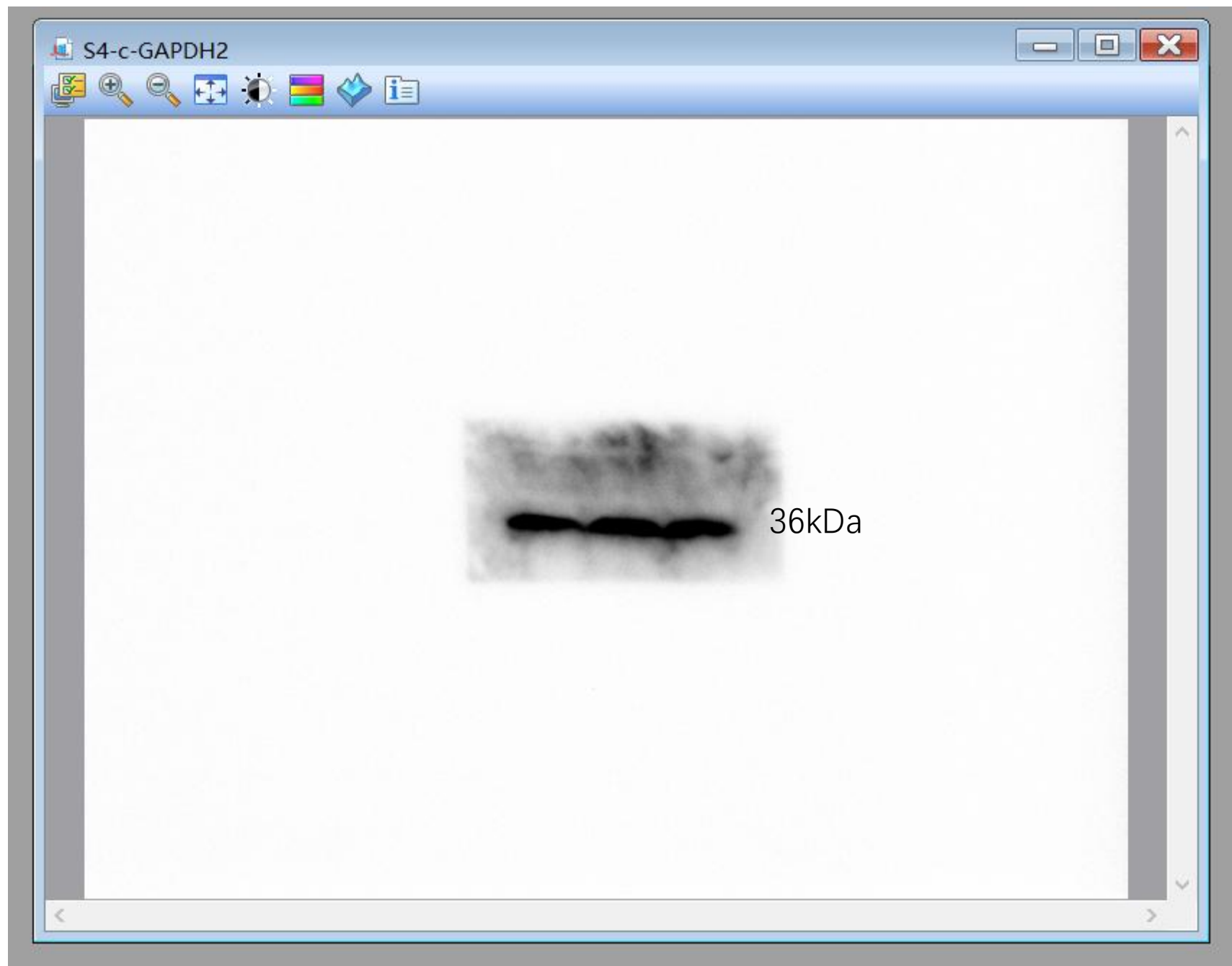

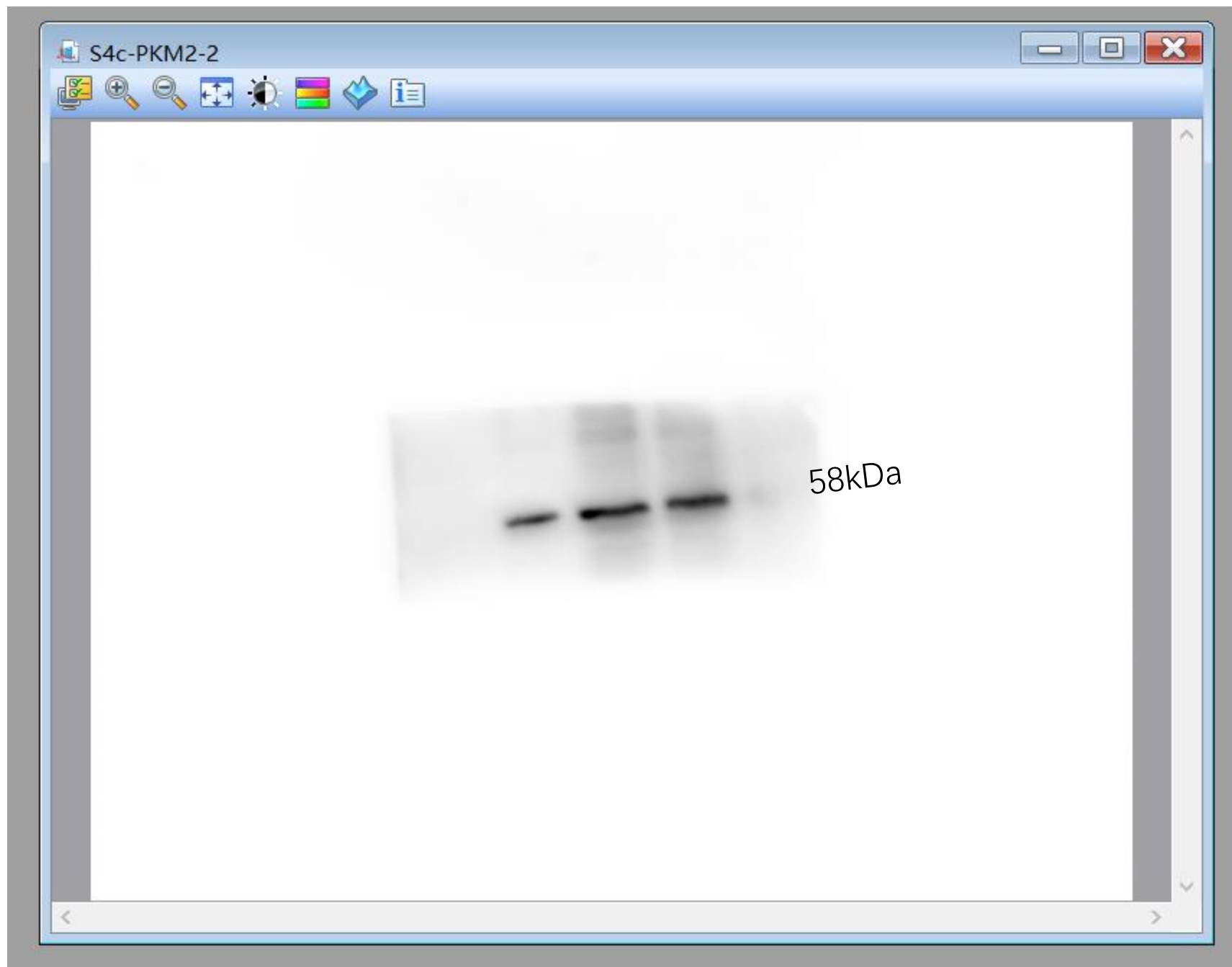

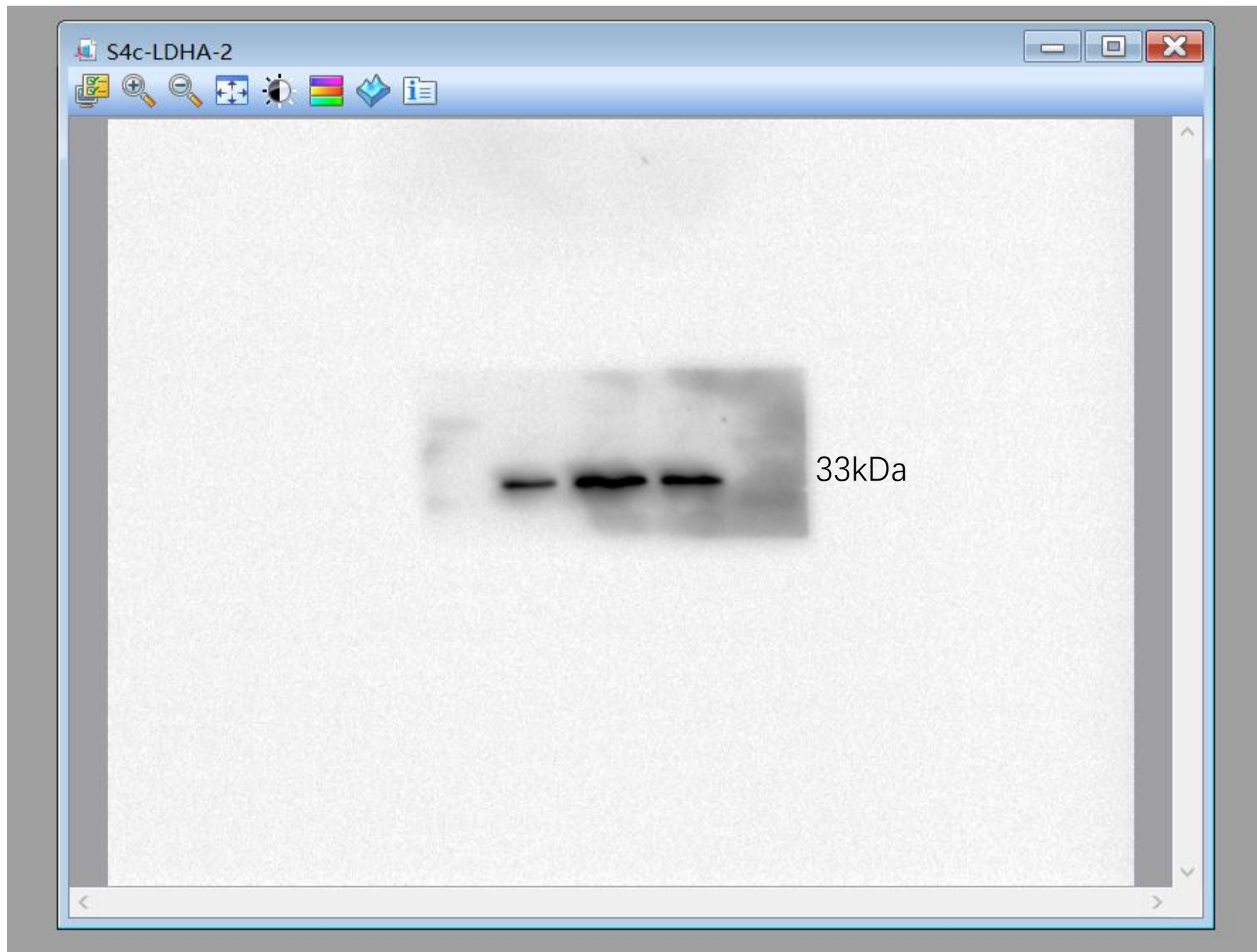

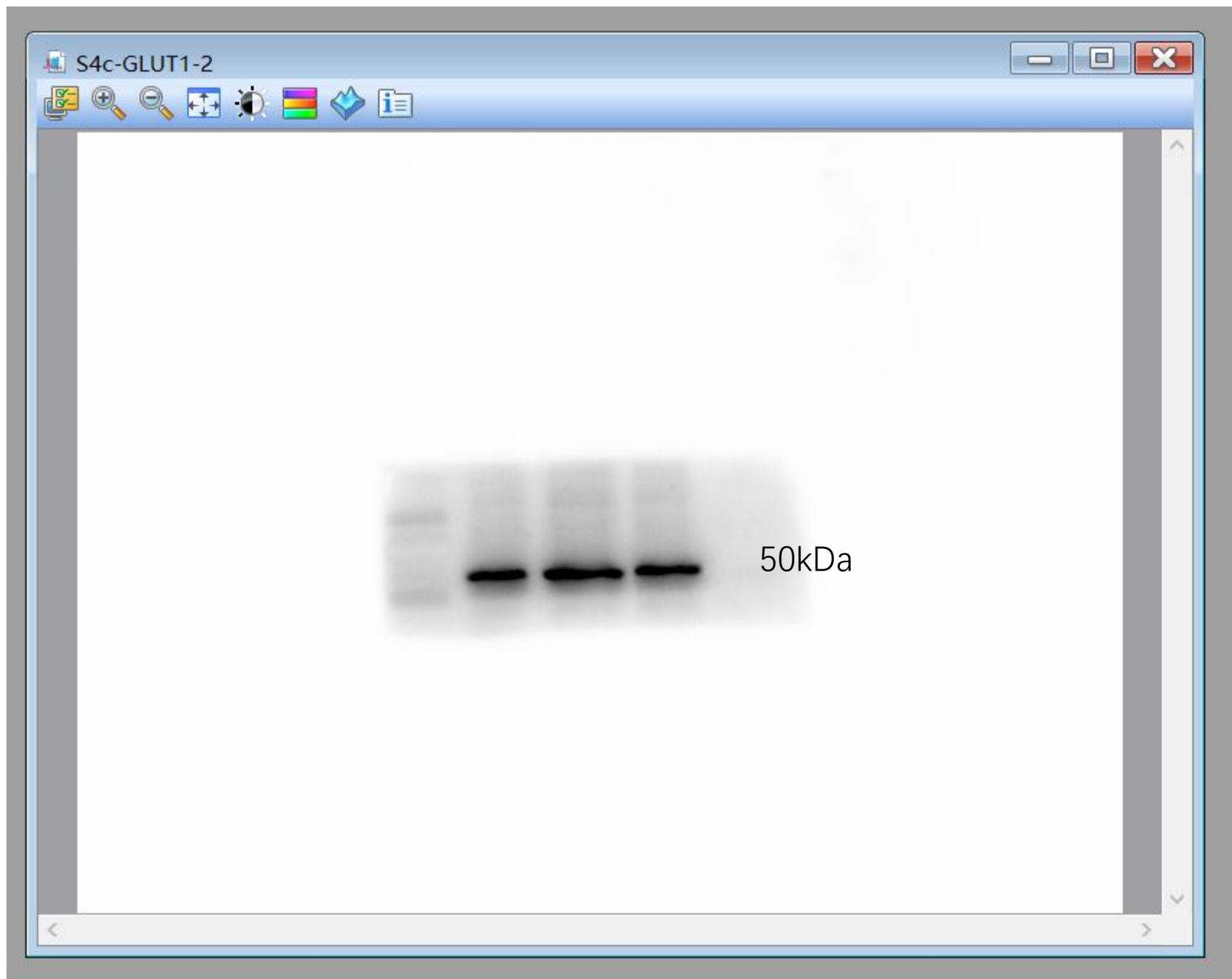

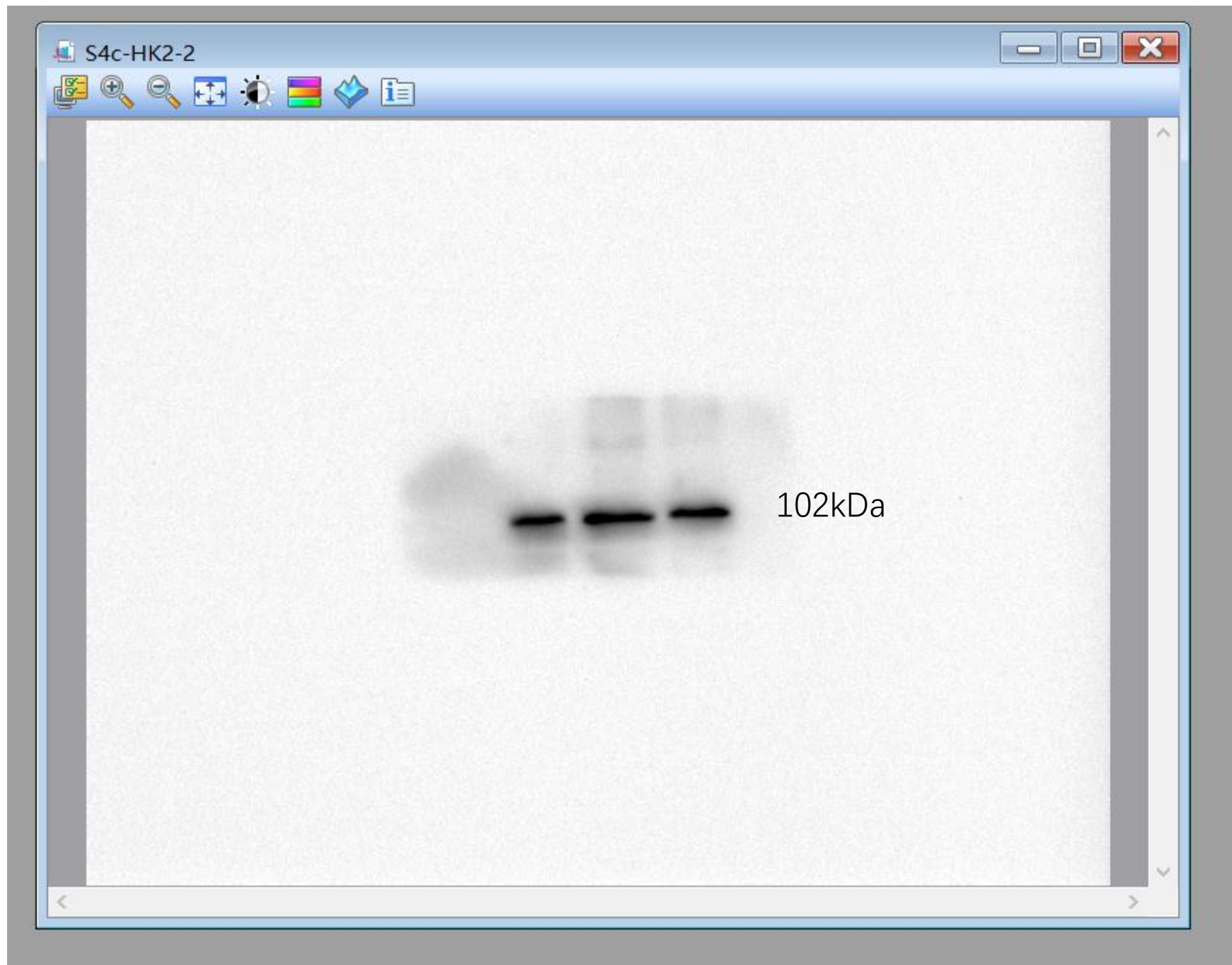

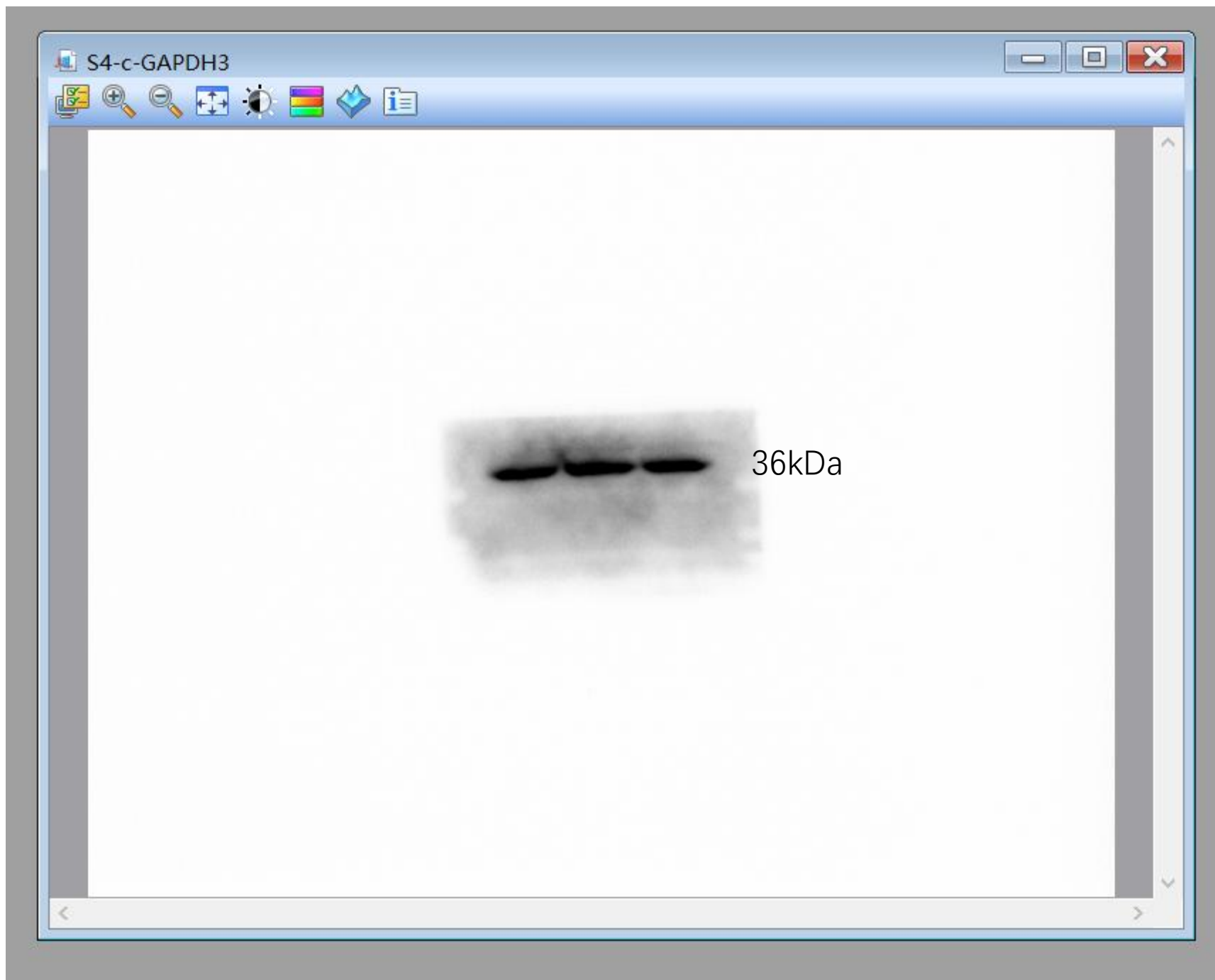

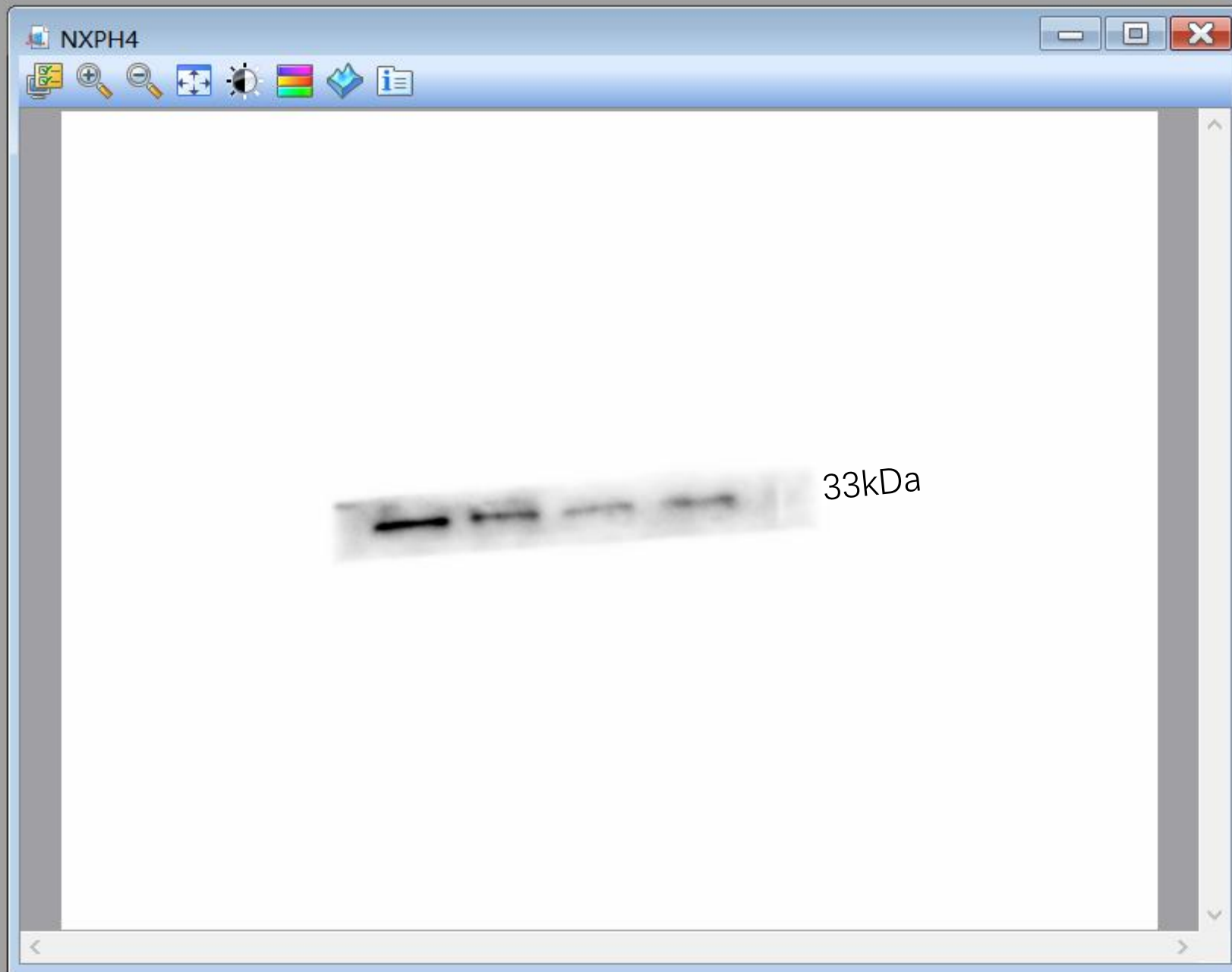

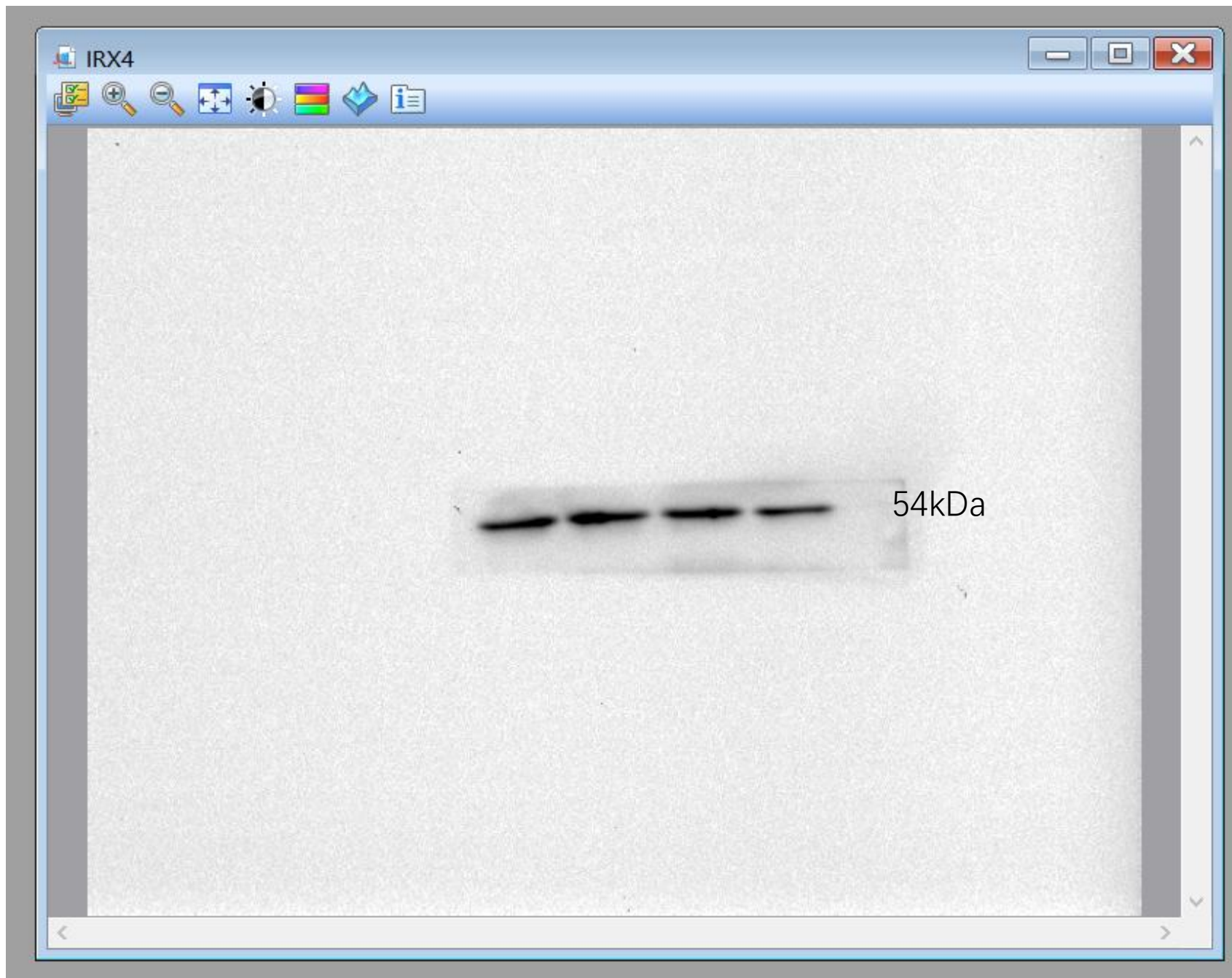

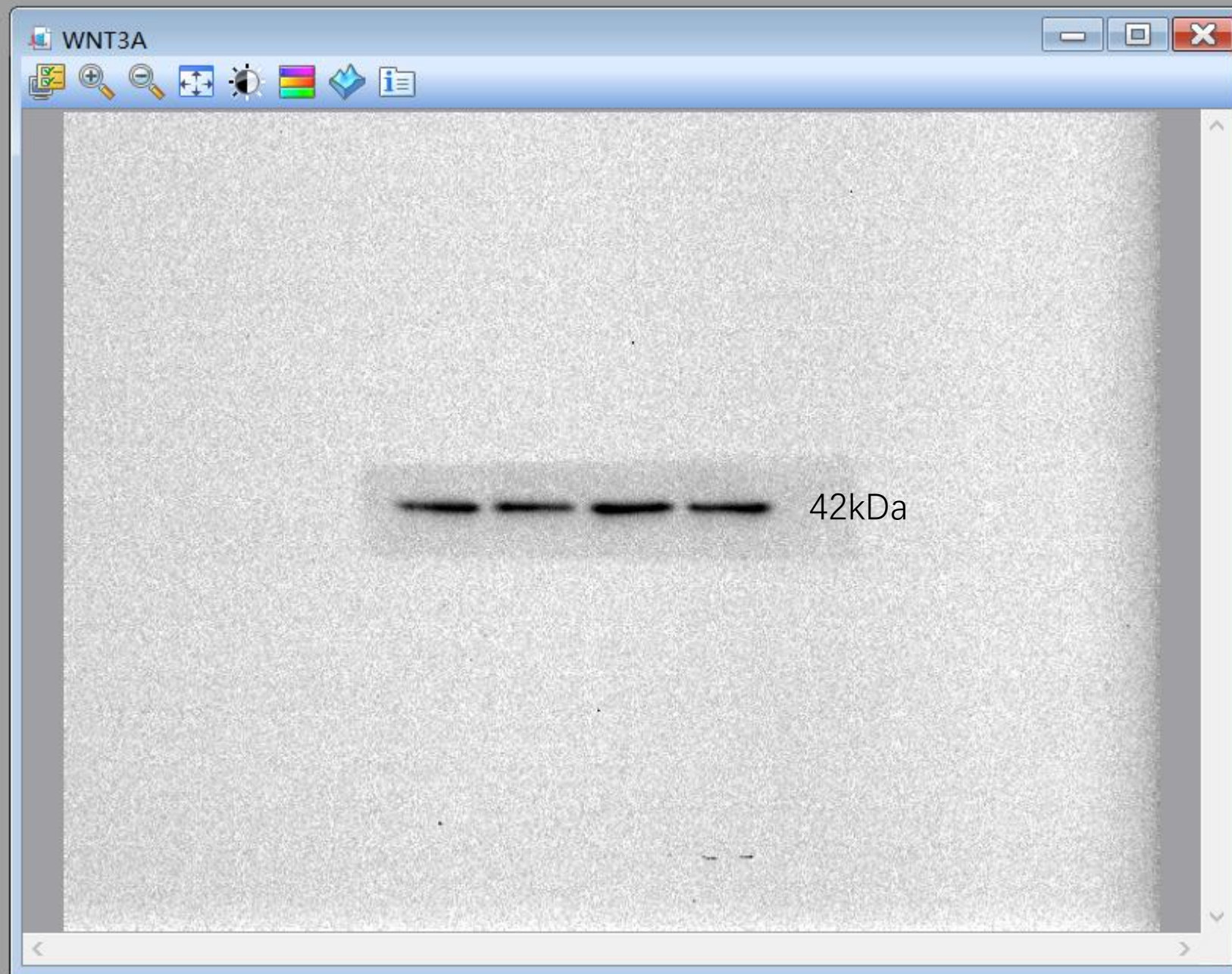

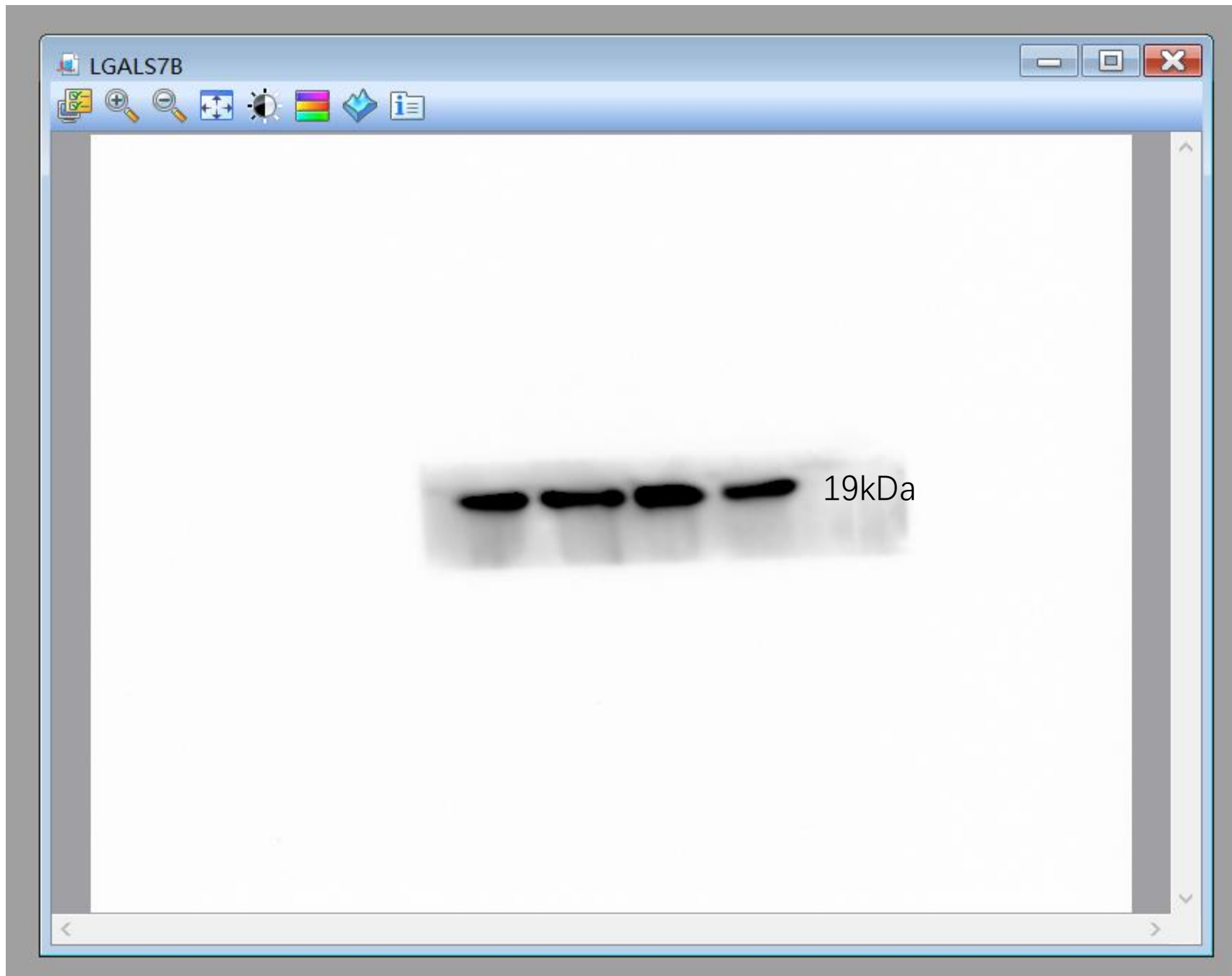

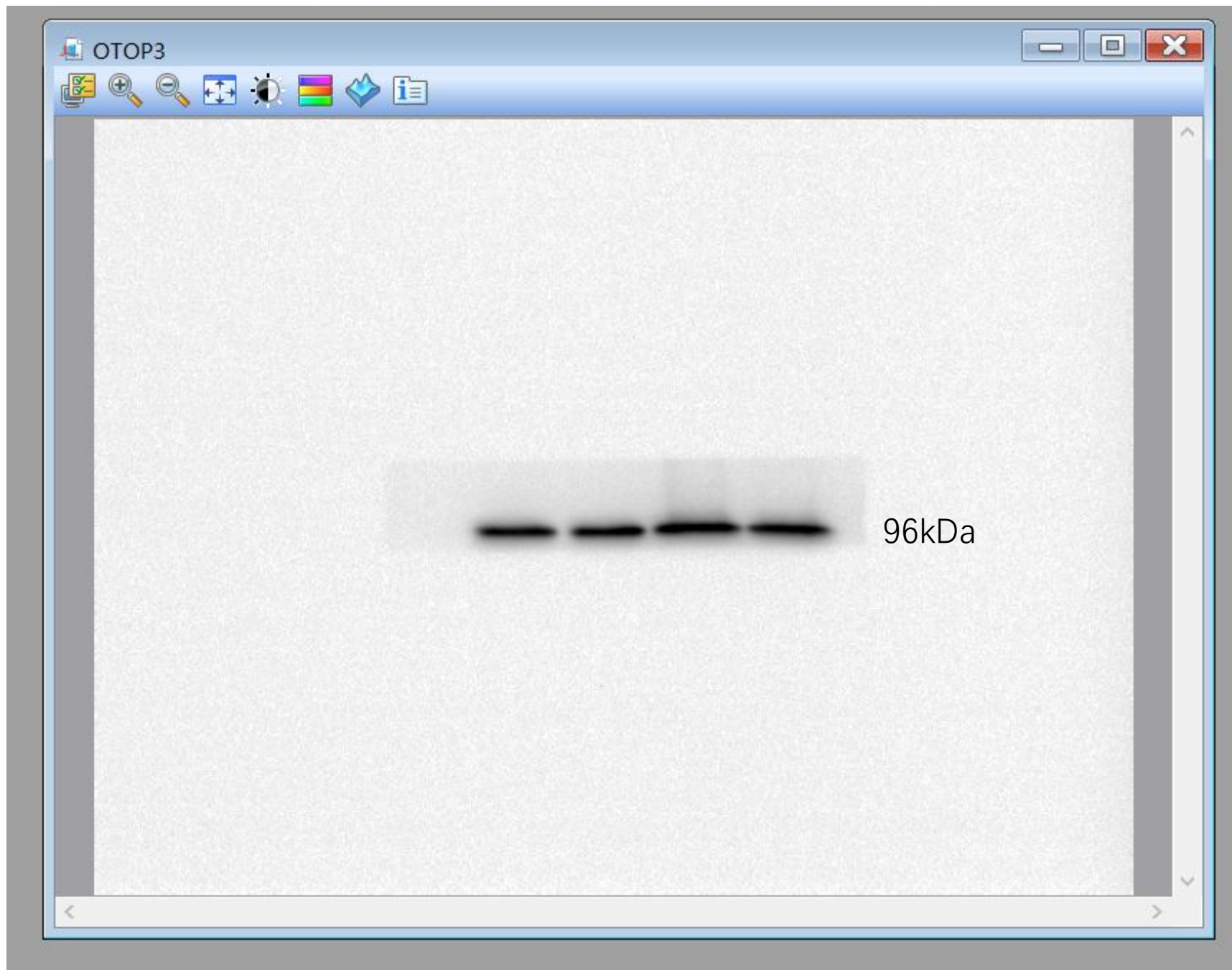

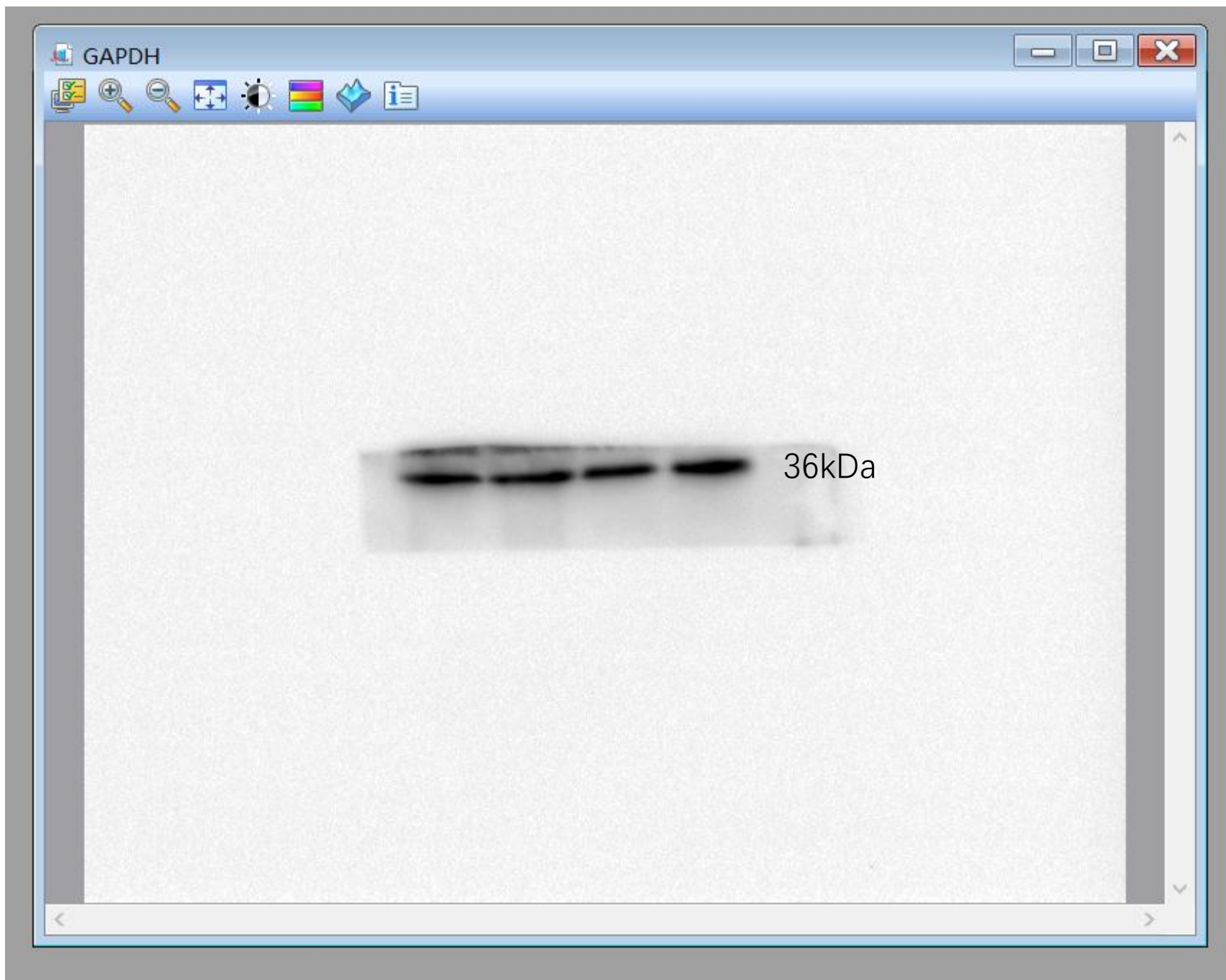

Supplement: Supplementary file 1 [file cancers-14-03782-s001.zip › Figure S6. All original western blot figures.pdf]
